# Supplementary figures and images for: Analysis of the genetic diversity of the coastal and island endangered plant species Elaeagnus macrophylla via conserved DNA-derived polymorphism marker (part 1 of 2)
Source: PeerJ. 2020 Jan 31;8:e8498. doi: 10.7717/peerj.8498 (PMC6996508; doi:10.7717/peerj.8498)

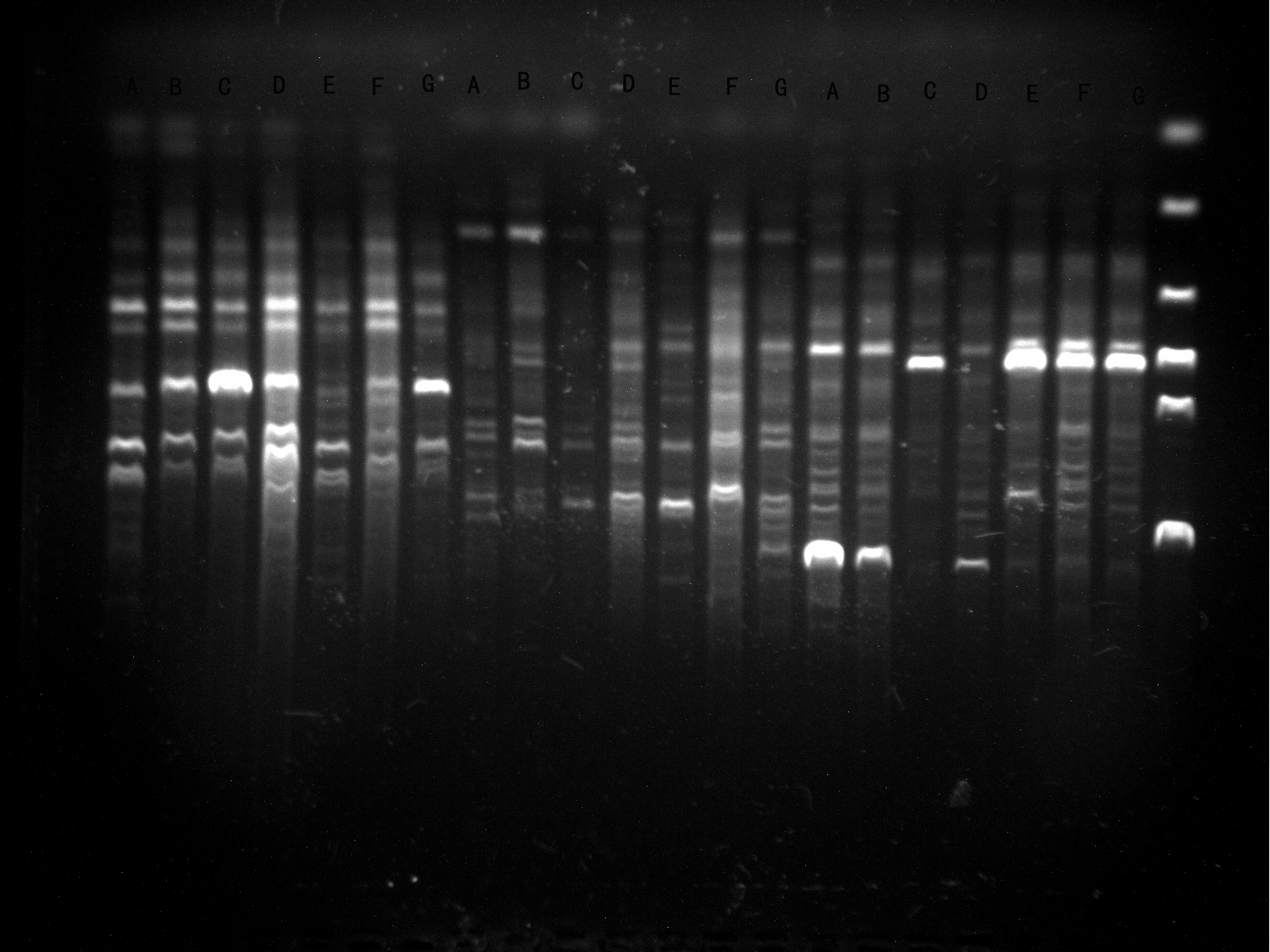

Supplement: Supplemental Information 2 — It can be seen that the three primer amplification bands are clear and specific and can be used for experiments. [file peerj-08-8498-s002.png]

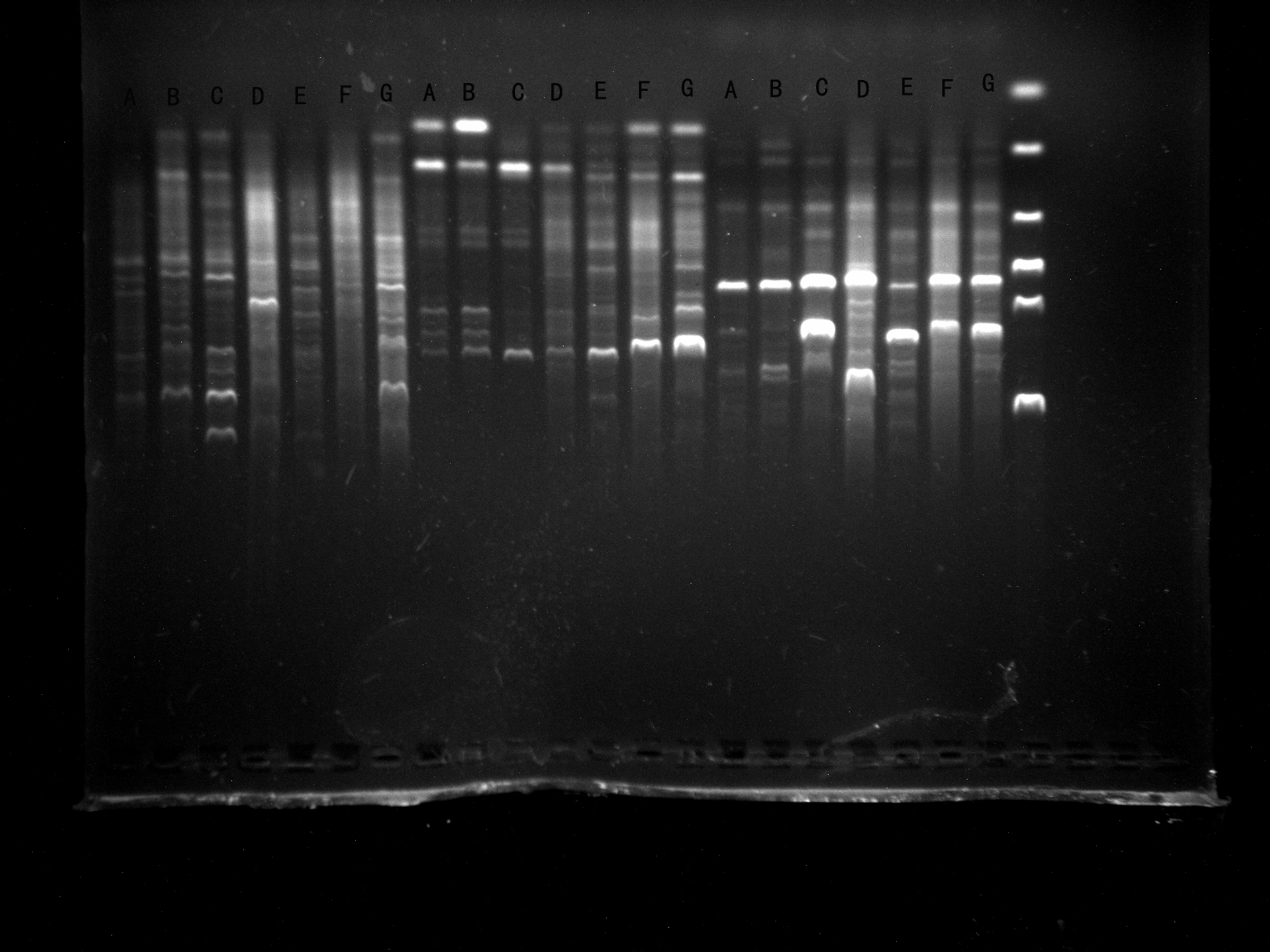

Supplement: Supplemental Information 3 — It can be seen that the MYB1, MYB2, ERF1 amplified bands are clear and specific and can be used for experiments. [file peerj-08-8498-s003.png]

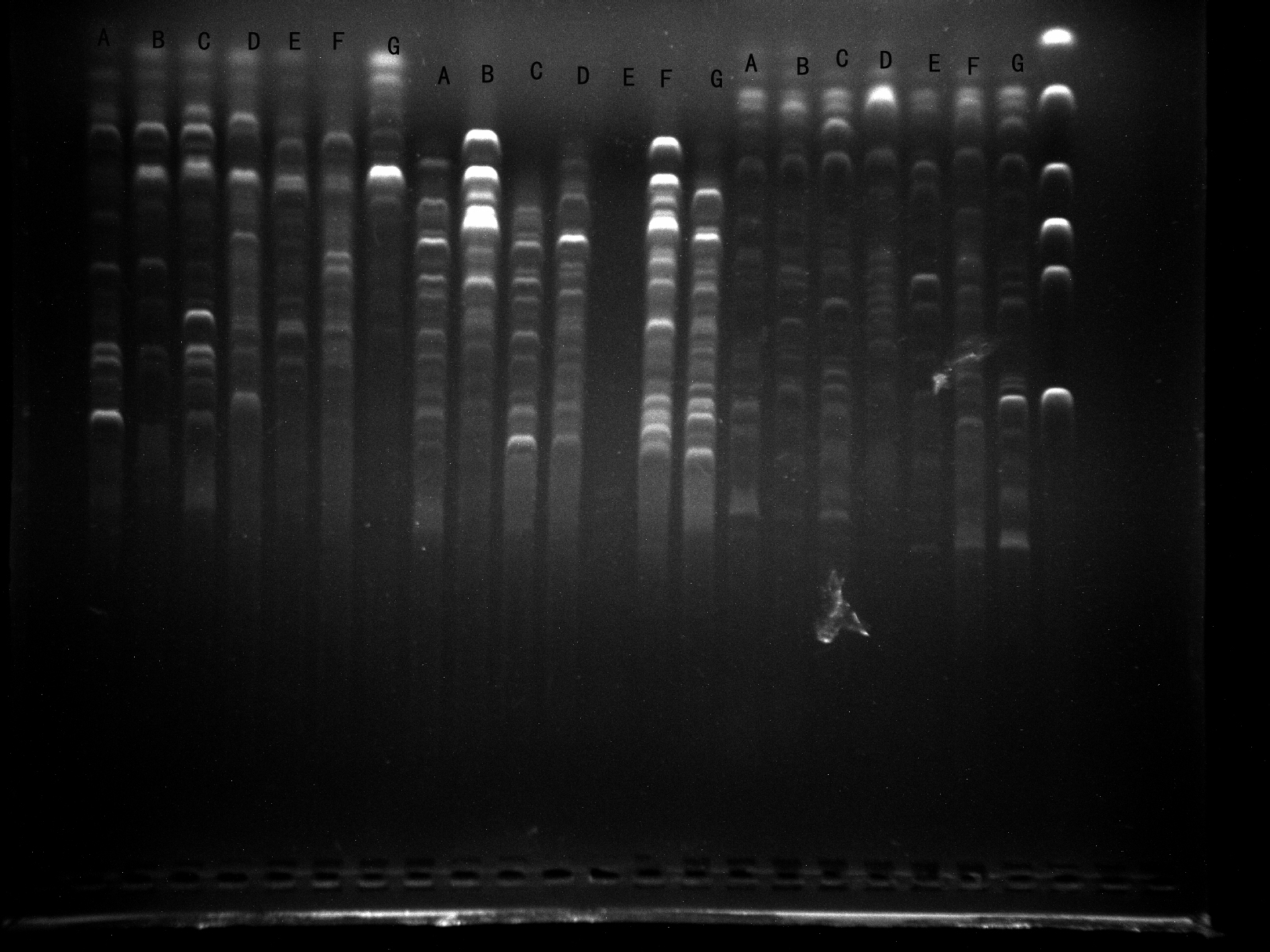

Supplement: Supplemental Information 4 — It can be seen that the WRKY-F1, WRKY-R1 amplified bands are clear and specific and can be used for experiments. Although the WRKY-R2 has a strip, the strip is weak and therefore not used. [file peerj-08-8498-s004.png]

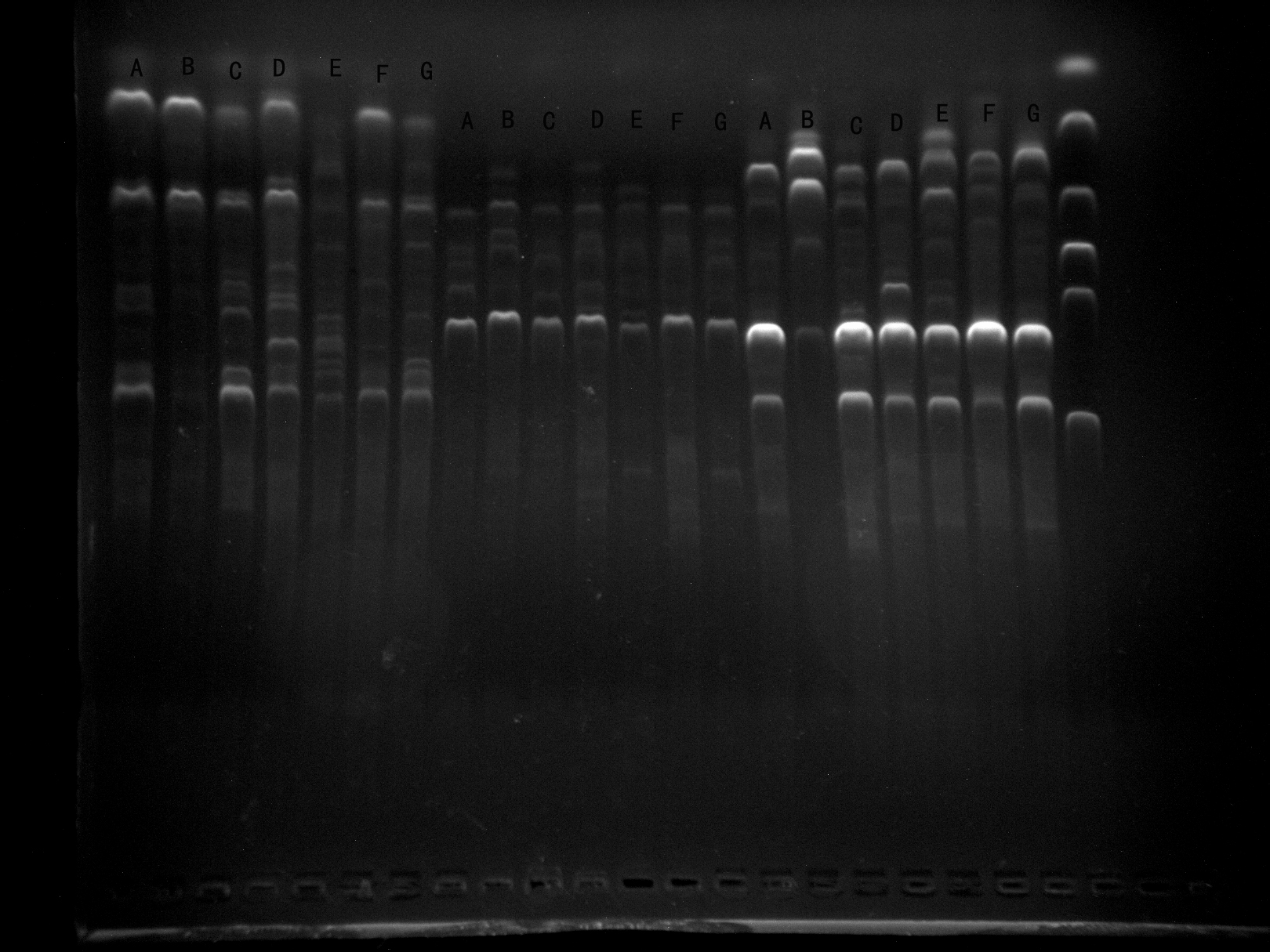

Supplement: Supplemental Information 5 — It can be seen that the WRKY-R3 amplified band is clear and specific, and can be used for experiments. WRKY-R2B, WRKY-R3B is not effective, so it is not used. [file peerj-08-8498-s005.png]

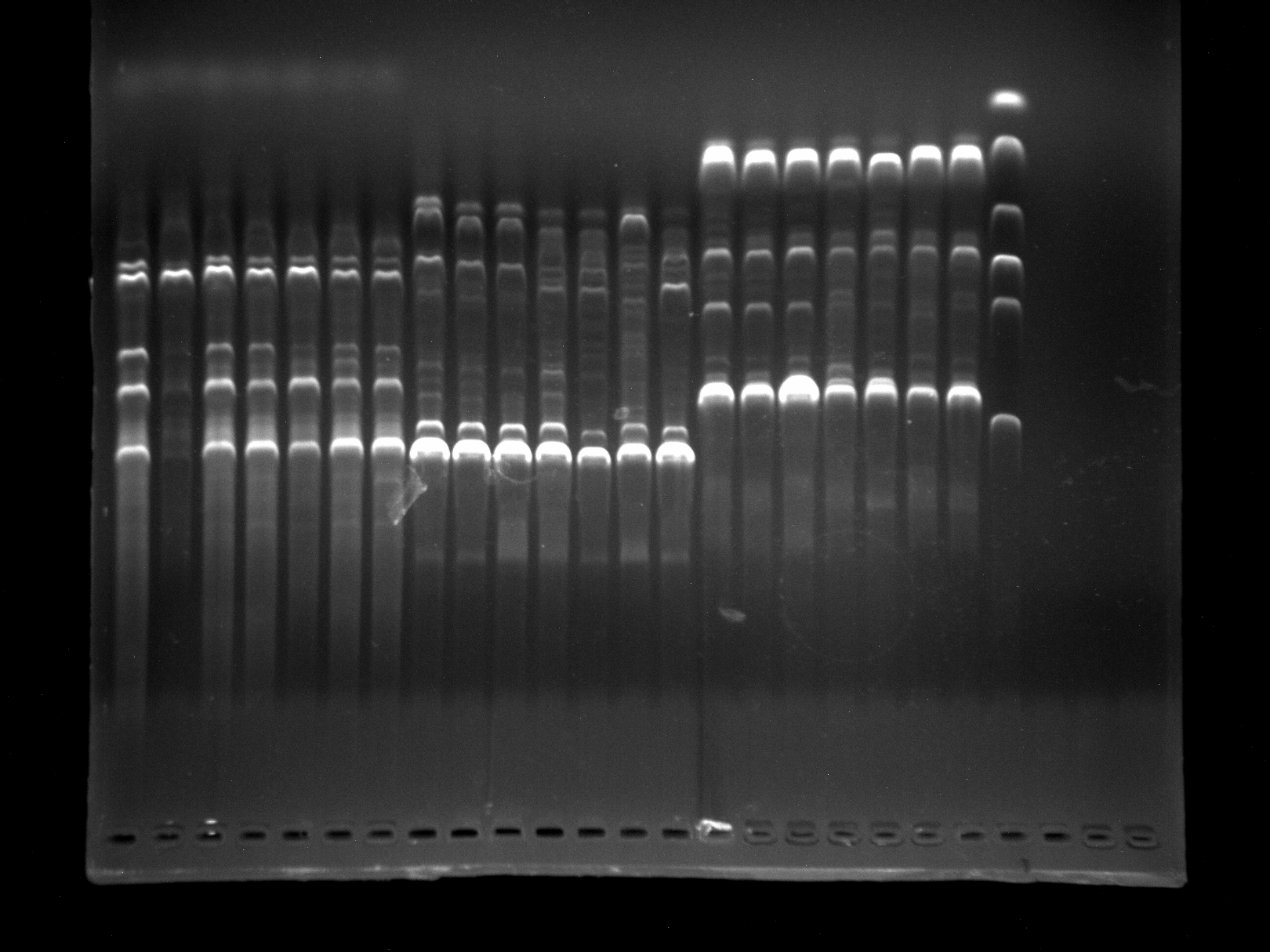

Supplement: Supplemental Information 6 — It can be seen that KNOX-2, KNOX-3, MADS-1 amplified bands are clear and specific and can be used for experiments. [file peerj-08-8498-s006.png]

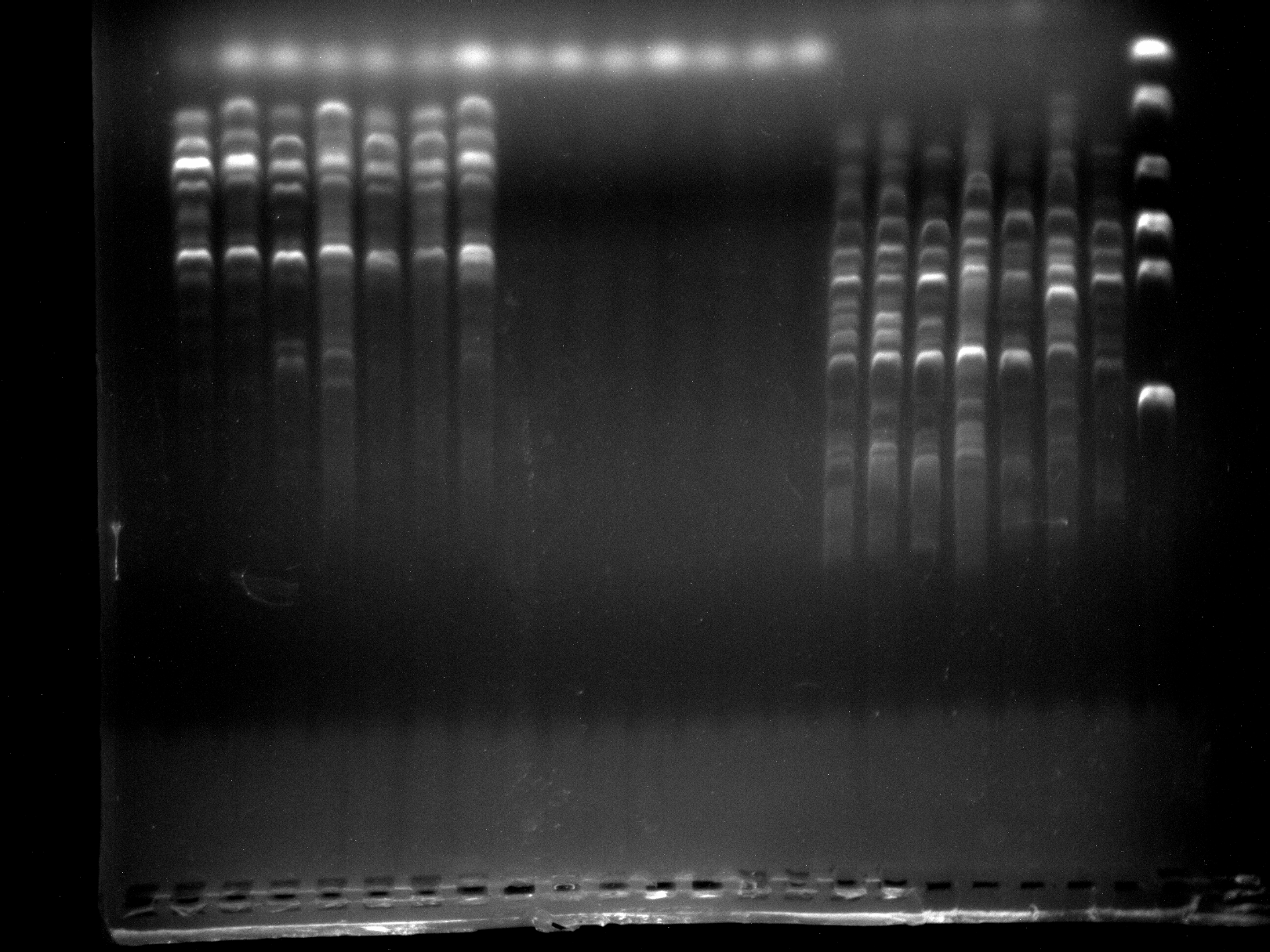

Supplement: Supplemental Information 7 — It can be seen that the ABP1-1, ABP1-3 amplified bands are clear and specific and can be used for experiments. ABP1-2 did not amplify the band and therefore could not be used. [file peerj-08-8498-s007.png]

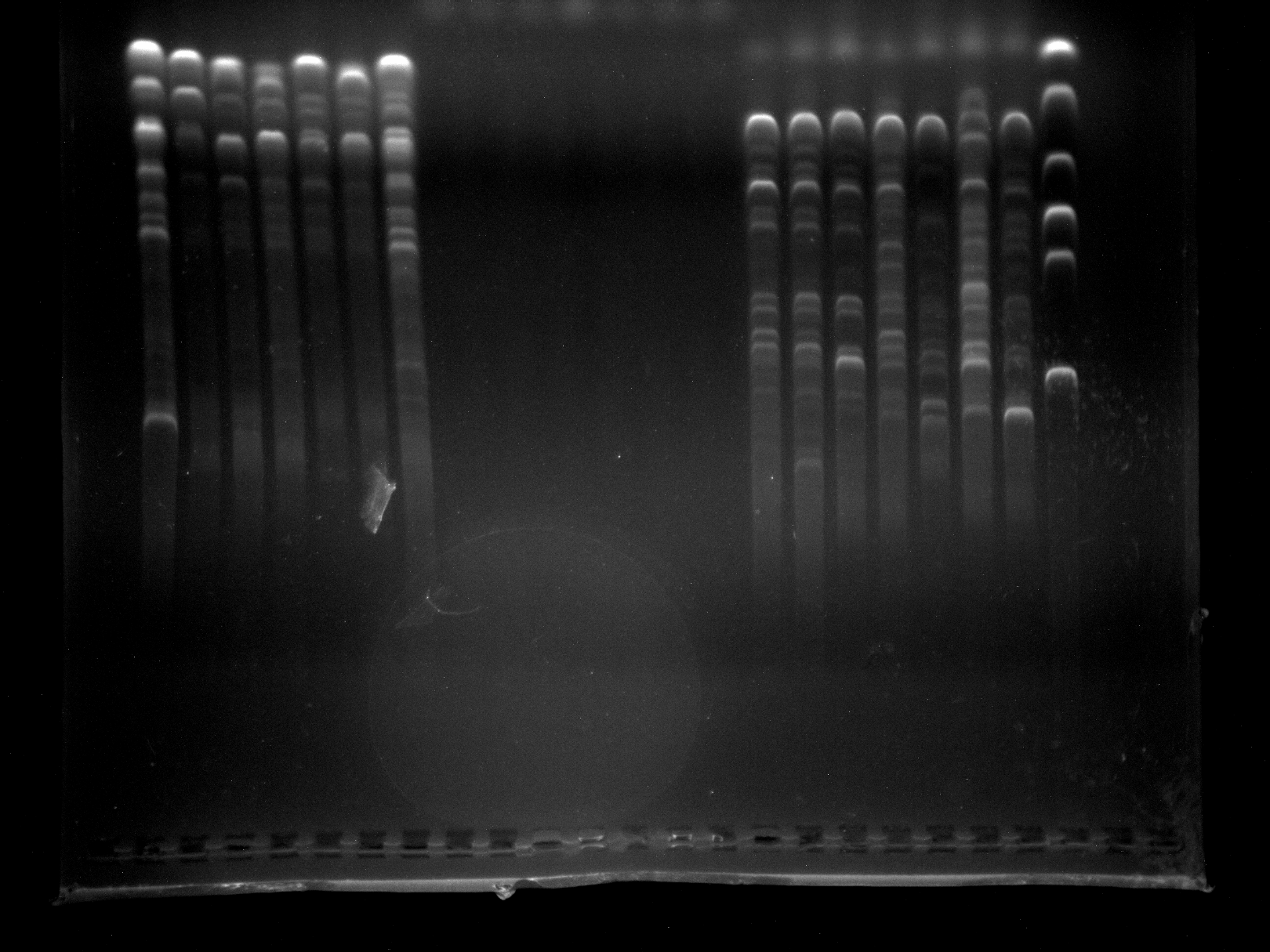

Supplement: Supplemental Information 8 — It can be seen that the MADS-4 amplification band is clear and specific and can be used for experiments. Although the MADS-2 has a band, it is not effective in use, and the MADS-3 does not amplify the band, so it is not used. [file peerj-08-8498-s008.png]

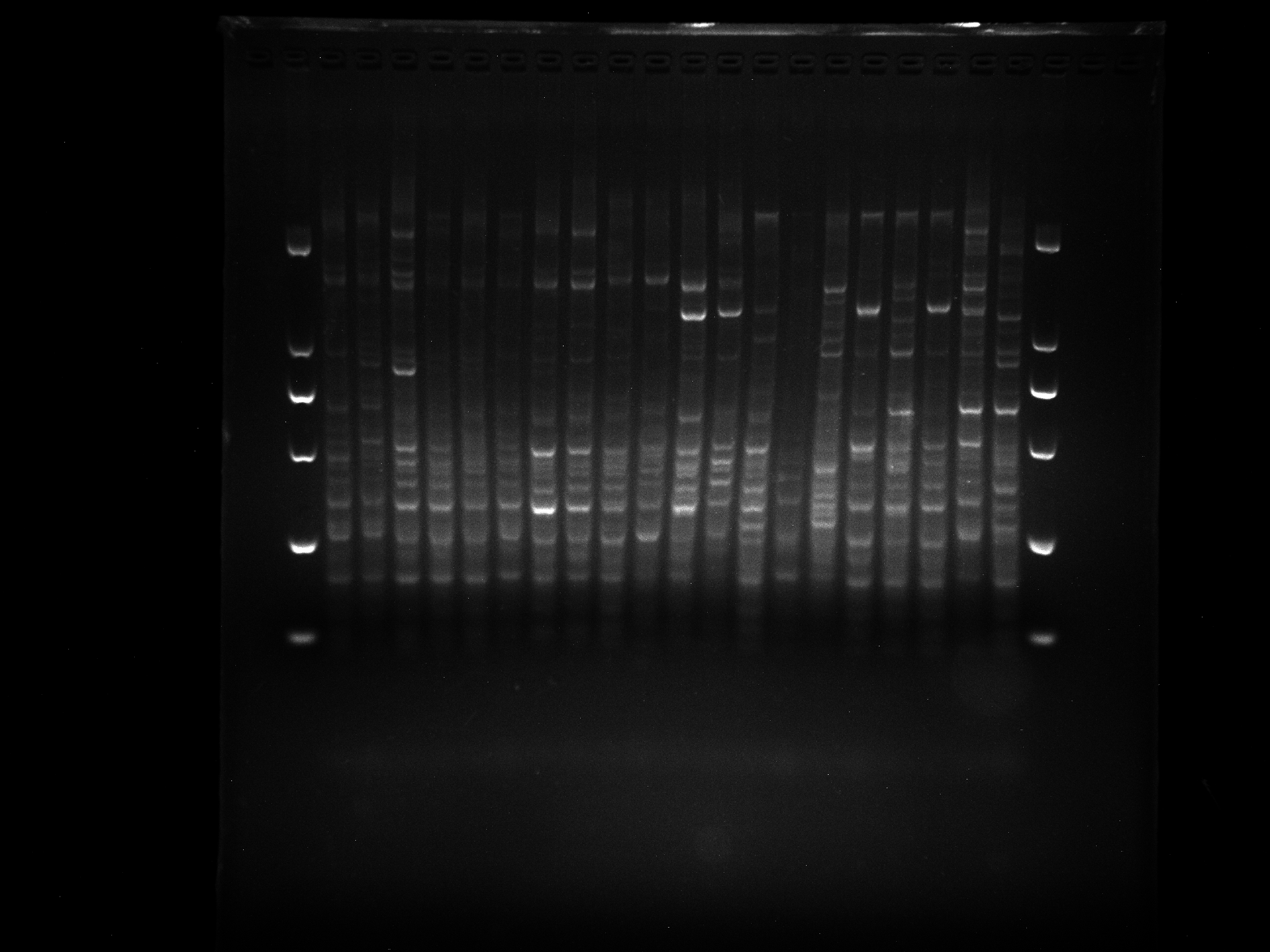

Supplement: Supplemental Information 9 — Amplification results of WRKY-F1 on LGD1-8 and DRS9-20. [file peerj-08-8498-s009.png]

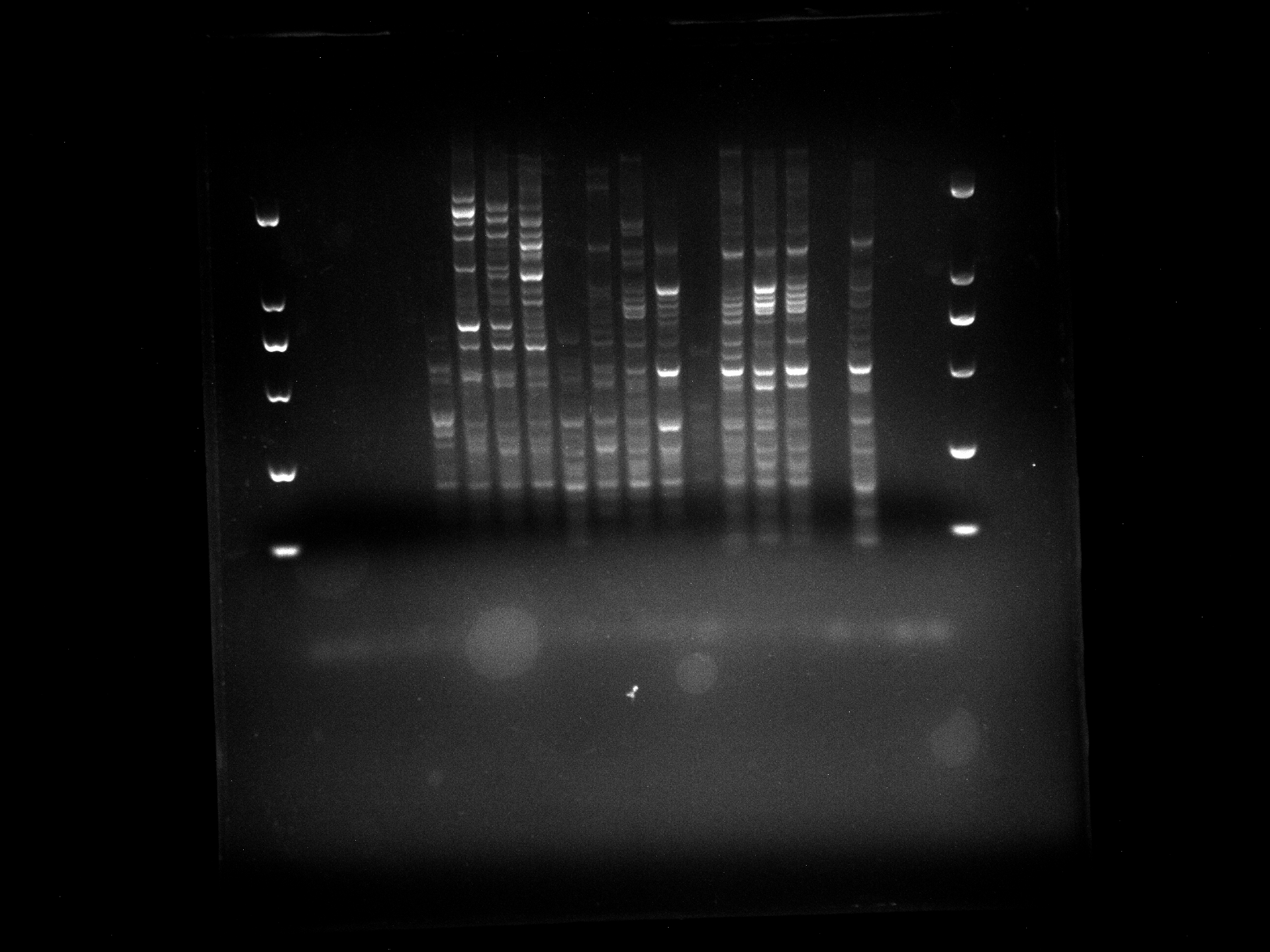

Supplement: Supplemental Information 10 — Amplification results of WRKY-F1 on DRS21-28, NJD1-12. [file peerj-08-8498-s010.png]

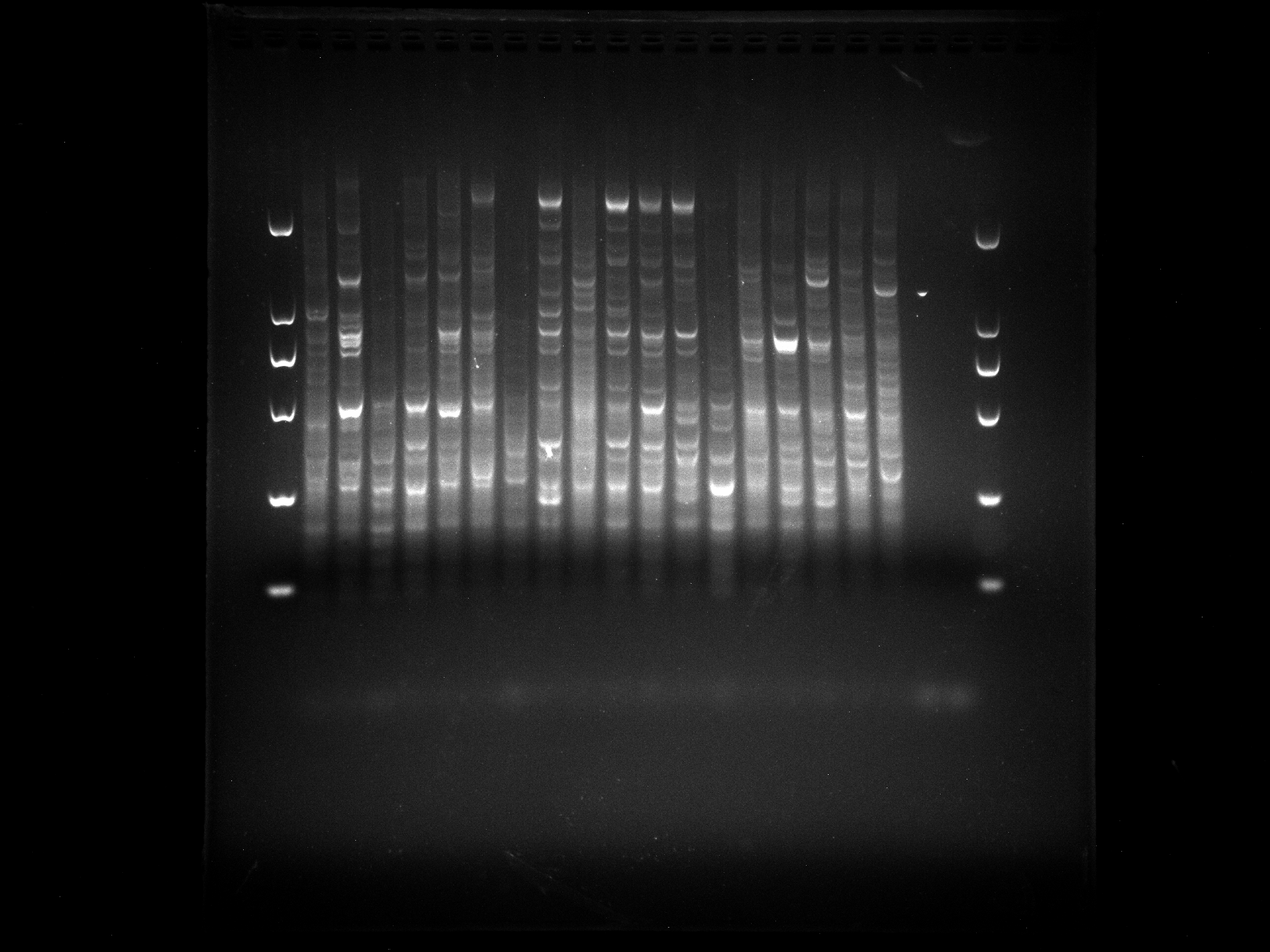

Supplement: Supplemental Information 11 — Amplification results of WRKY-F1 on NJD13-NJD32. [file peerj-08-8498-s011.png]

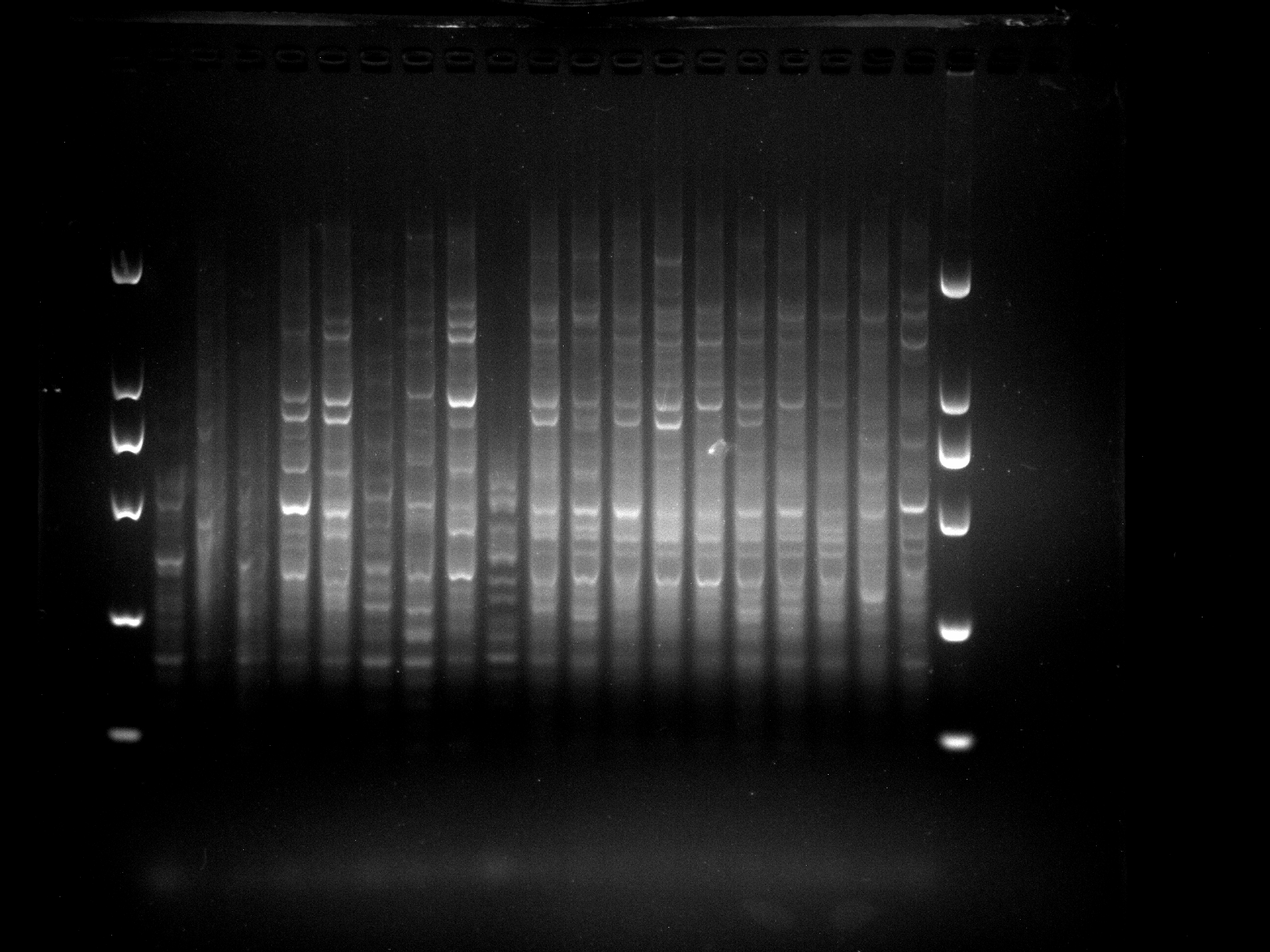

Supplement: Supplemental Information 12 — Amplification results of WRKY-F1 on NJD 33, LS 1-13, PTD1, LSD1. [file peerj-08-8498-s012.png]

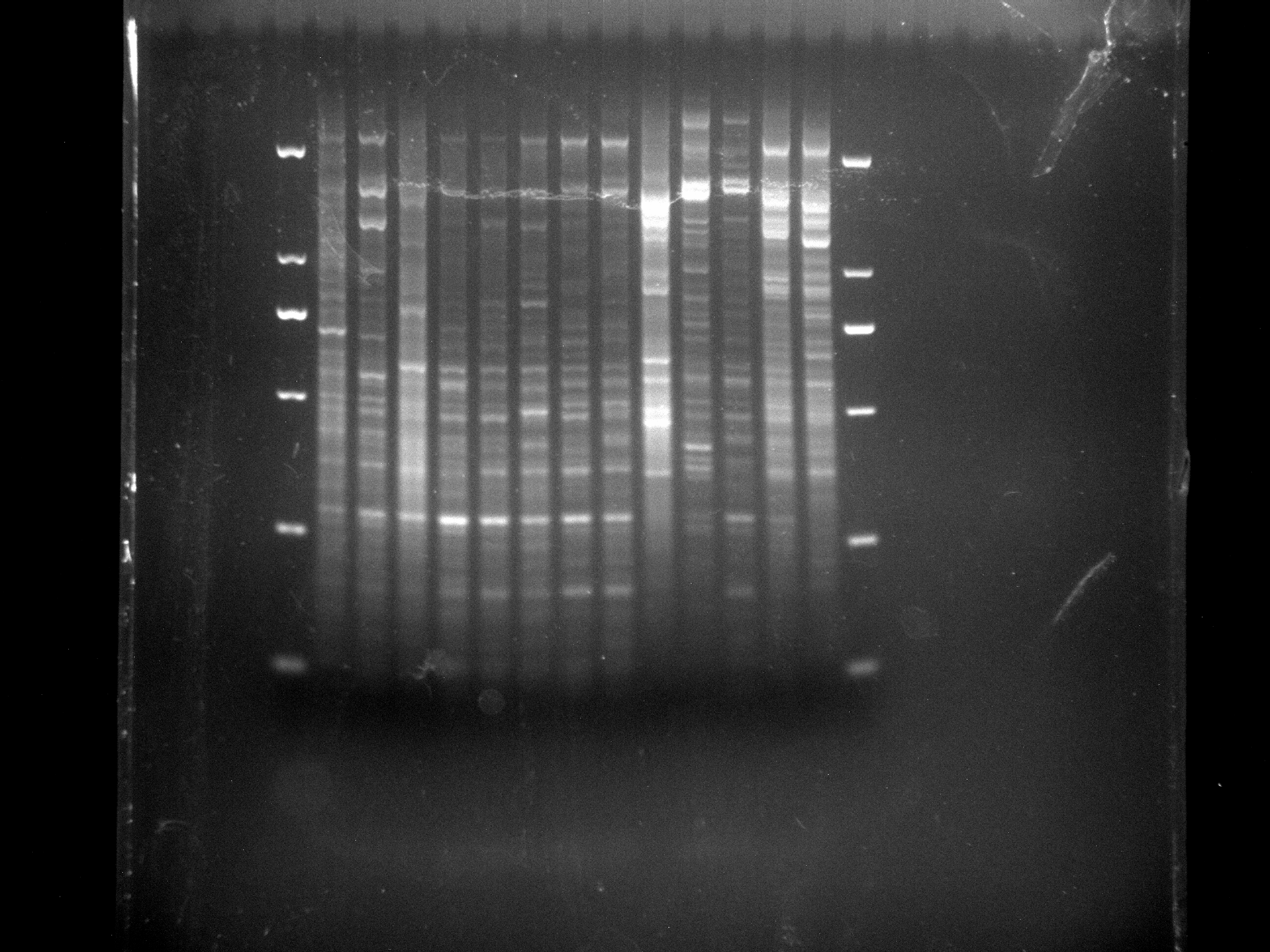

Supplement: Supplemental Information 13 — Amplification results of WRKY-F1 on DGD1-13. [file peerj-08-8498-s013.png]

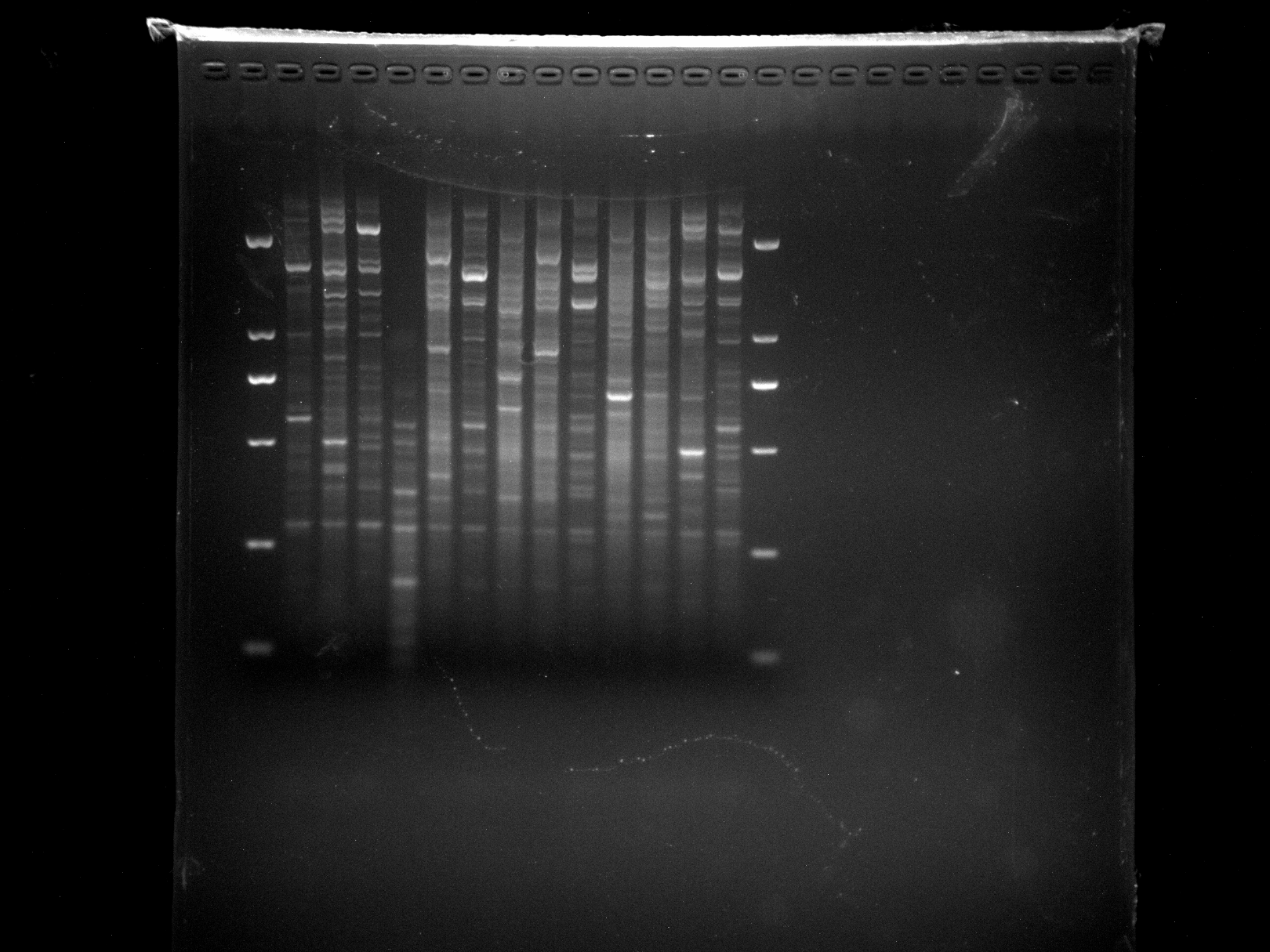

Supplement: Supplemental Information 14 — Amplification results of WRKY-F1 on DGD14-26. [file peerj-08-8498-s014.png]

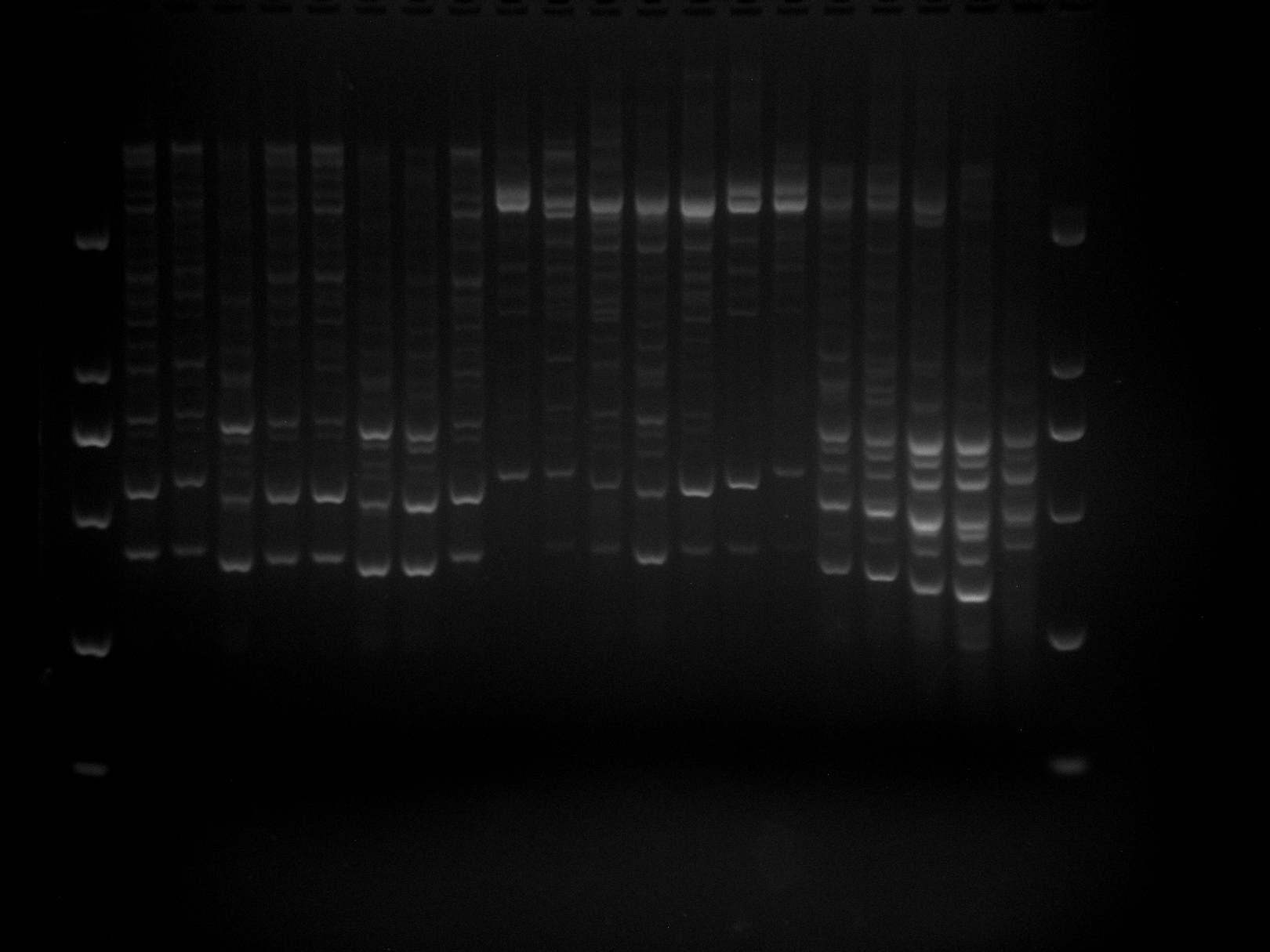

Supplement: Supplemental Information 15 — Amplification results of WRKY-R1 on LGD1-8 and DRS9-20. [file peerj-08-8498-s015.png]

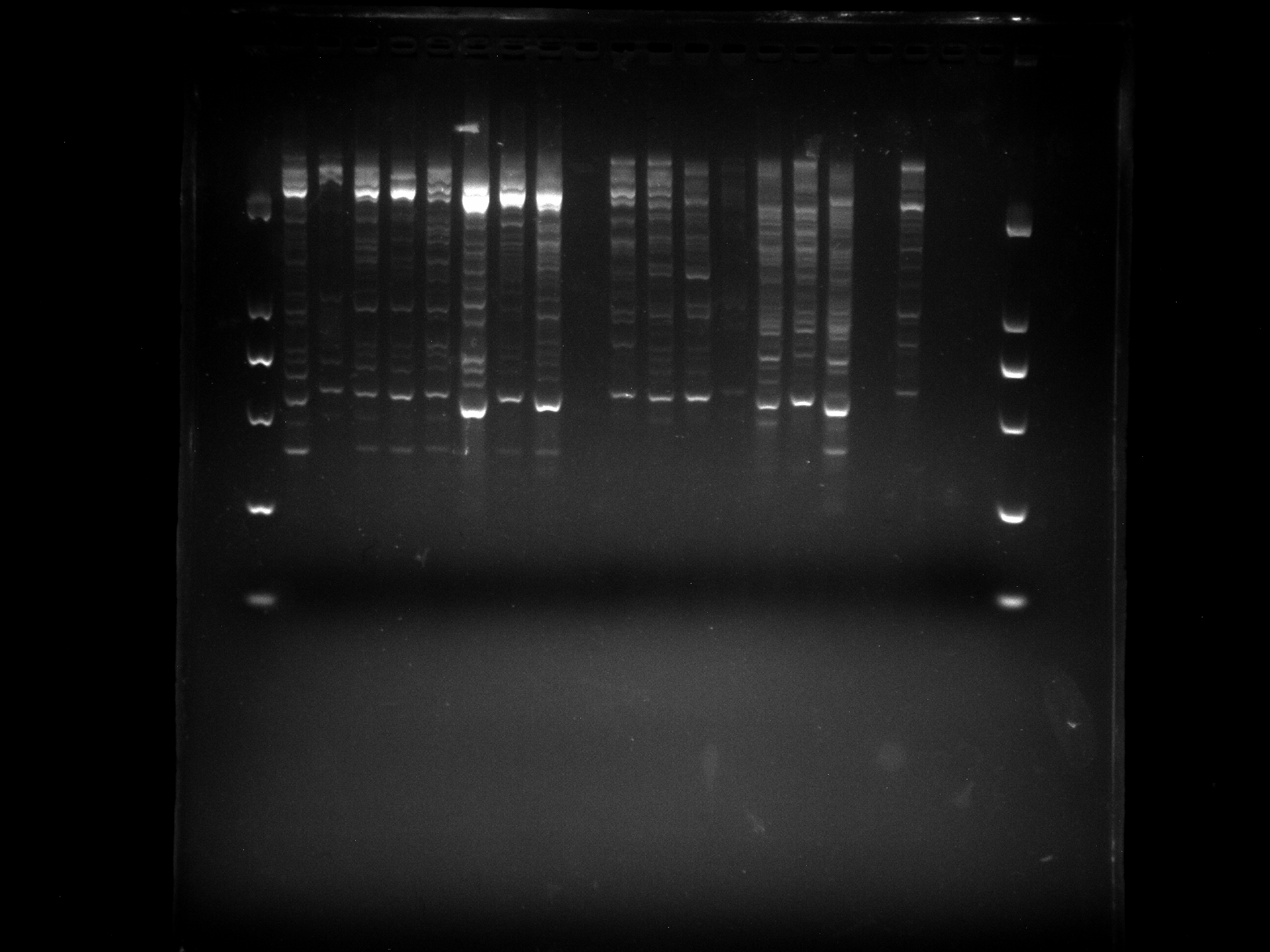

Supplement: Supplemental Information 16 — Amplification results of WRKY-R1 on DRS21-28, NJD1-12. [file peerj-08-8498-s016.png]

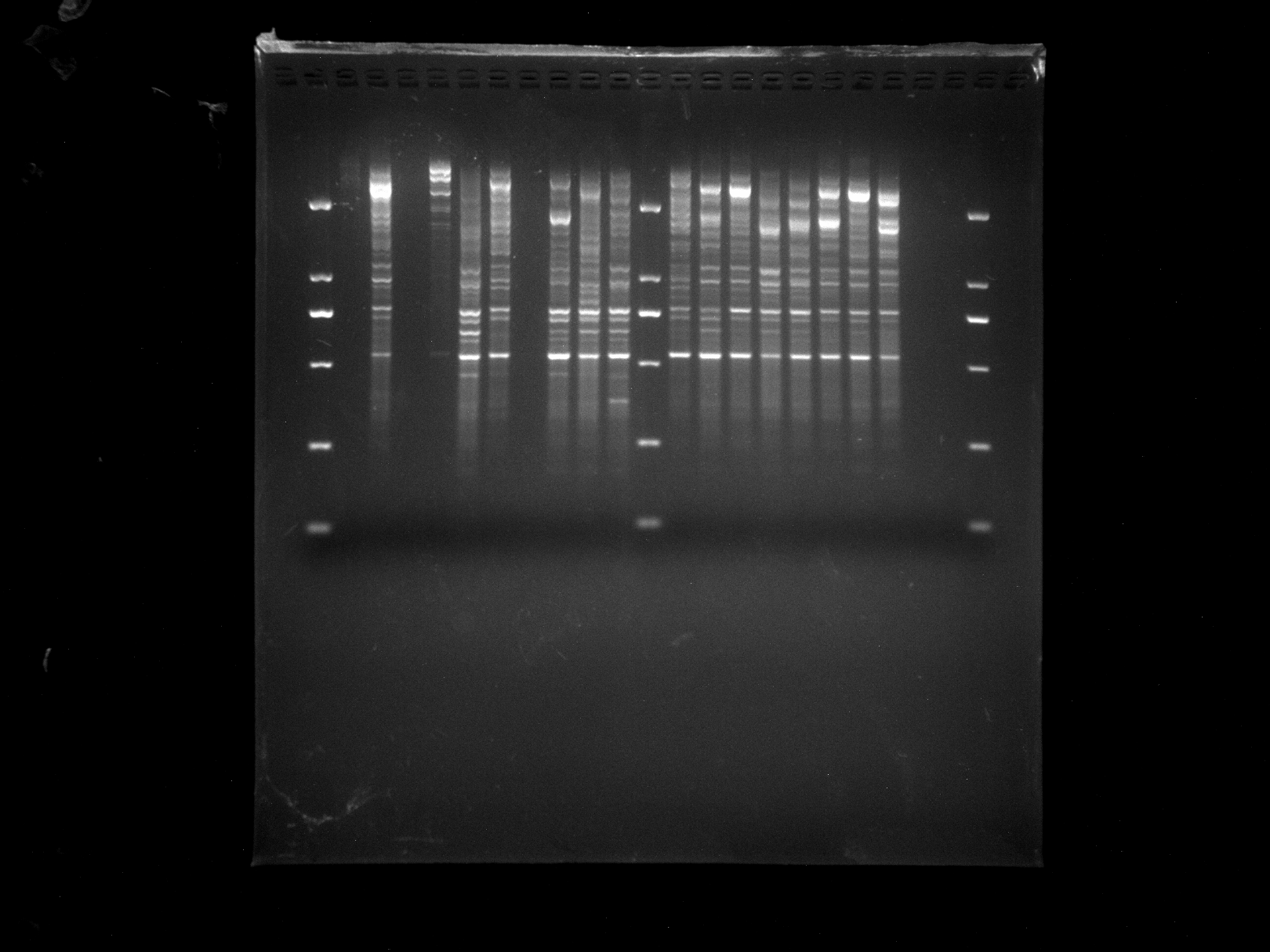

Supplement: Supplemental Information 17 — Amplification results of WRKY-R1 on NJD13-32. [file peerj-08-8498-s017.png]

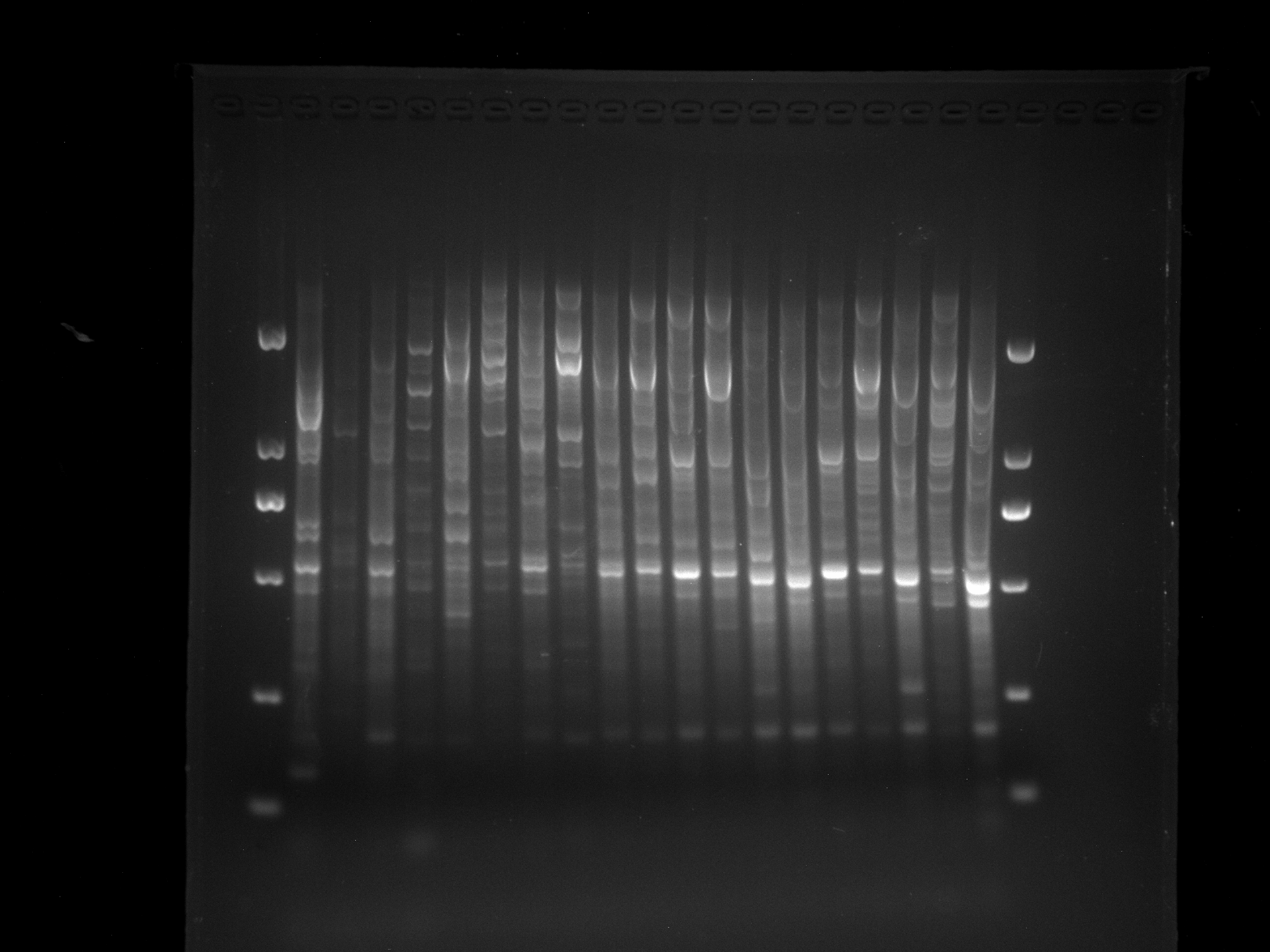

Supplement: Supplemental Information 18 — Amplification results of WRKY-R1 on NJD 33, LS1-13, PTD1, LSD1. [file peerj-08-8498-s018.png]

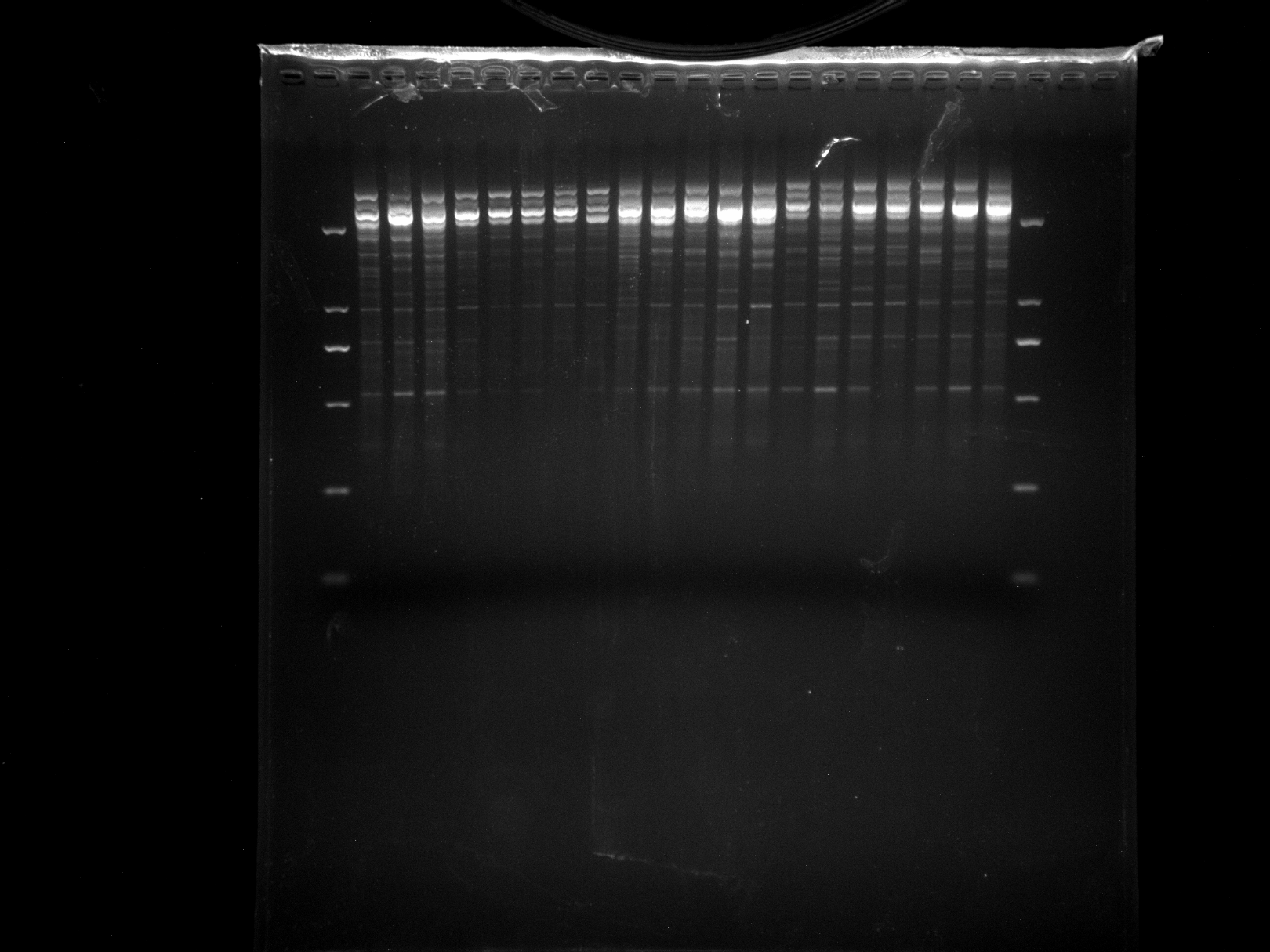

Supplement: Supplemental Information 19 — Amplification results of WRKY-R1 on DGD1-20. [file peerj-08-8498-s019.png]

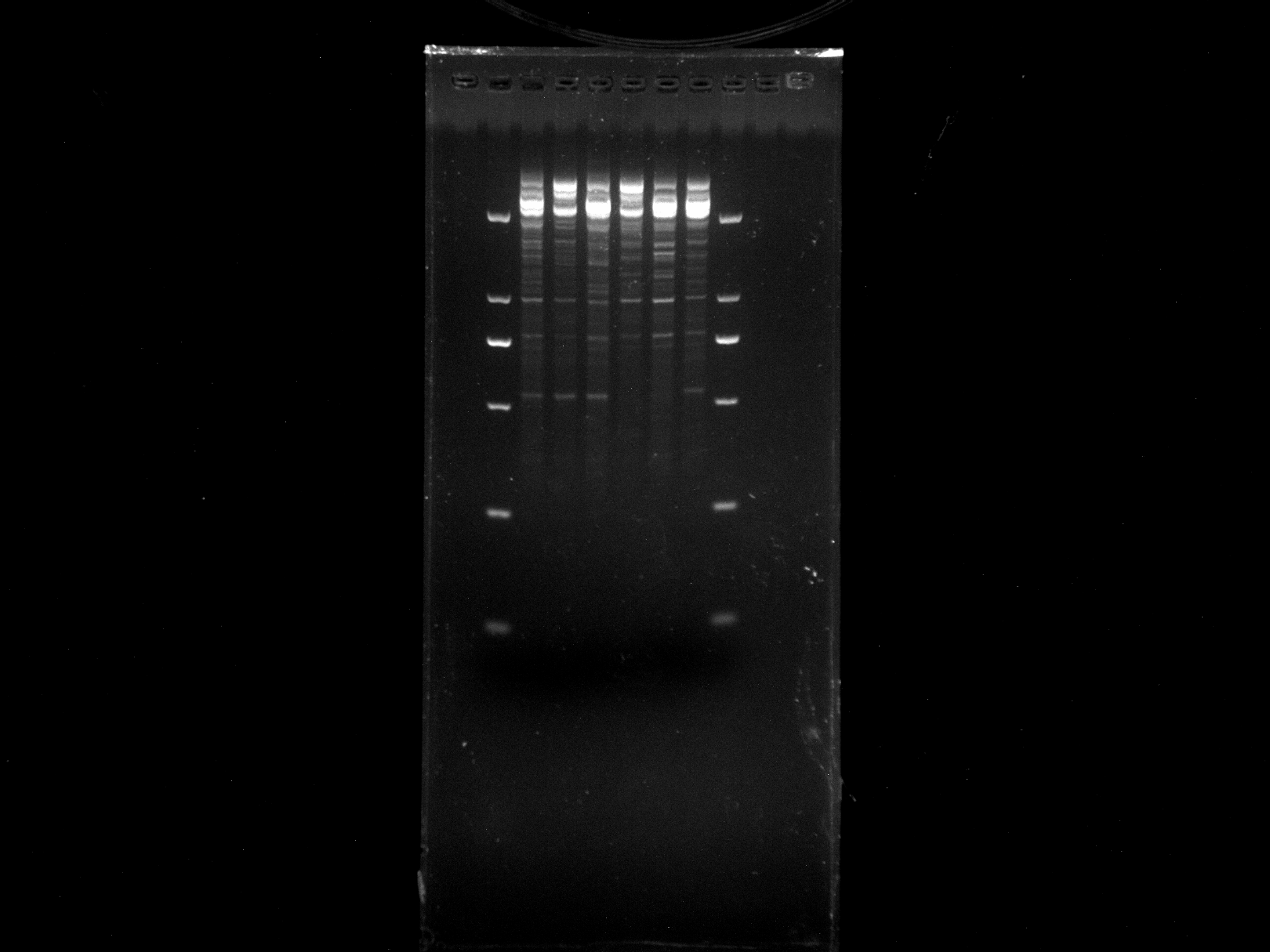

Supplement: Supplemental Information 20 — Amplification results of WRKY-R1 on DGD21-26. [file peerj-08-8498-s020.png]

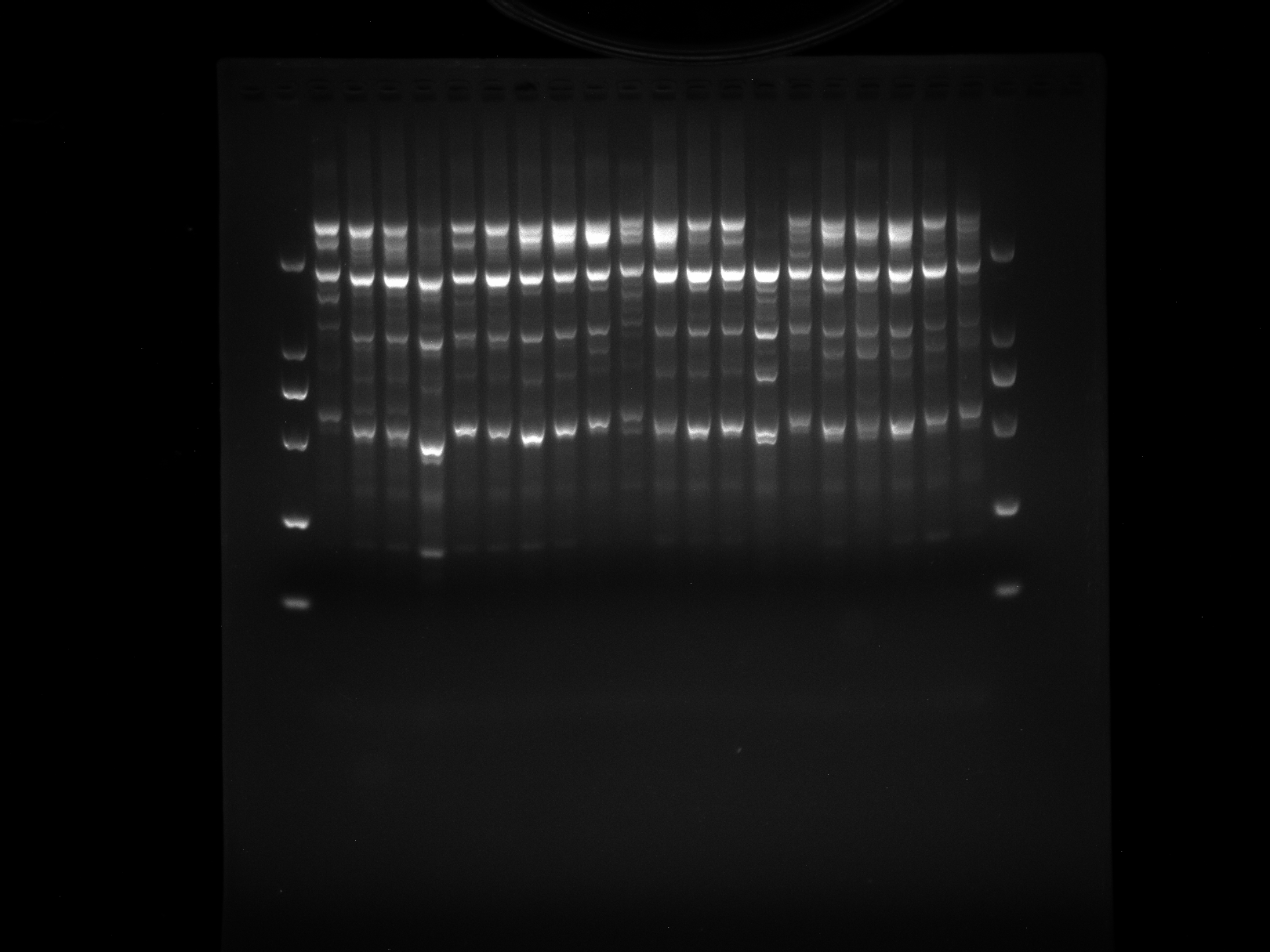

Supplement: Supplemental Information 21 — Amplification results of WRKY-R3 on LGD1-8, DRS9-20 samples. [file peerj-08-8498-s021.png]

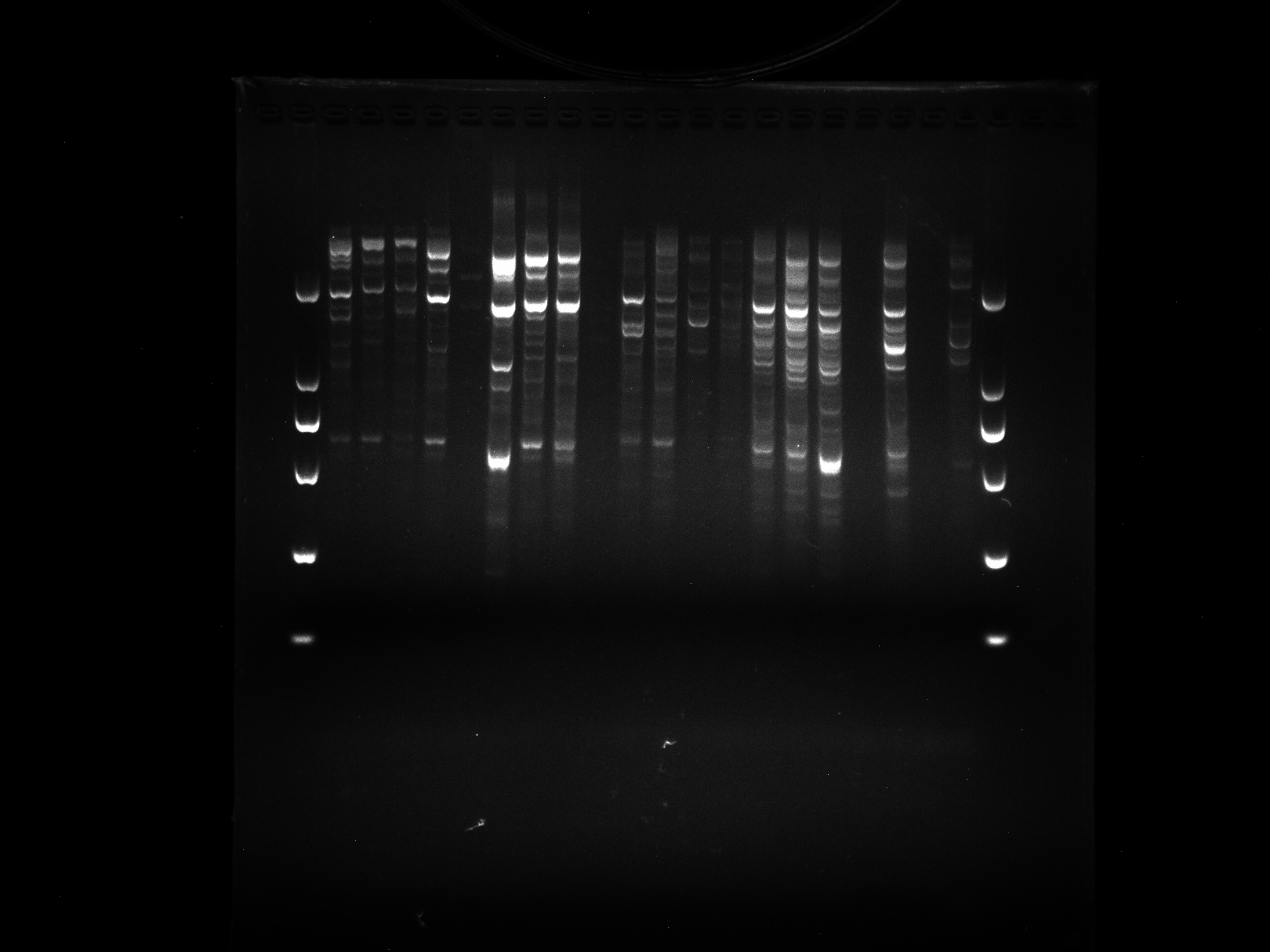

Supplement: Supplemental Information 22 — Amplification results of WRKY-R3 on DRS21-28, NJD1-12 samples. [file peerj-08-8498-s022.png]

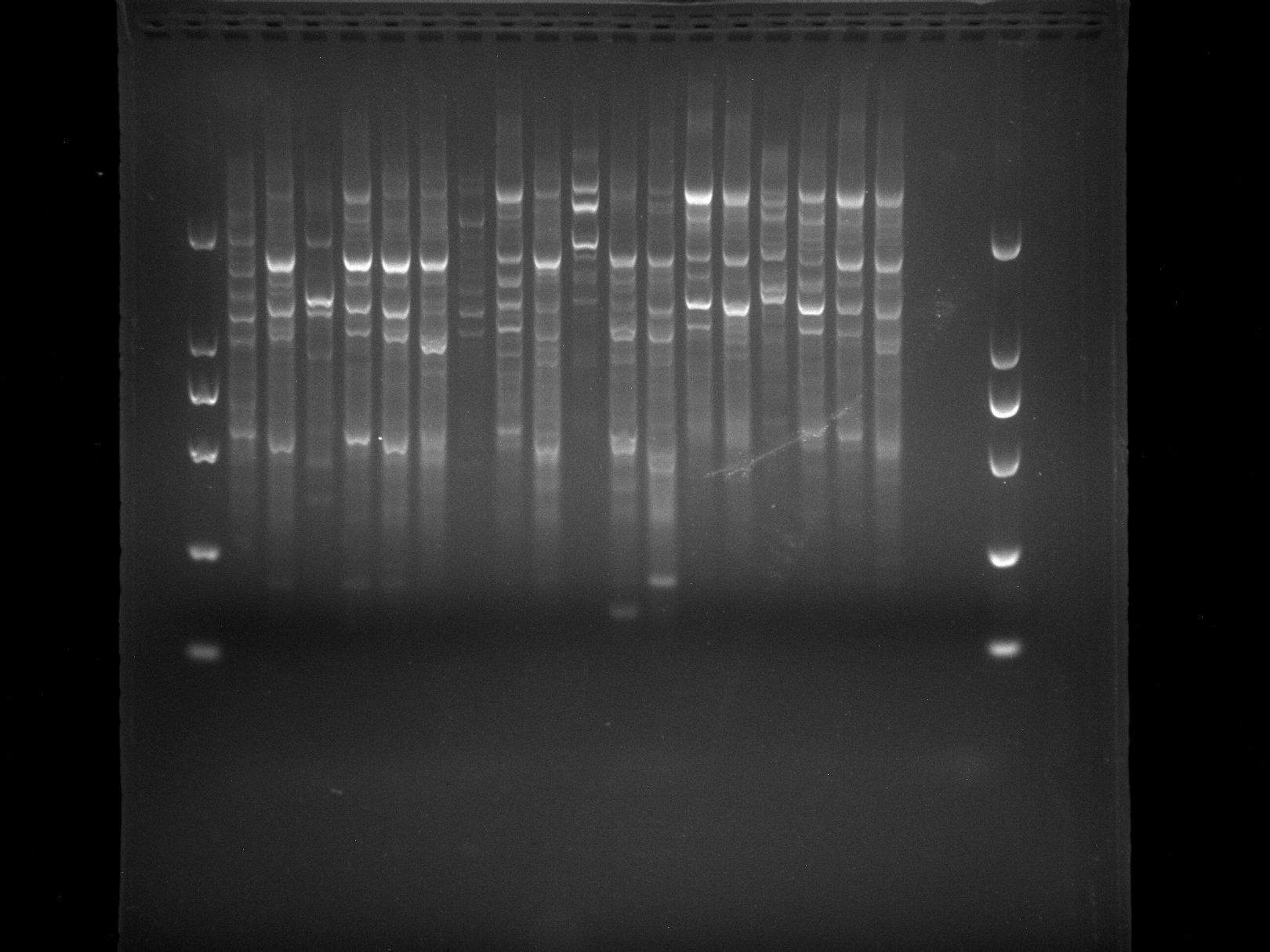

Supplement: Supplemental Information 23 — Amplification results of WRKY-R3 on DRS21-28, NJD13-32 samples. [file peerj-08-8498-s023.png]

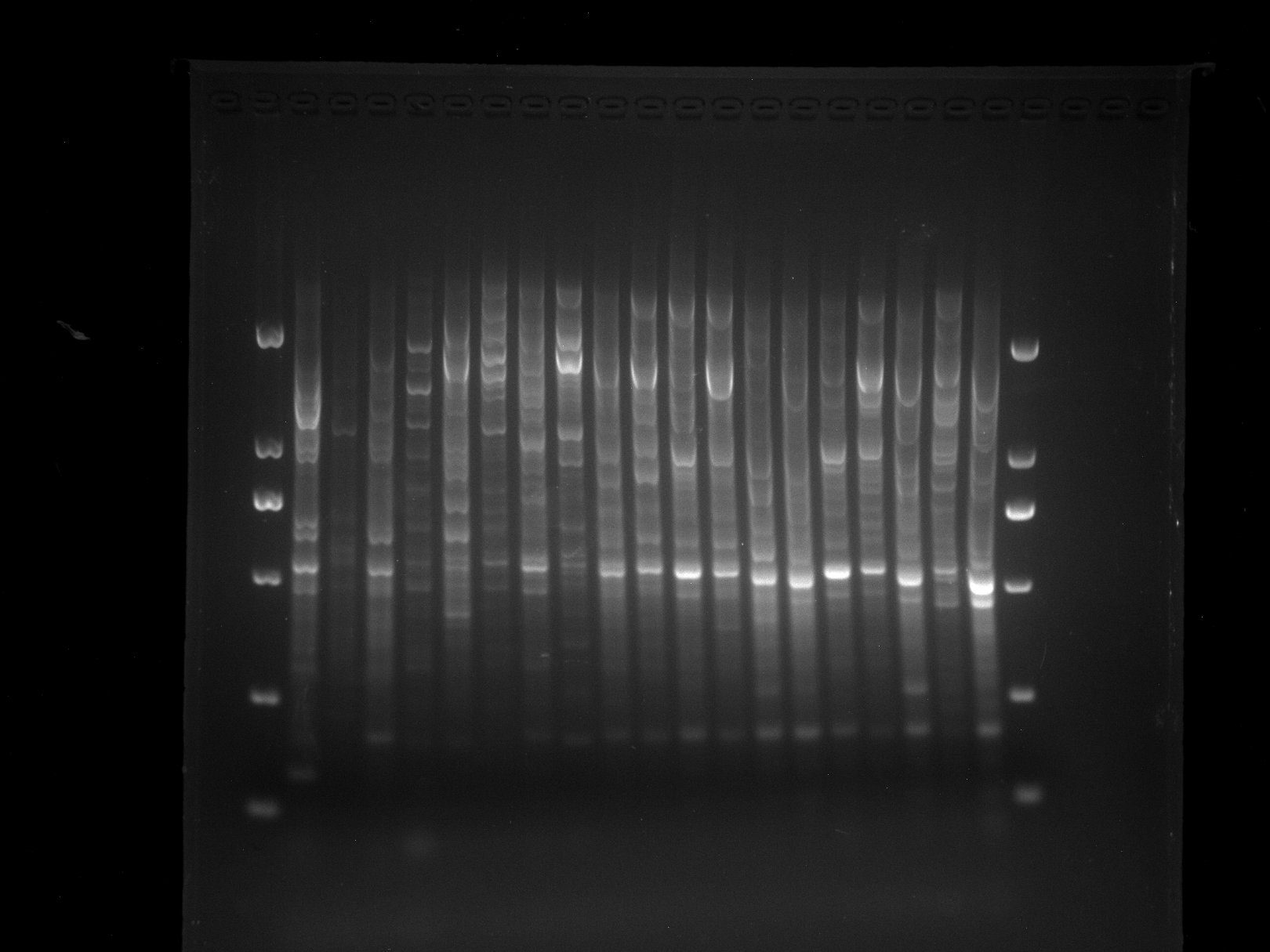

Supplement: Supplemental Information 24 — Amplification results of WRKY-R3 on NJD33, LS1-13, PTD1, LSD1 samples. [file peerj-08-8498-s024.png]

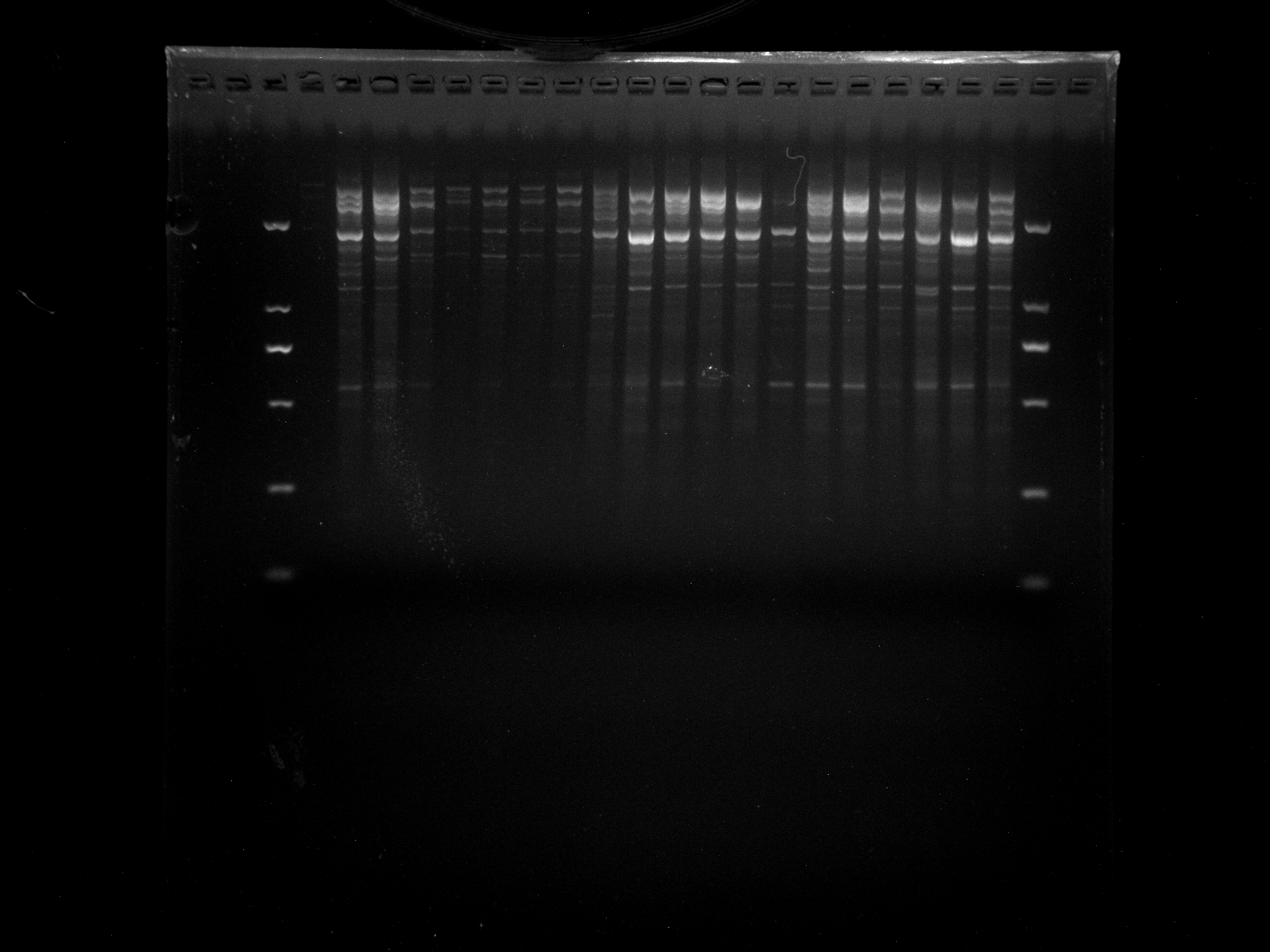

Supplement: Supplemental Information 25 — Amplification results of WRKY-R3 on DGD1-20 samples. [file peerj-08-8498-s025.png]

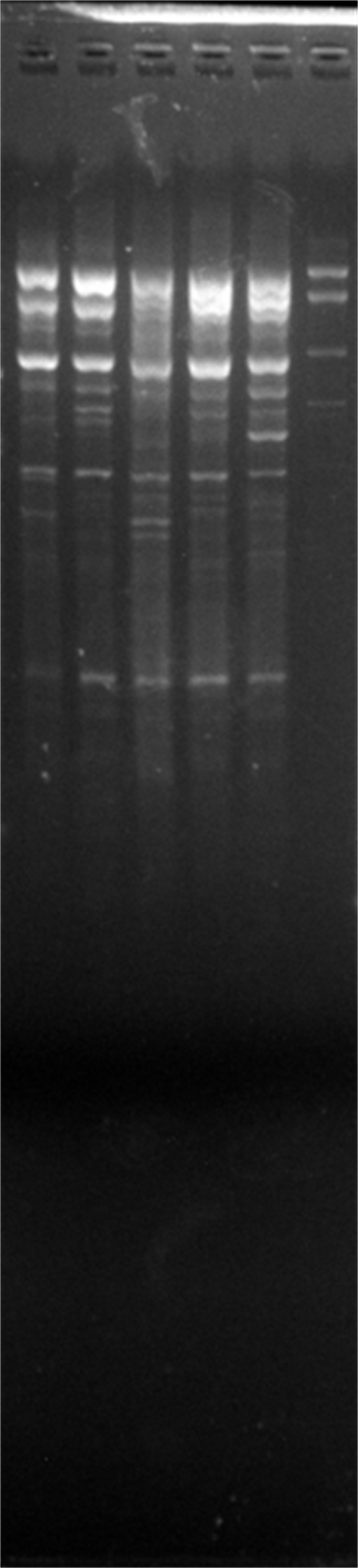

Supplement: Supplemental Information 26 — Amplification results of WRKY-R3 on DGD21-26 samples. [file peerj-08-8498-s026.png]

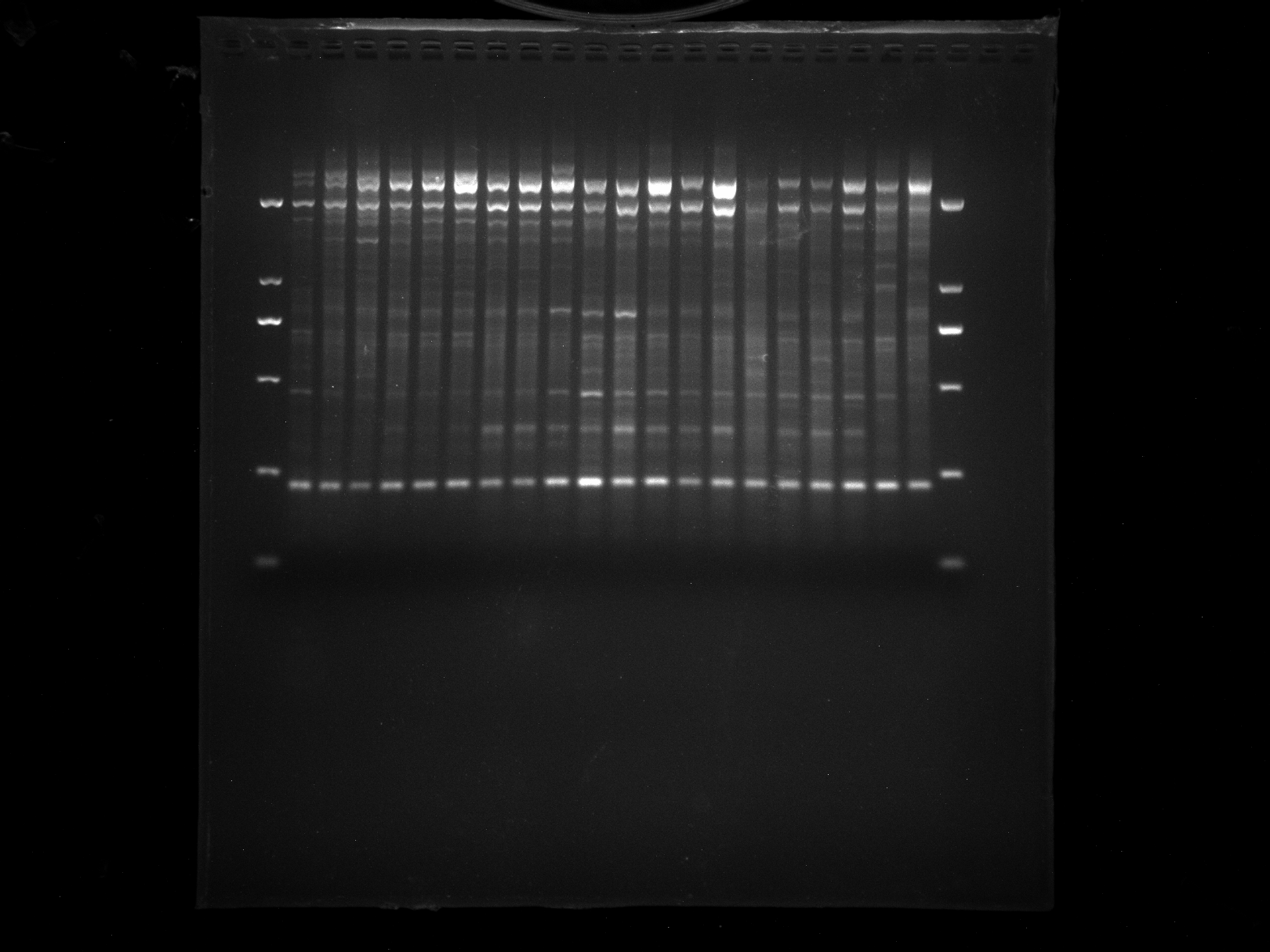

Supplement: Supplemental Information 27 — Amplification results of MYB1 on LGD1-8, DRS9-20 samples. [file peerj-08-8498-s027.png]

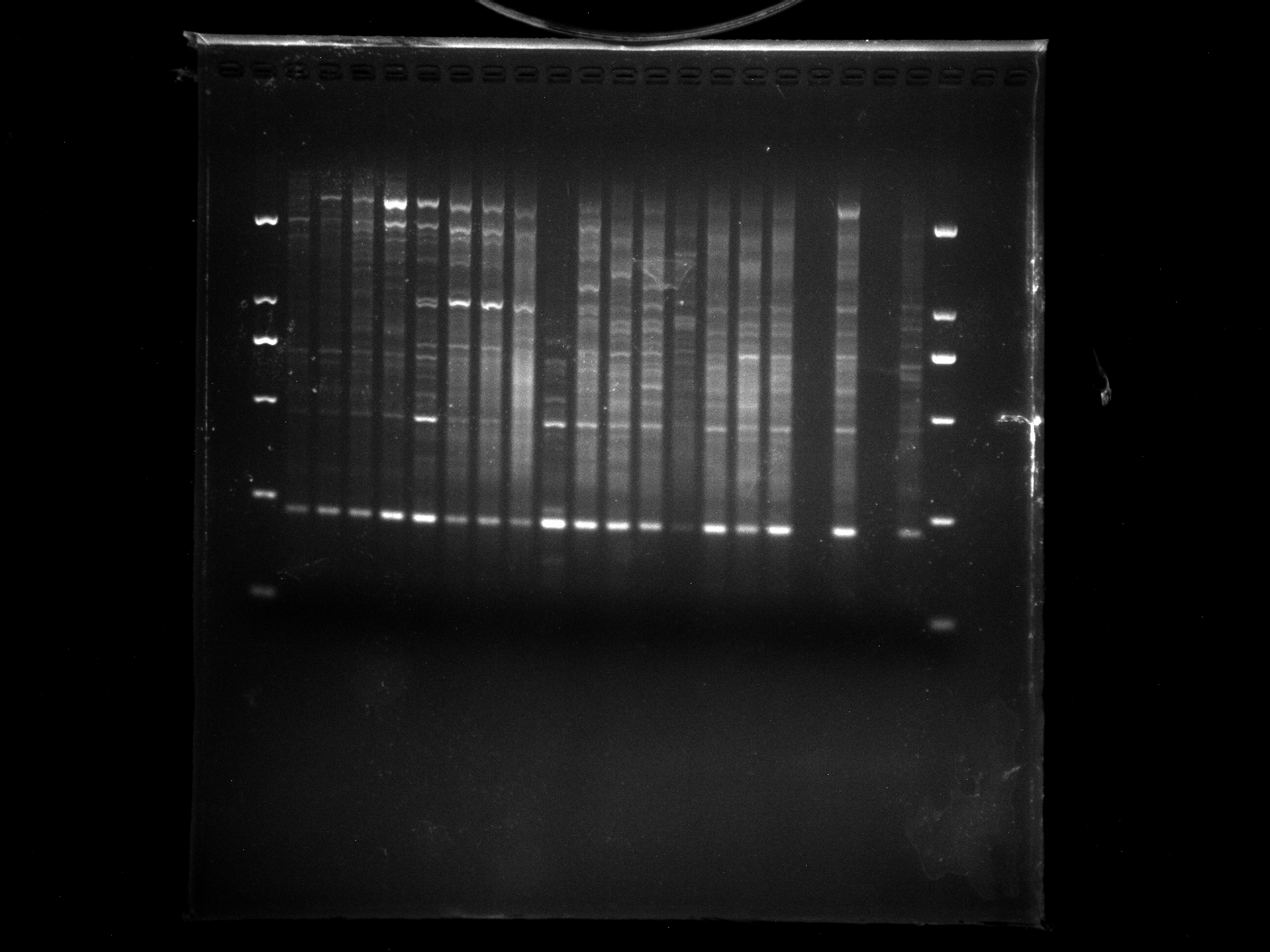

Supplement: Supplemental Information 28 — Amplification results of MYB1 on DRS21-28, NJD1-12 samples. [file peerj-08-8498-s028.png]

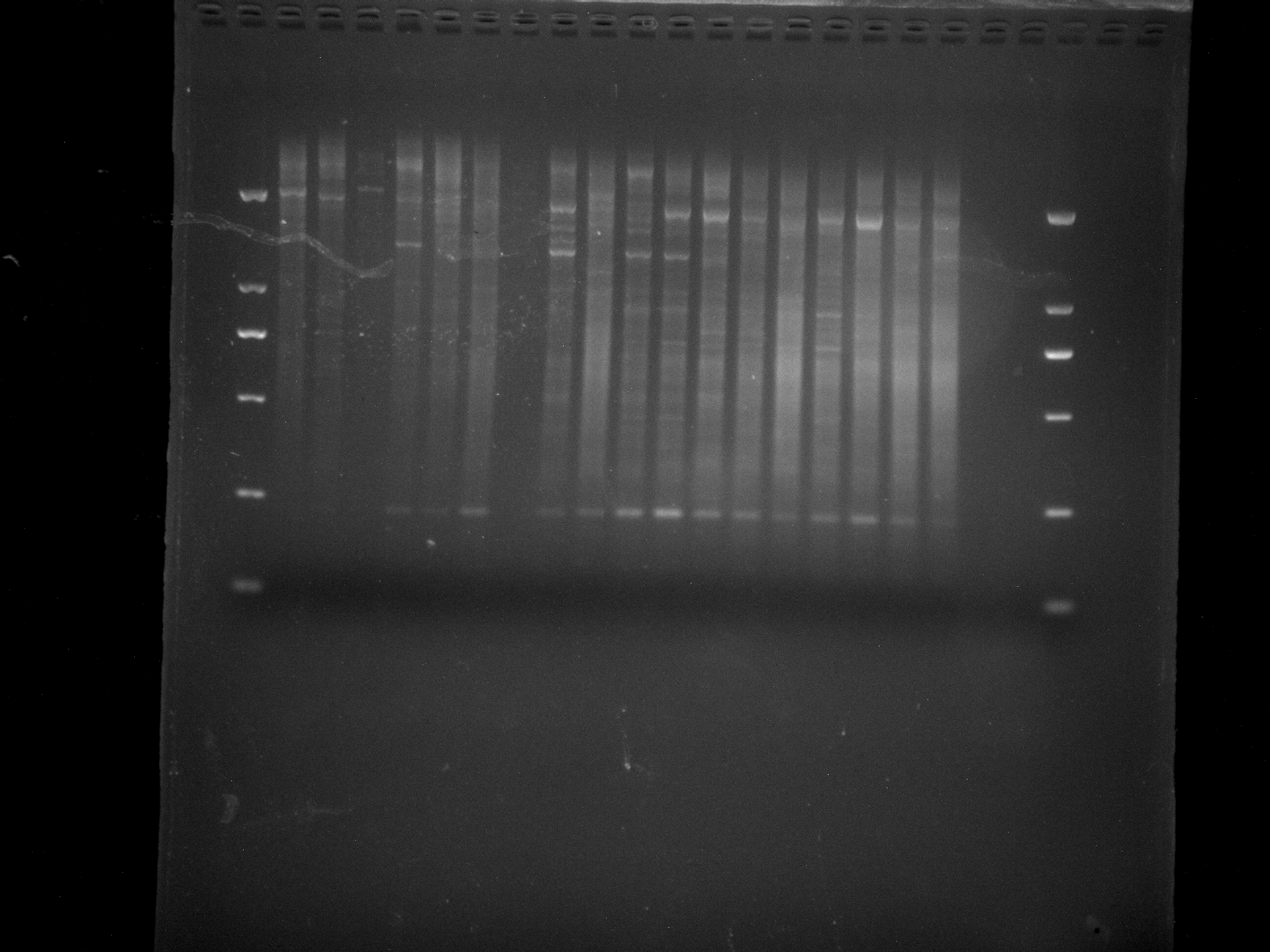

Supplement: Supplemental Information 29 — Amplification results of MYB1 on NJD13-33 samples. [file peerj-08-8498-s029.png]

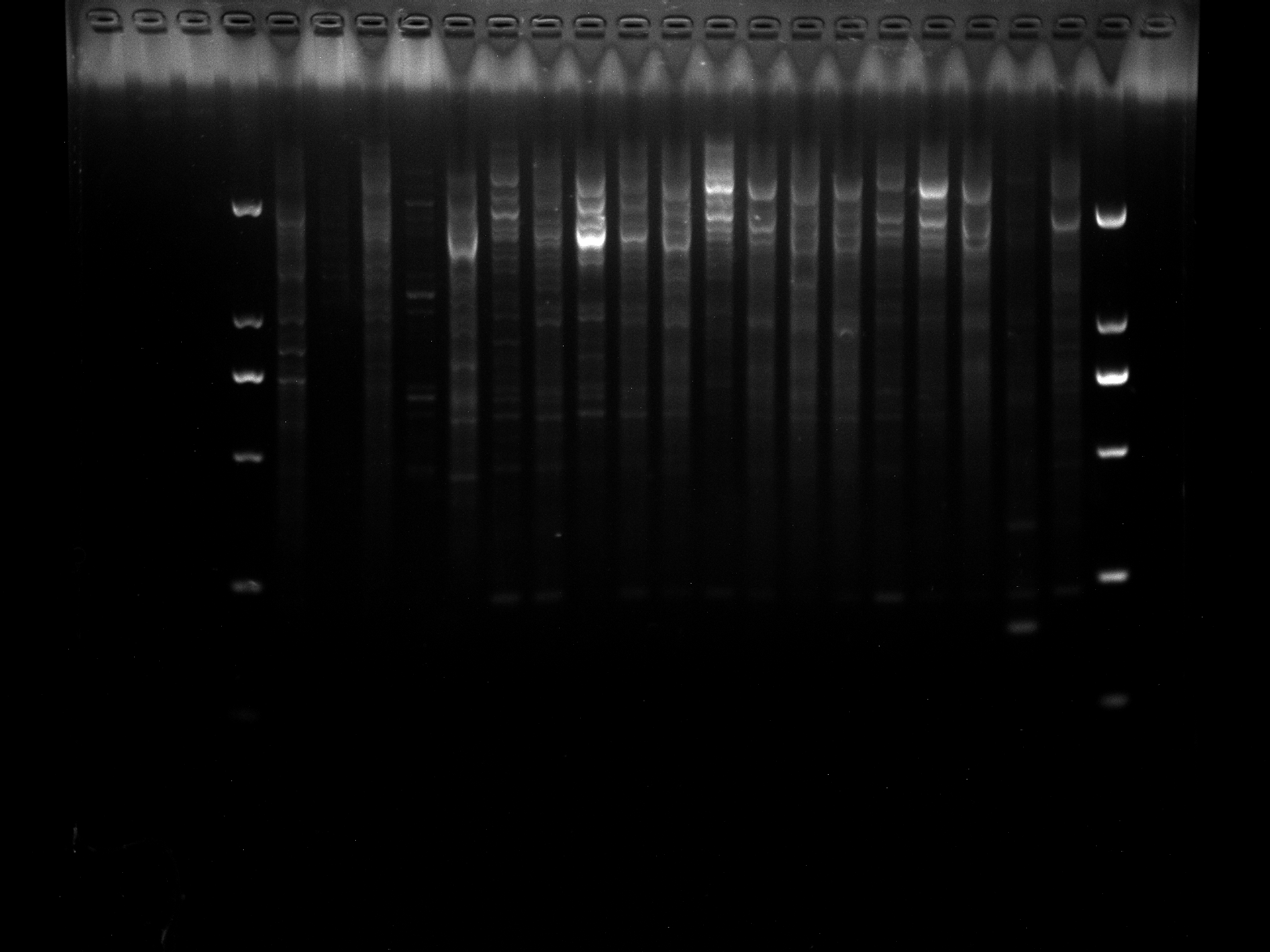

Supplement: Supplemental Information 30 — Amplification results of MYB1 on NJD33, LS1-13, PTD1, LSD1 samples. [file peerj-08-8498-s030.png]

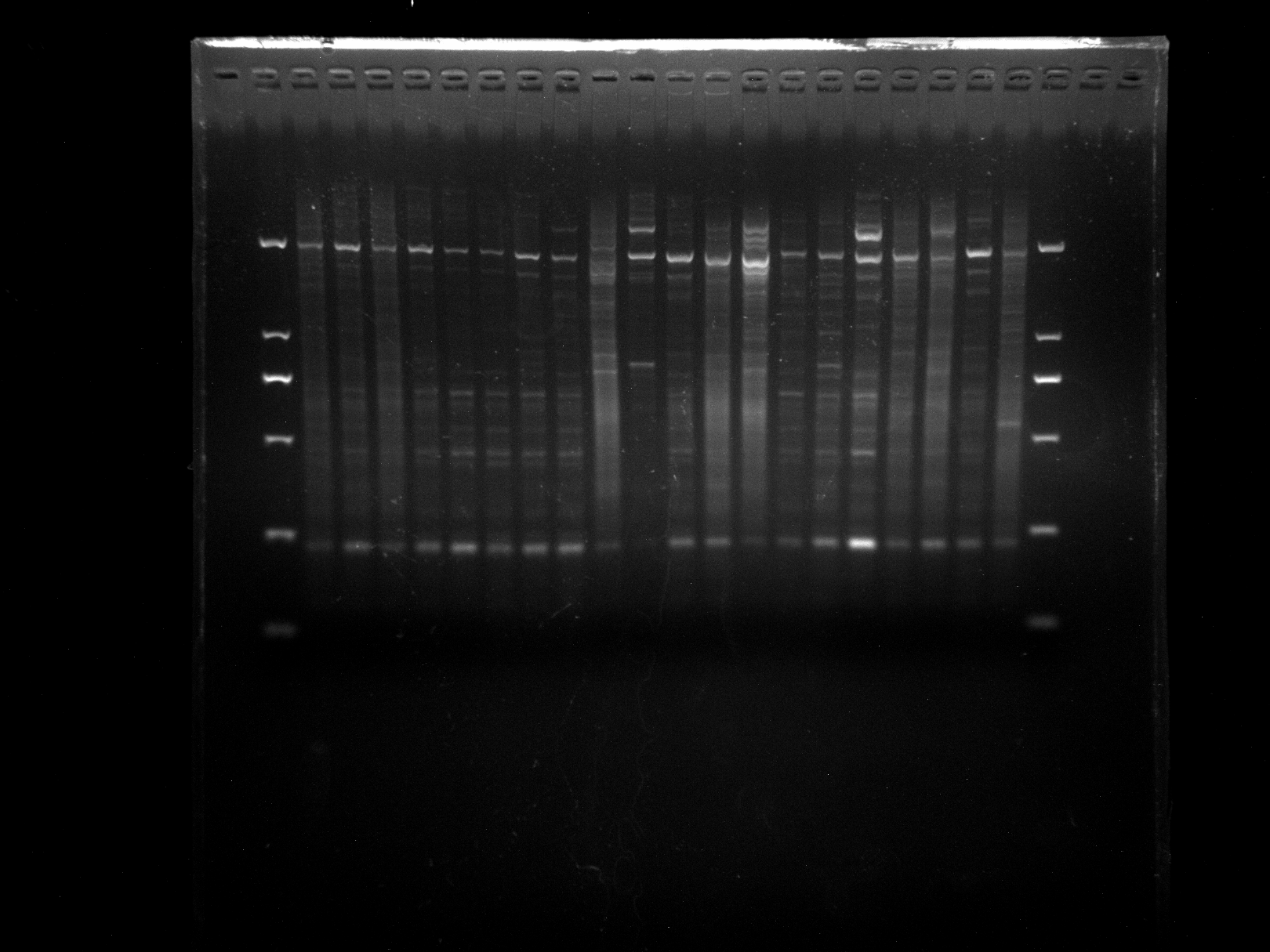

Supplement: Supplemental Information 31 — Amplification results of MYB1 on DGD1-20 samples. [file peerj-08-8498-s031.png]

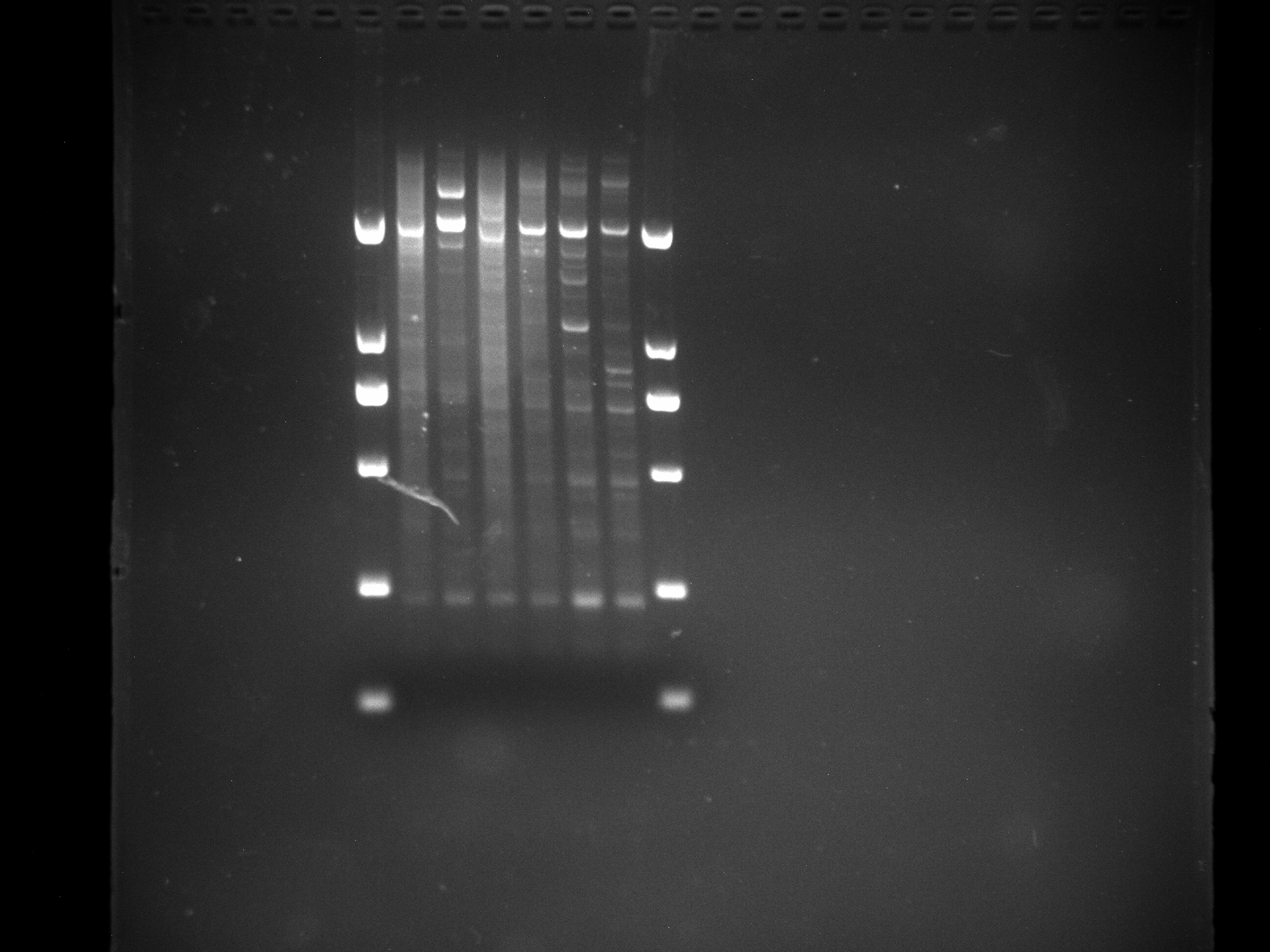

Supplement: Supplemental Information 32 — Amplification results of MYB1 on DGD21-26 samples. [file peerj-08-8498-s032.png]

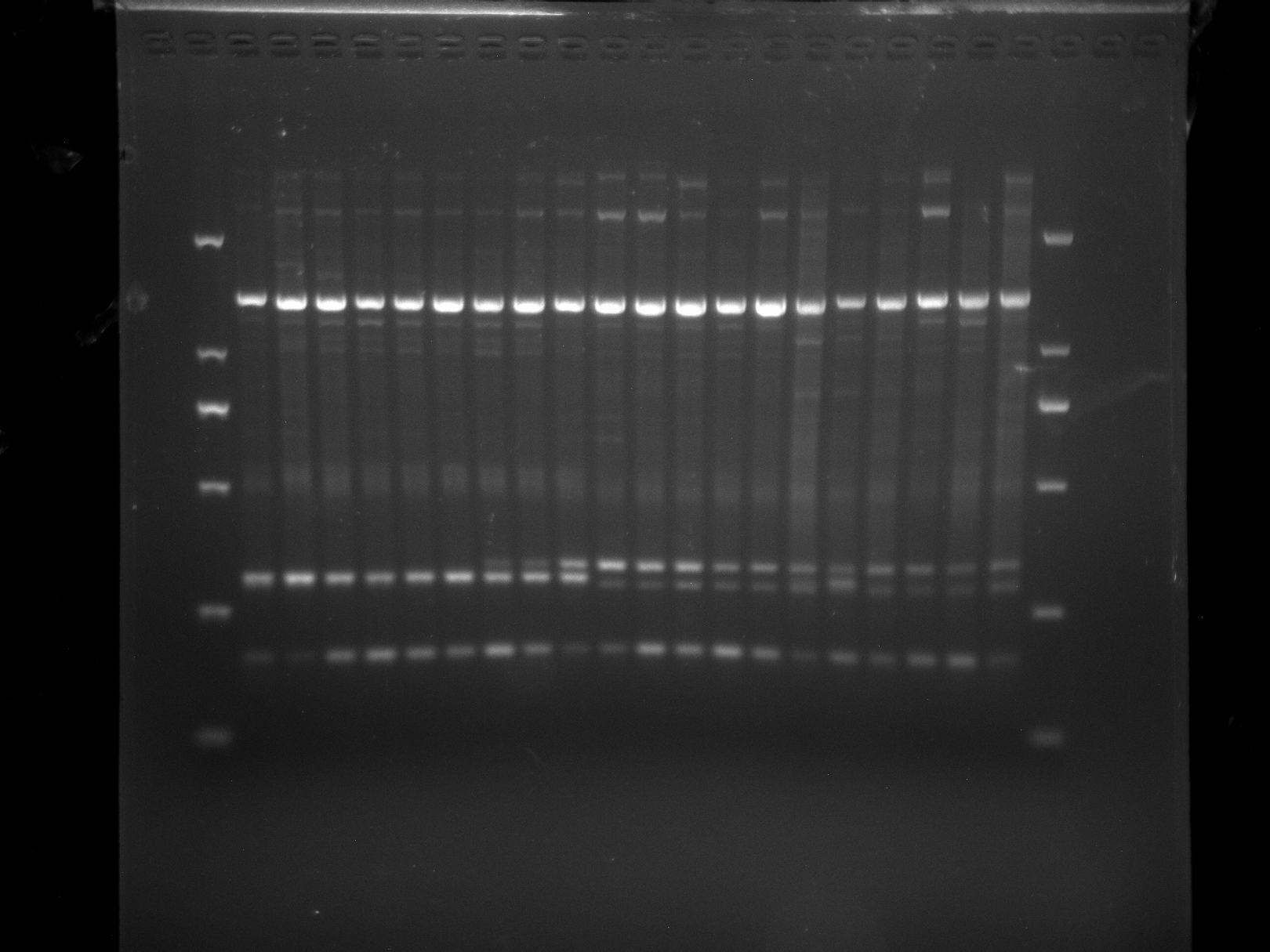

Supplement: Supplemental Information 33 — Amplification results of MYB2 on LGD1-8, DRS9-20 samples. [file peerj-08-8498-s033.png]

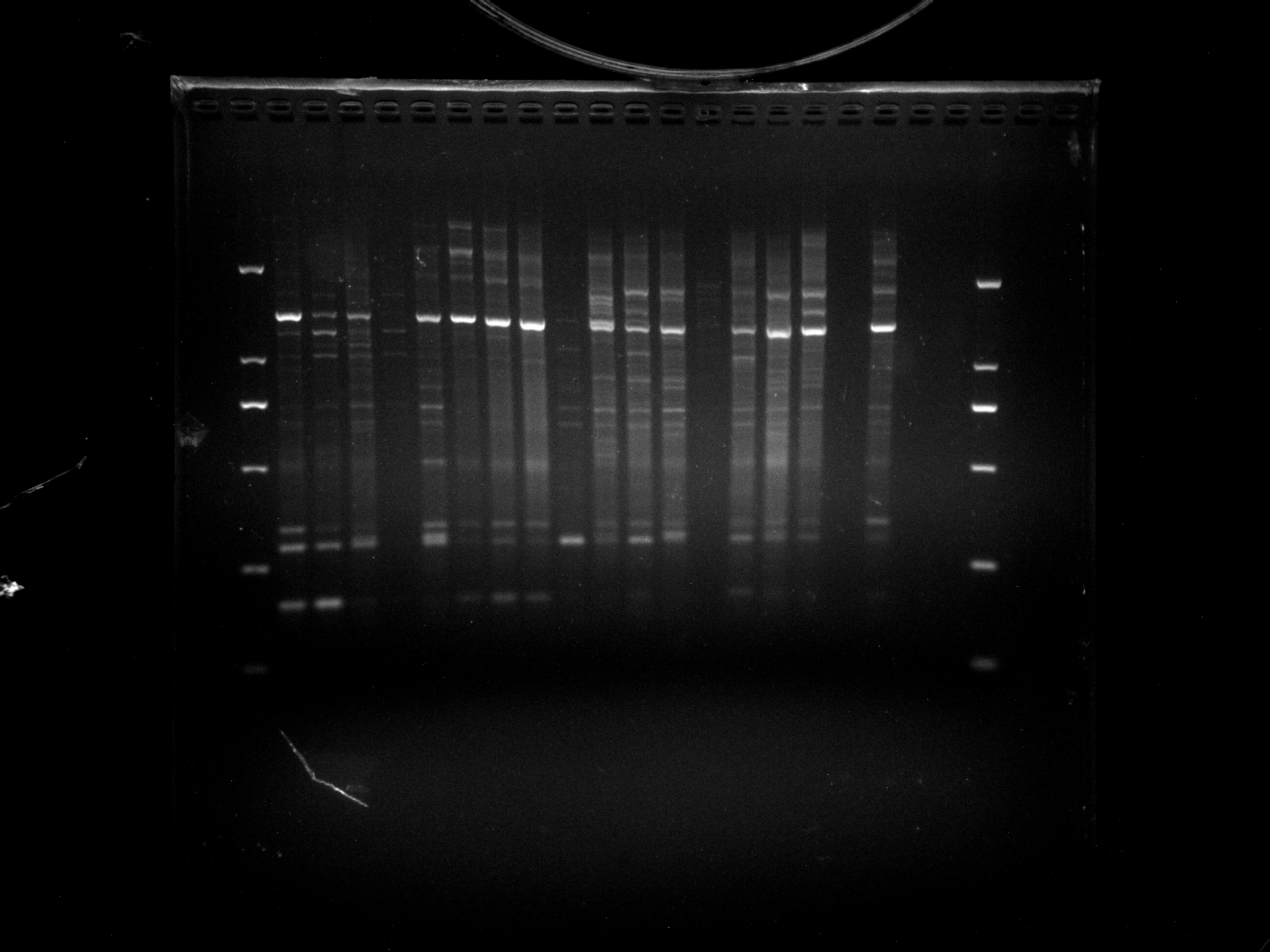

Supplement: Supplemental Information 34 — Amplification results of MYB2 on DRS21-28, NJD1-12 samples. [file peerj-08-8498-s034.png]

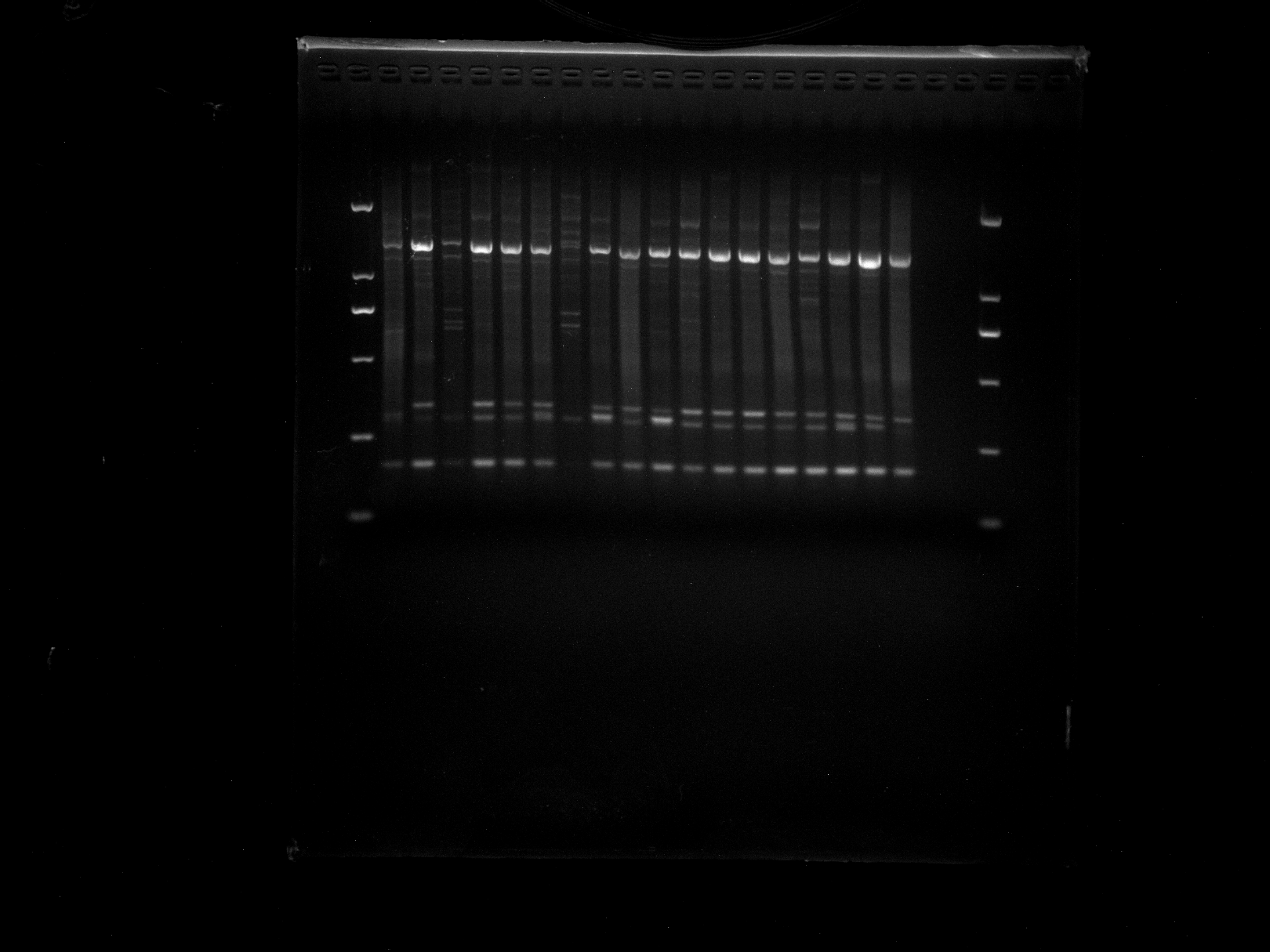

Supplement: Supplemental Information 35 — Amplification results of MYB2 on NJD13-32 samples. [file peerj-08-8498-s035.png]

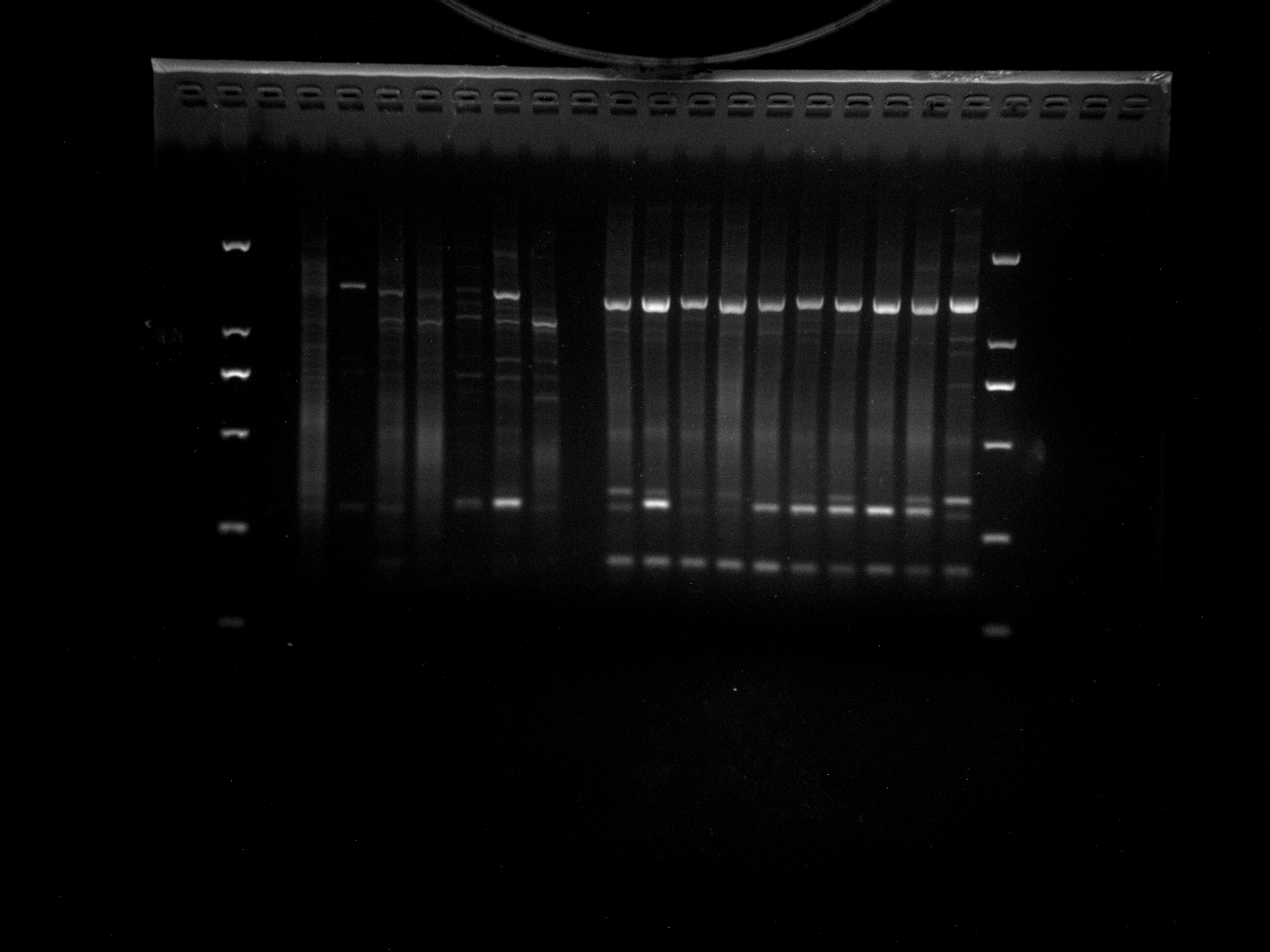

Supplement: Supplemental Information 36 — Amplification results of MYB2 on NJD33, LS1-13, PUD1, LSD1 samples. [file peerj-08-8498-s036.png]

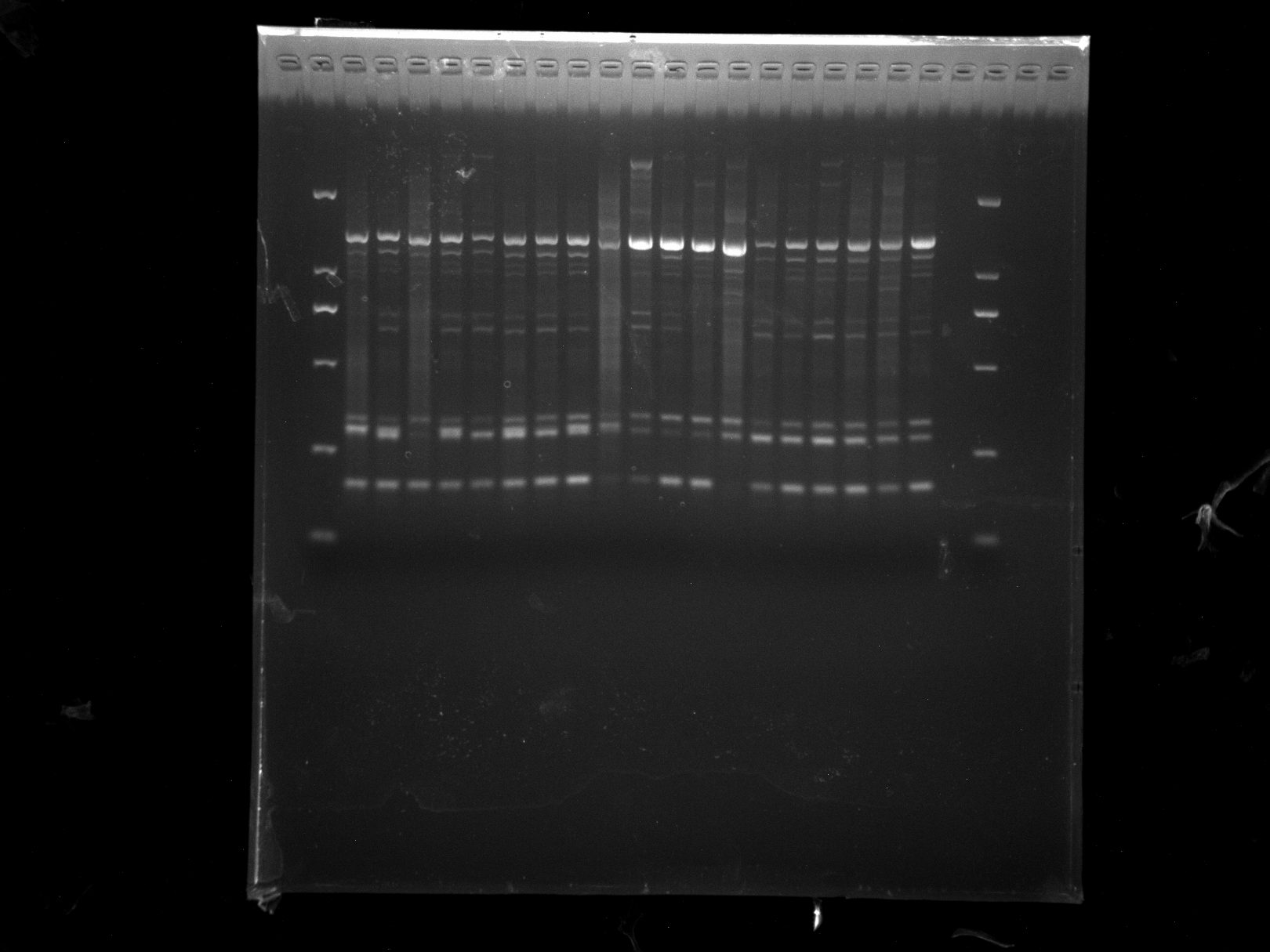

Supplement: Supplemental Information 37 — Amplification results of MYB2 on DGD1-20 samples. [file peerj-08-8498-s037.png]

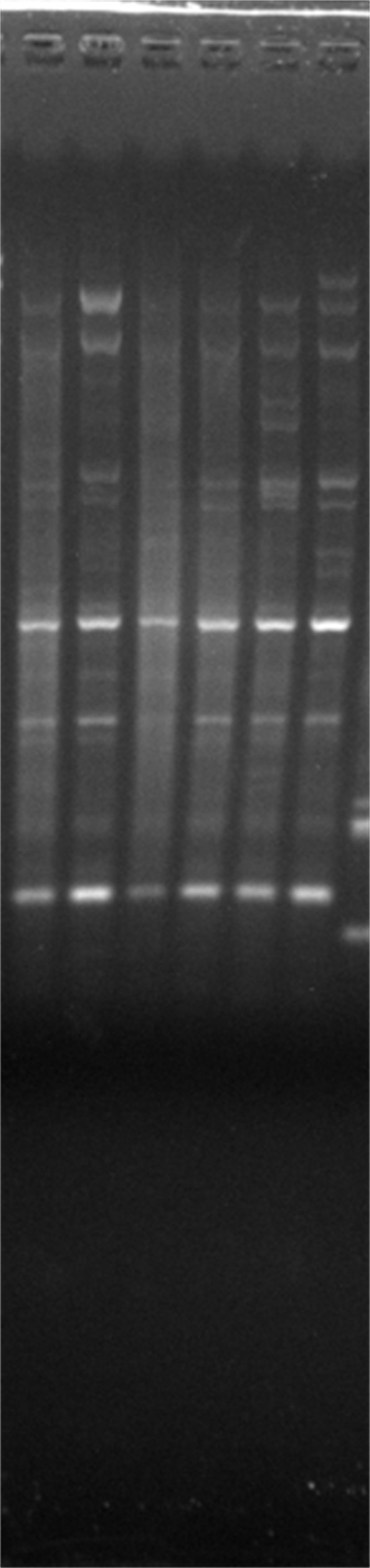

Supplement: Supplemental Information 38 — Amplification results of MYB2 on DGD21-26 samples. [file peerj-08-8498-s038.png]

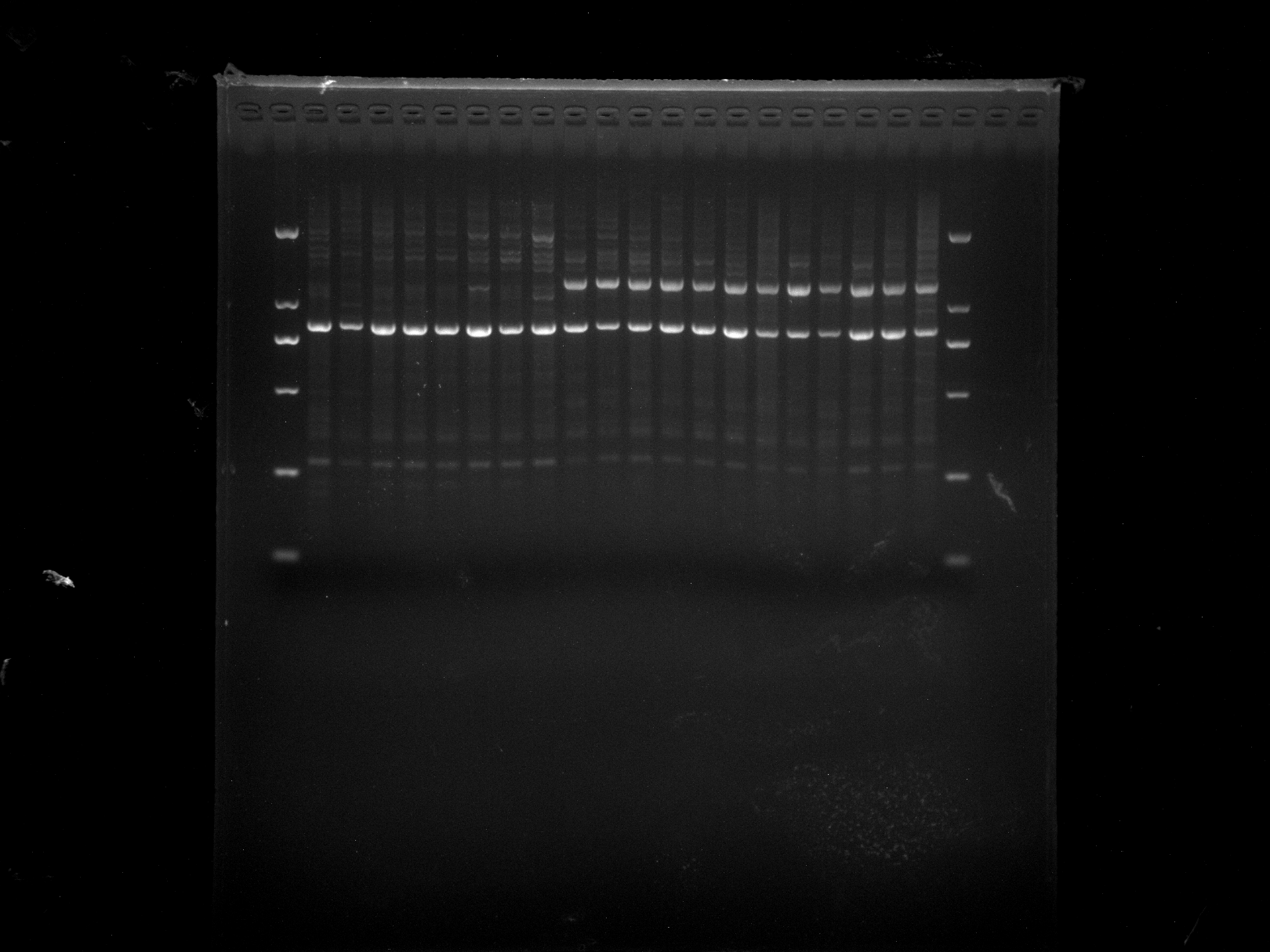

Supplement: Supplemental Information 39 — Amplification results of ERF1 on LGD1-8, DRS9-20 samples. [file peerj-08-8498-s039.png]

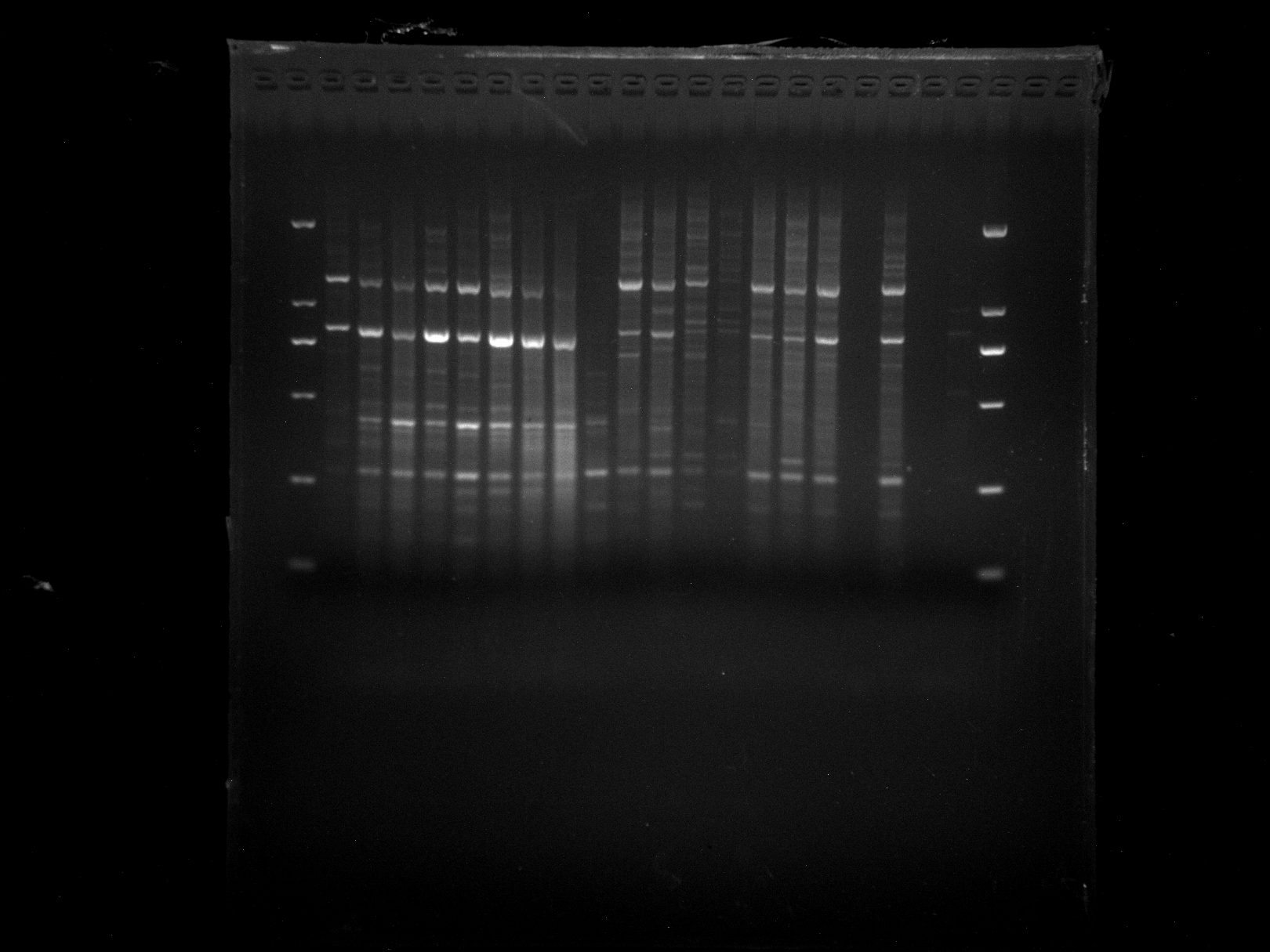

Supplement: Supplemental Information 40 — Amplification results of ERF1 on DRS21-28, NJD1-12 samples. [file peerj-08-8498-s040.png]

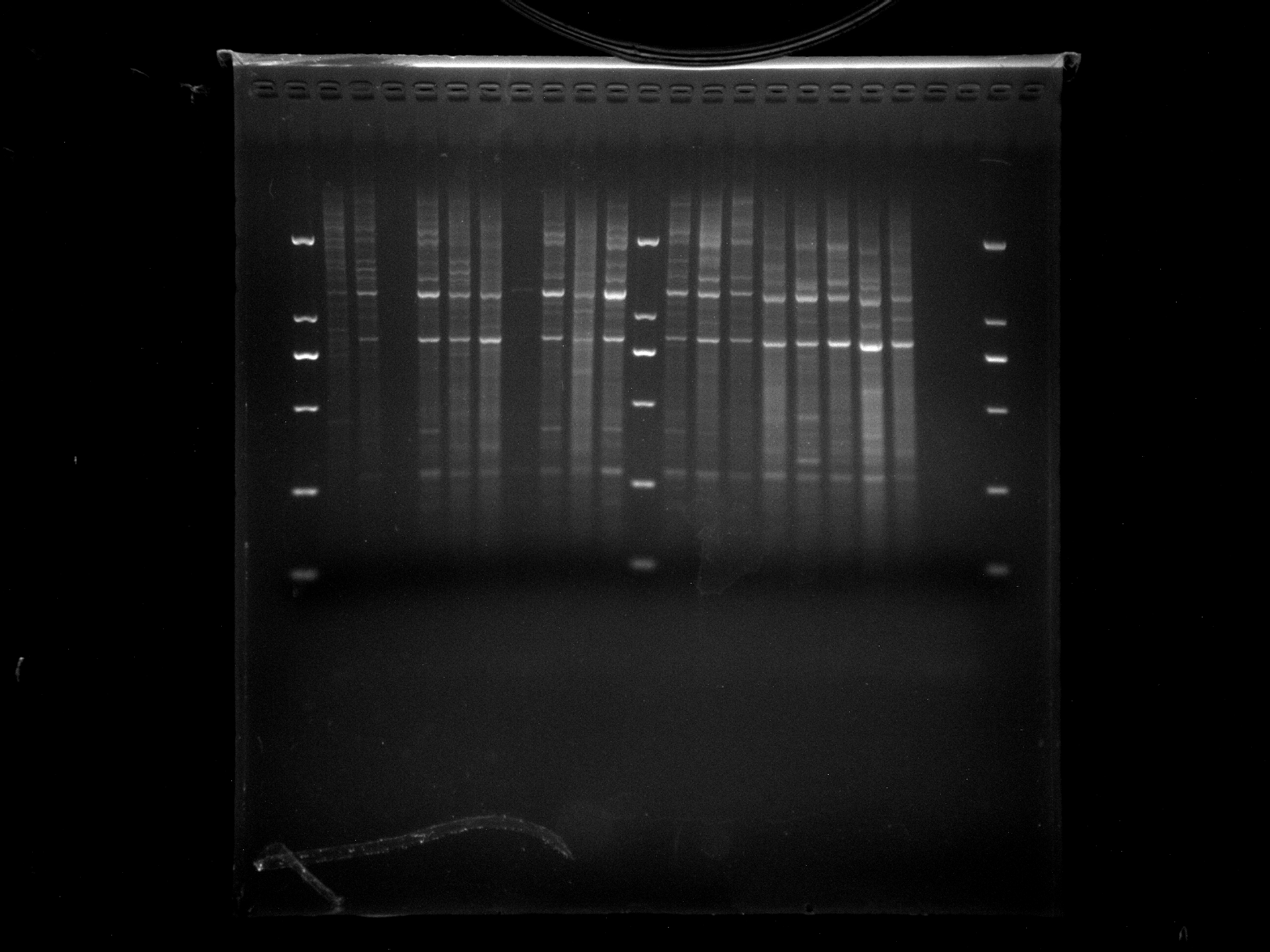

Supplement: Supplemental Information 41 — Amplification results of ERF1 on NJD13-32 samples. [file peerj-08-8498-s041.png]

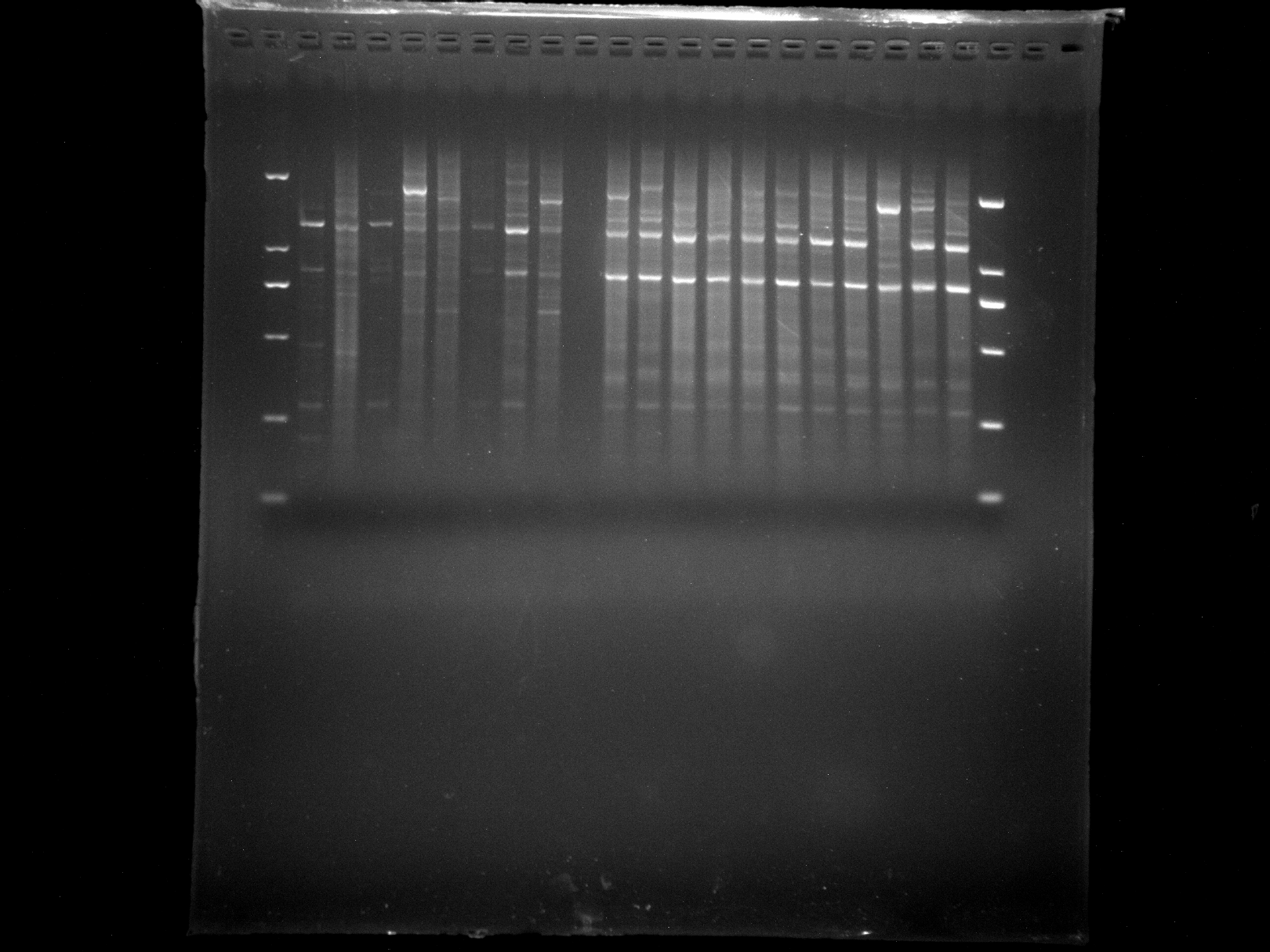

Supplement: Supplemental Information 42 — Amplification results of ERF1 on NJD33, LS1-13, PTD1, LSD1 samples. [file peerj-08-8498-s042.png]

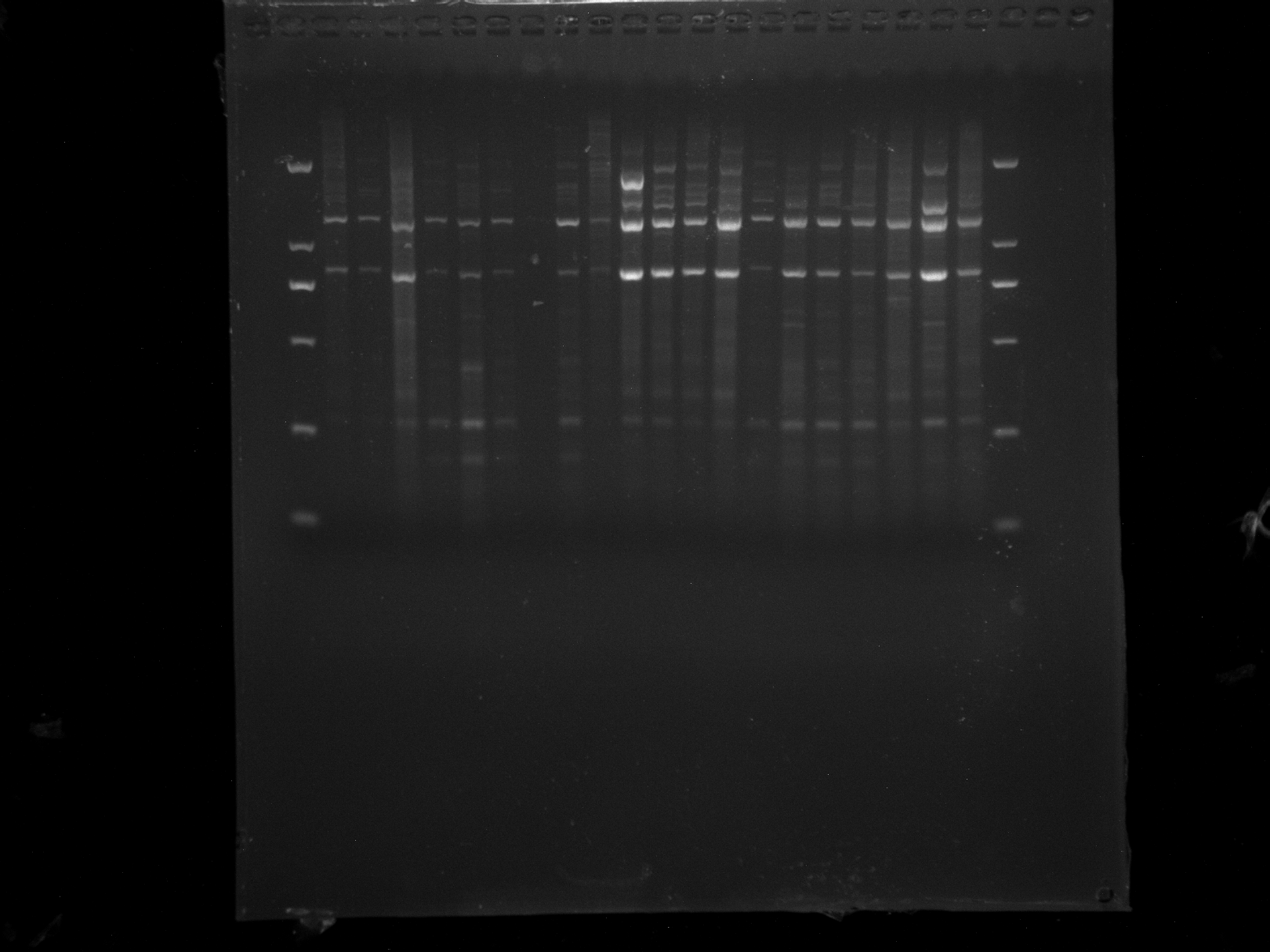

Supplement: Supplemental Information 43 — Amplification results of ERF1 on DGD1-20 samples. [file peerj-08-8498-s043.png]

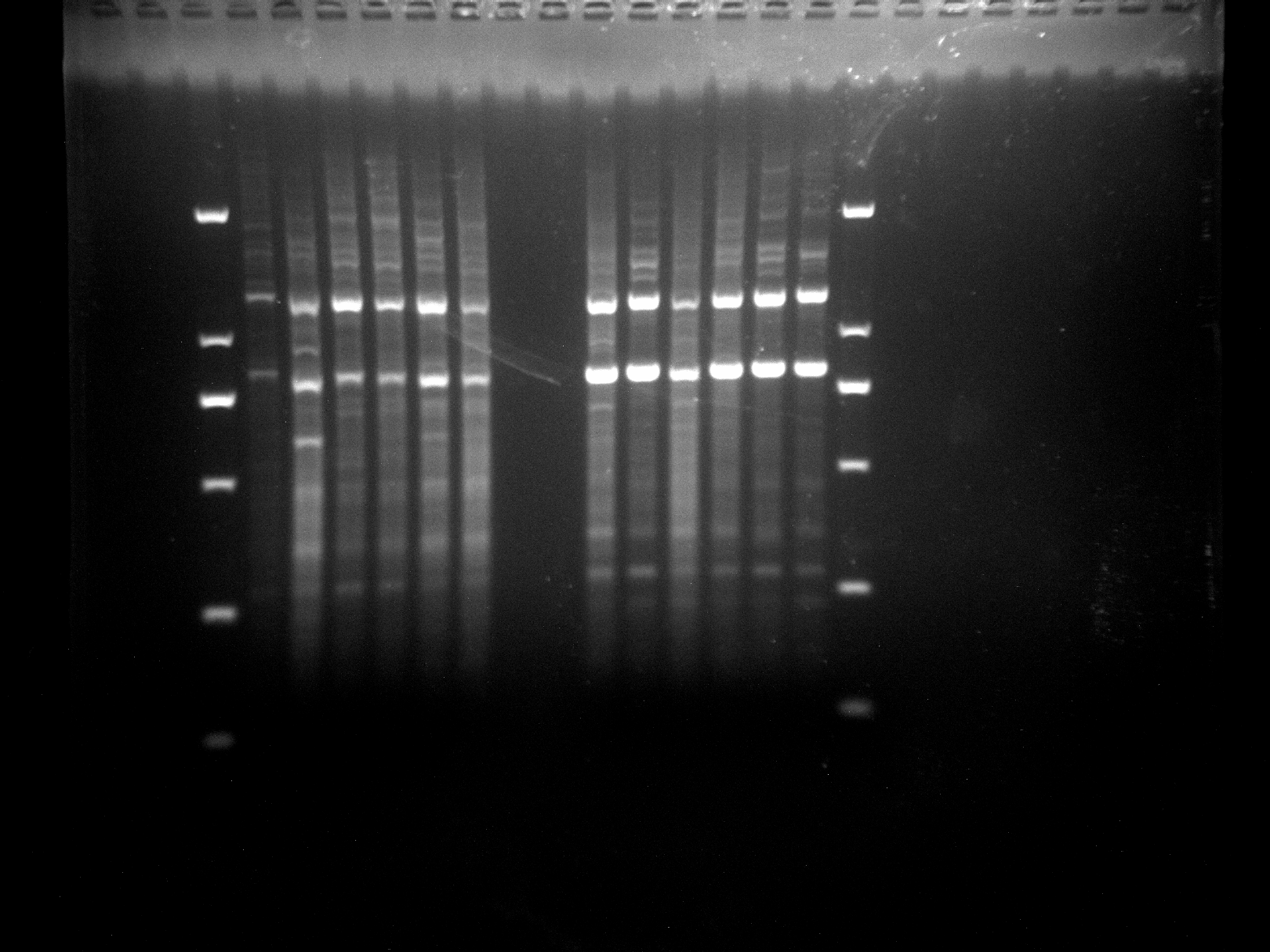

Supplement: Supplemental Information 44 — Amplification results of ERF1 on LS1-6, DGD21-26 samples. [file peerj-08-8498-s044.png]

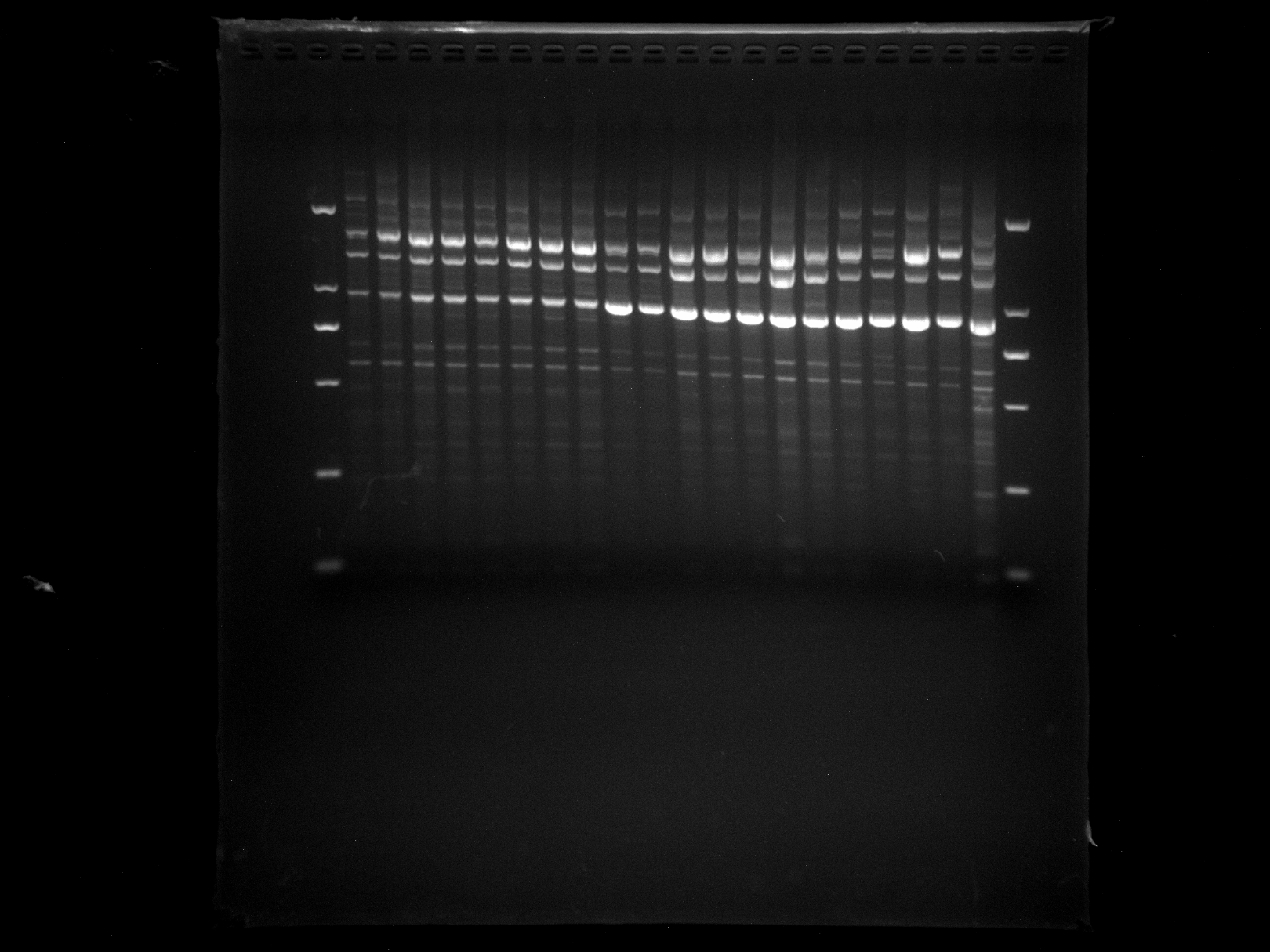

Supplement: Supplemental Information 45 — Amplification results of ERF2 on LGD1-8, DRS9-20 samples. [file peerj-08-8498-s045.png]

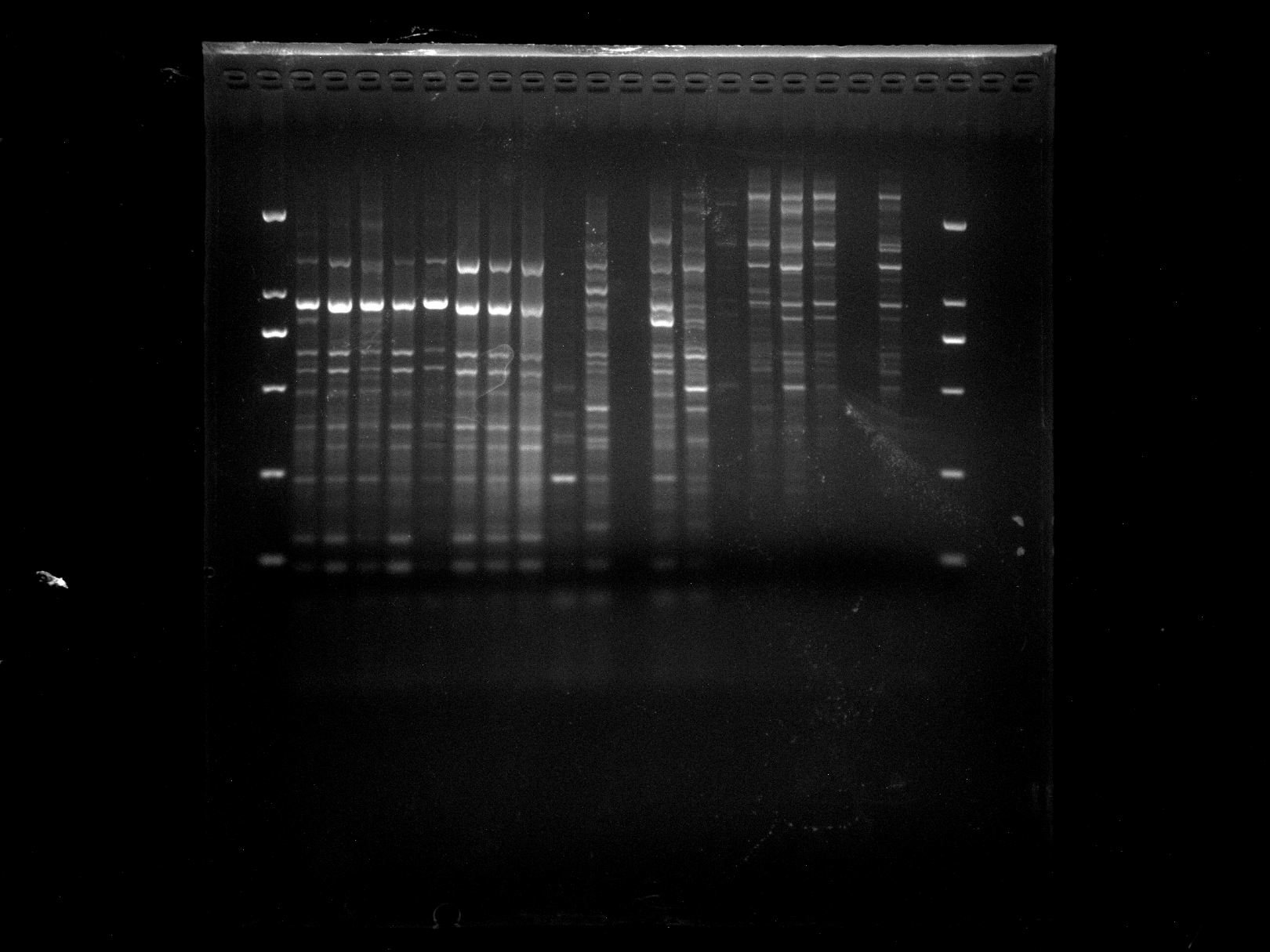

Supplement: Supplemental Information 46 — Amplification results of ERF2 on DRS21-28, NJD1-12 samples. [file peerj-08-8498-s046.png]

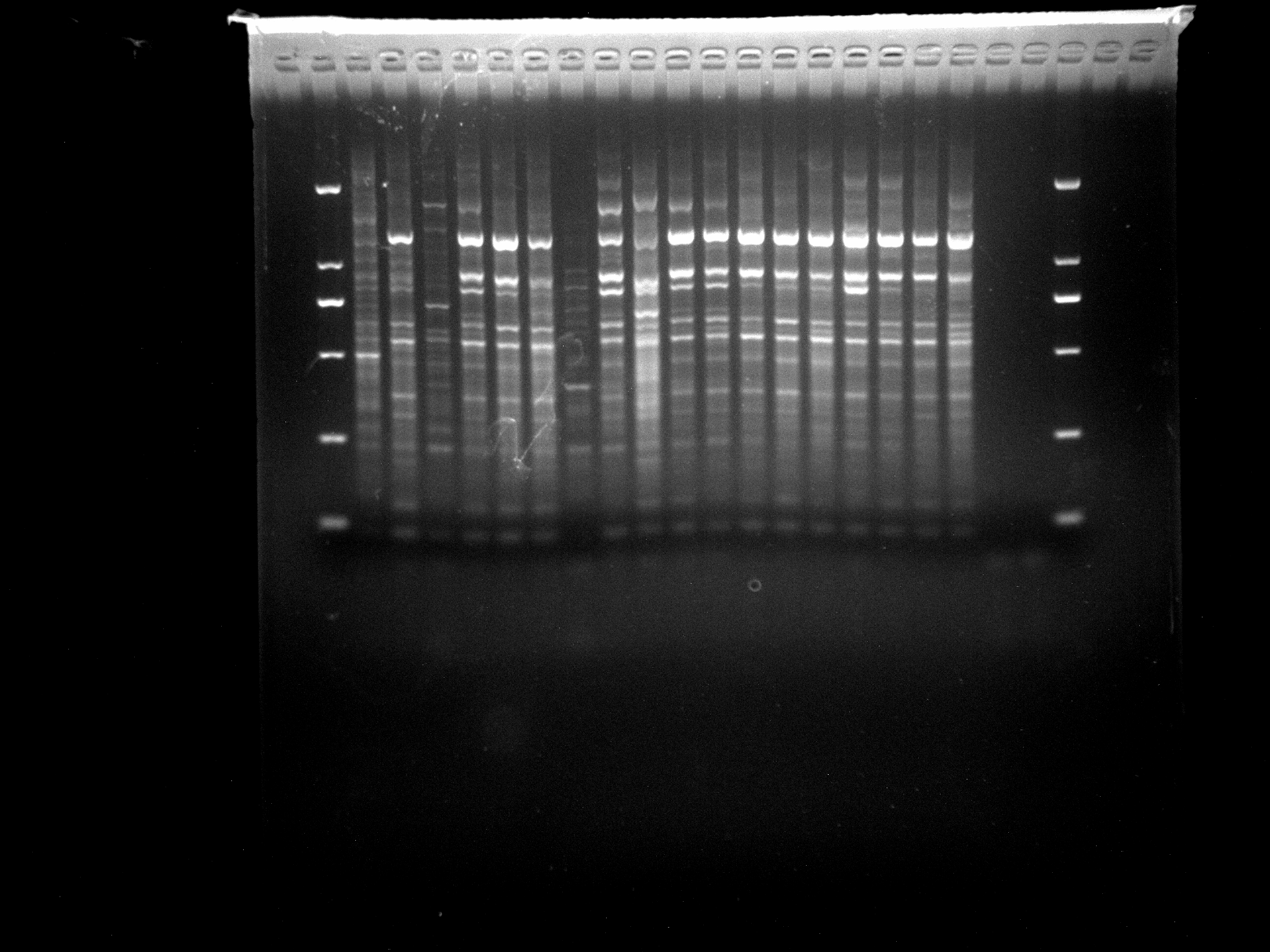

Supplement: Supplemental Information 47 — Amplification results of ERF2 on NJD13-32 samples. [file peerj-08-8498-s047.png]

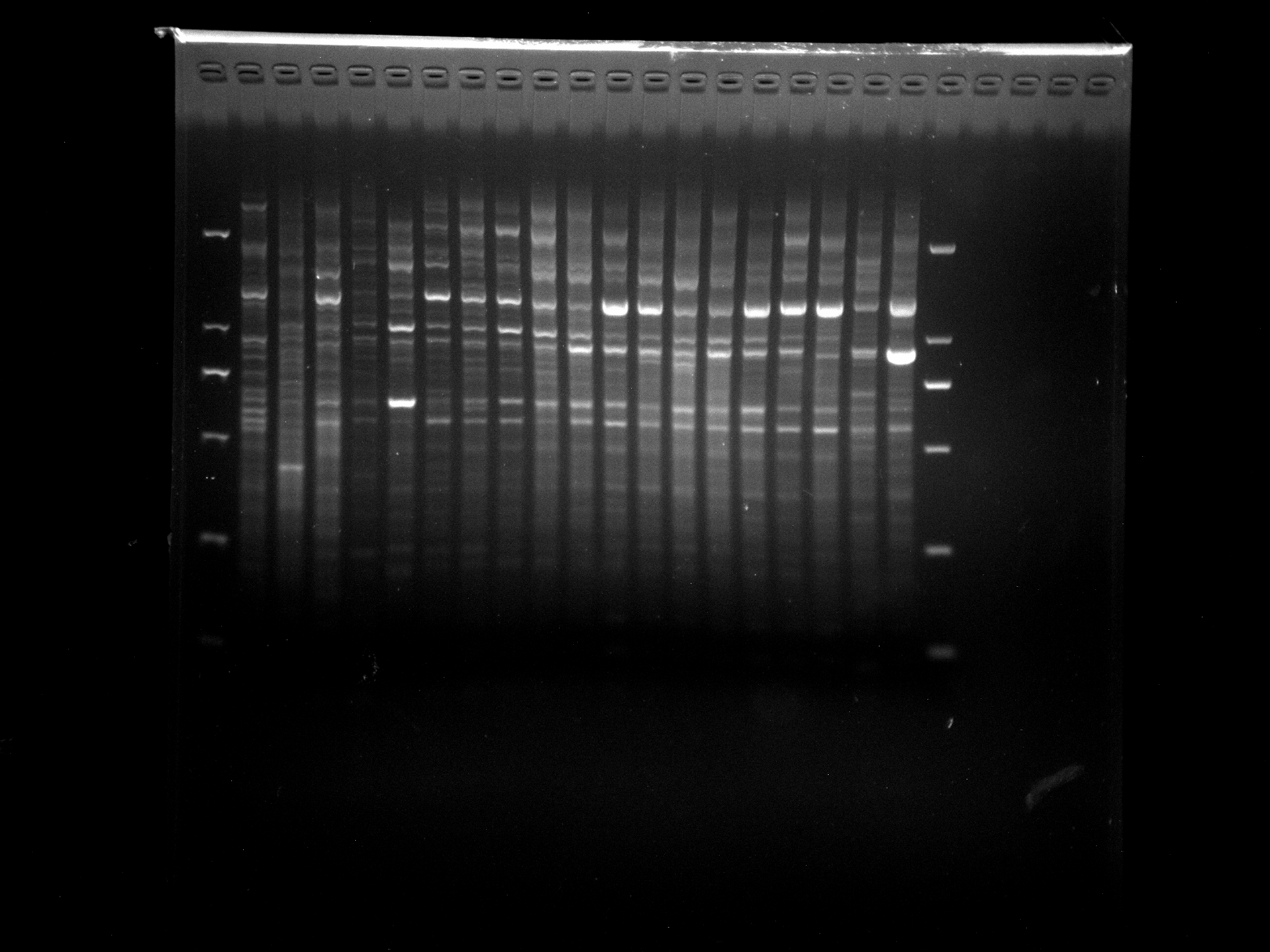

Supplement: Supplemental Information 48 — Amplification results of ERF2 on NJD33, LS1-13, PTD1, and LSD1 samples. [file peerj-08-8498-s048.png]

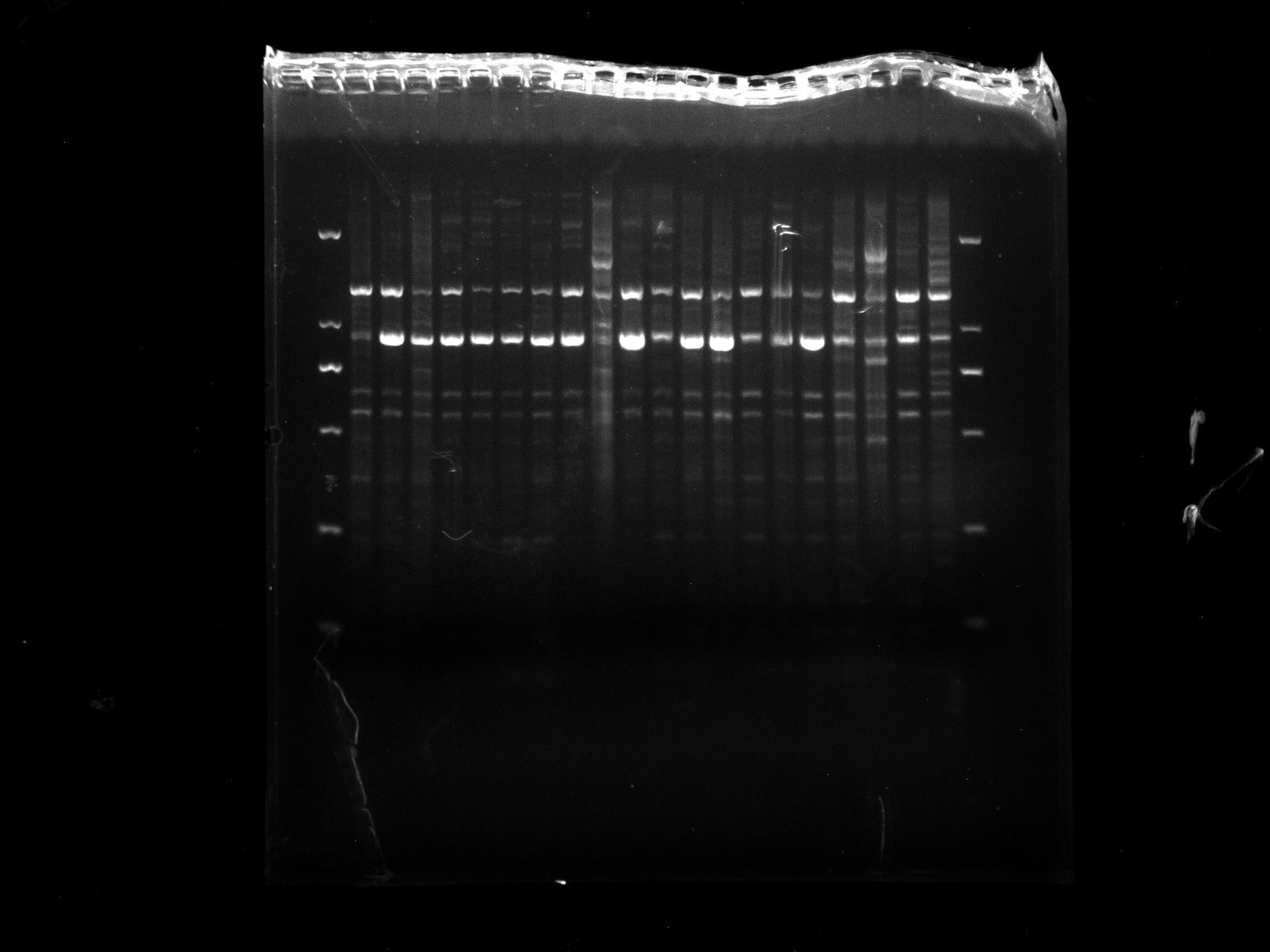

Supplement: Supplemental Information 49 — Amplification results of ERF2 on DGD1-20 samples. [file peerj-08-8498-s049.png]

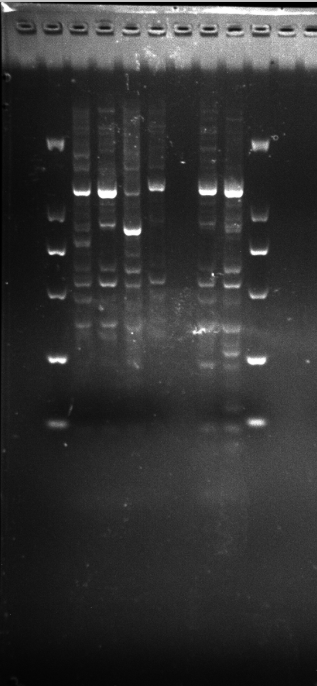

Supplement: Supplemental Information 50 — Amplification results of ERF2 on DGD21-26 samples. [file peerj-08-8498-s050.png]

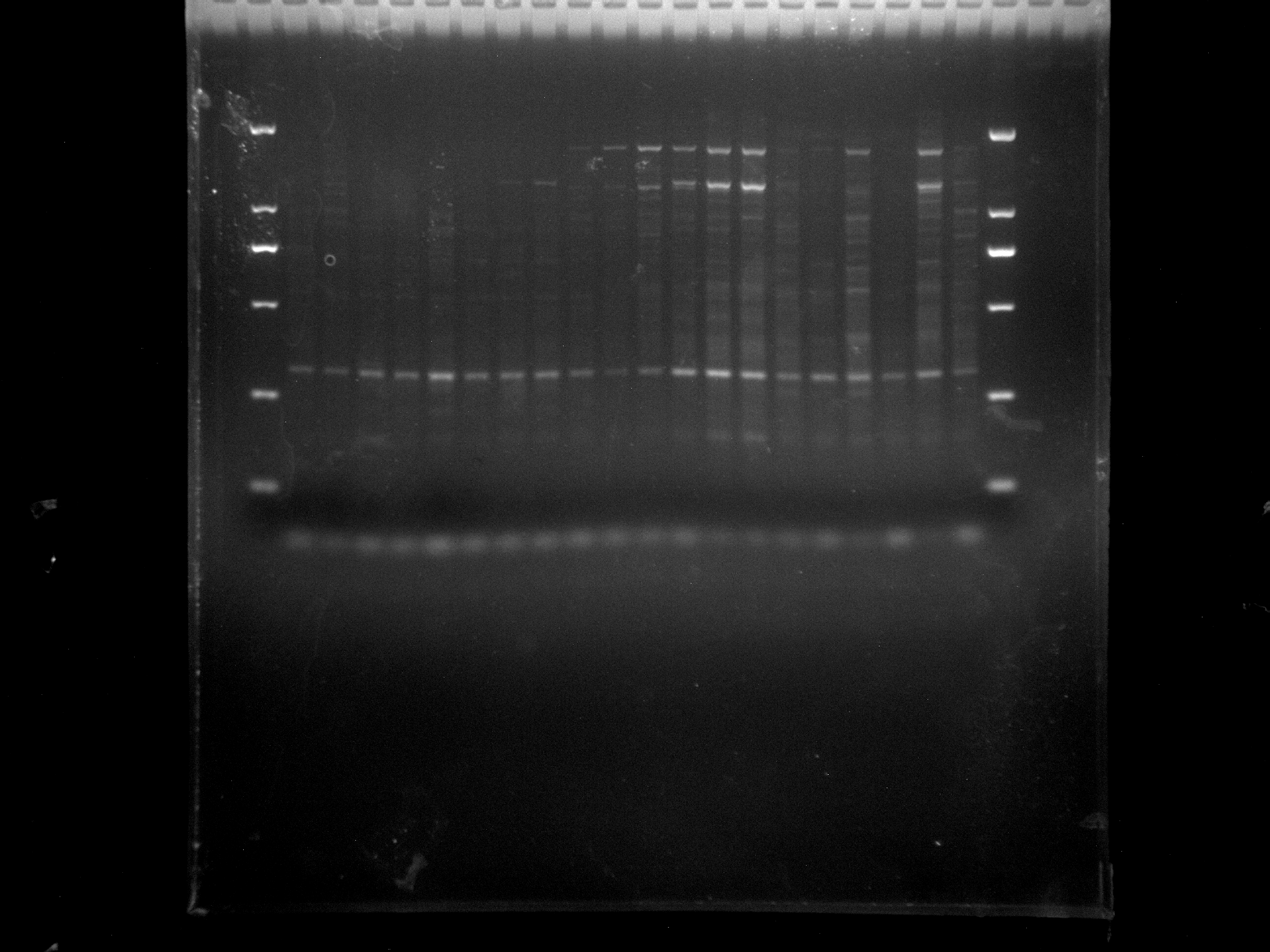

Supplement: Supplemental Information 51 — Amplification results of ERF3 on LGD1-8, DRS9-20 samples. [file peerj-08-8498-s051.png]

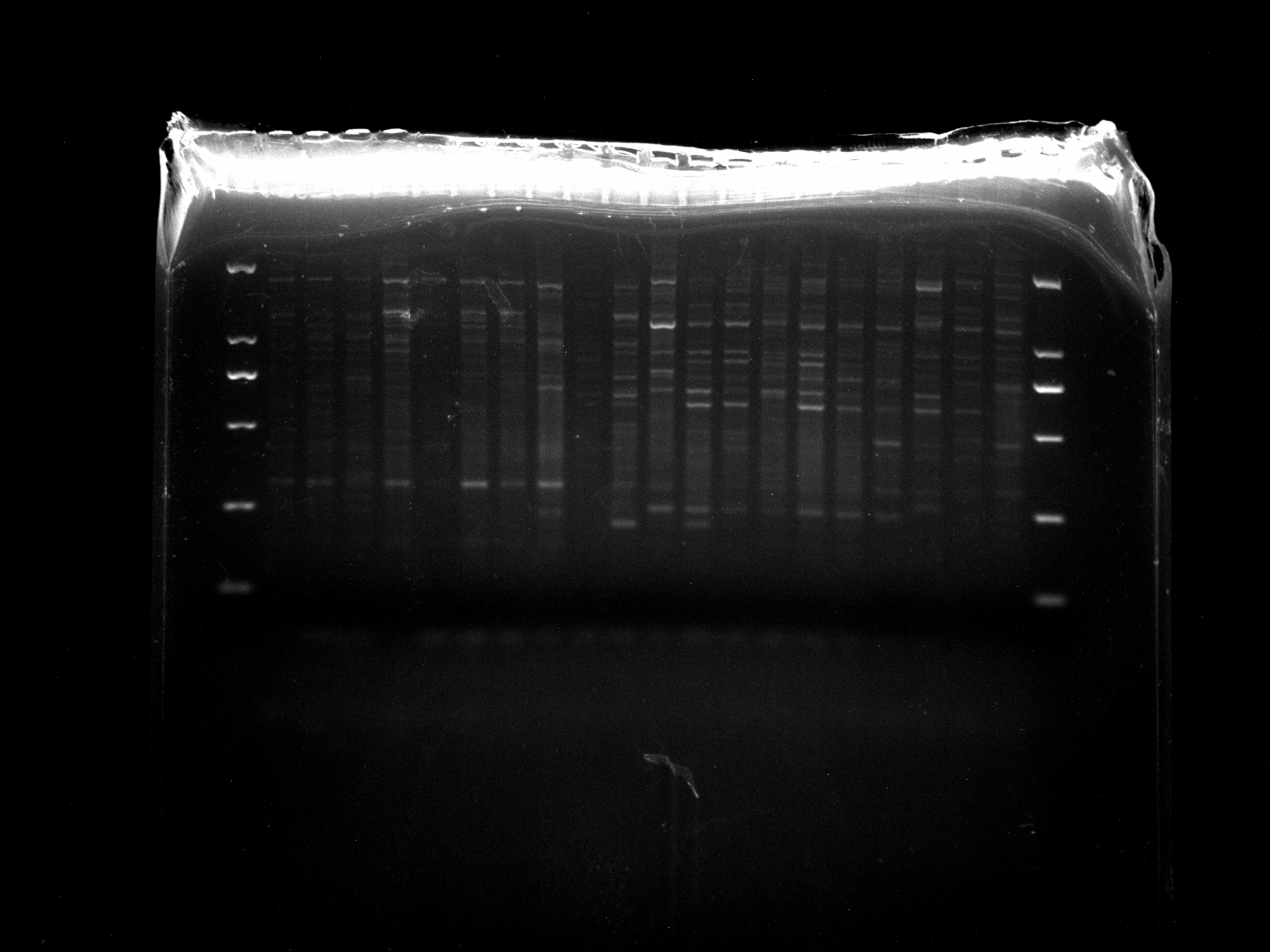

Supplement: Supplemental Information 52 — Amplification results of ERF3 on DRS21-28, NJD1-12 samples. [file peerj-08-8498-s052.png]

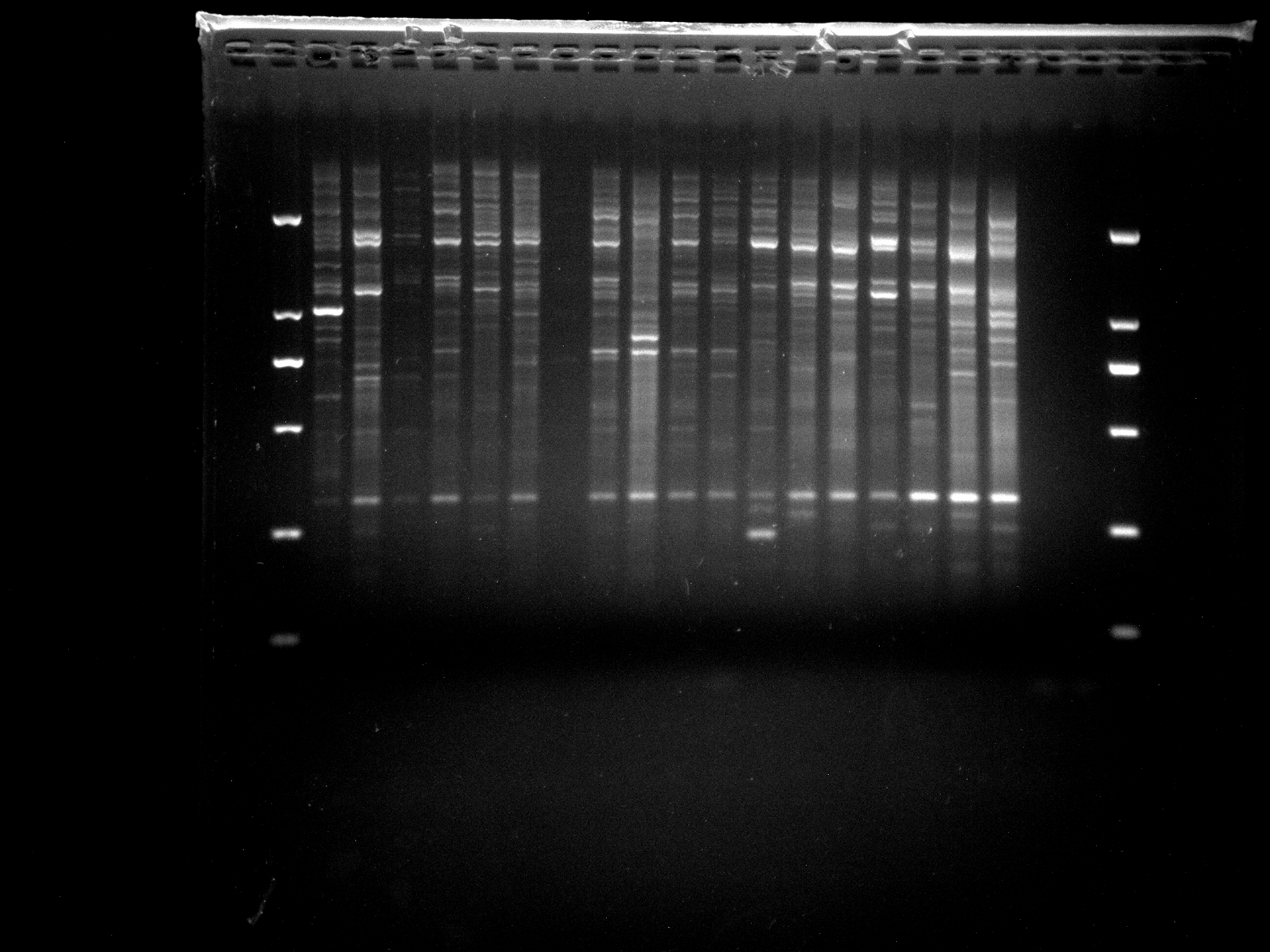

Supplement: Supplemental Information 53 — Amplification results of ERF3 on NJD13-32 samples. [file peerj-08-8498-s053.png]

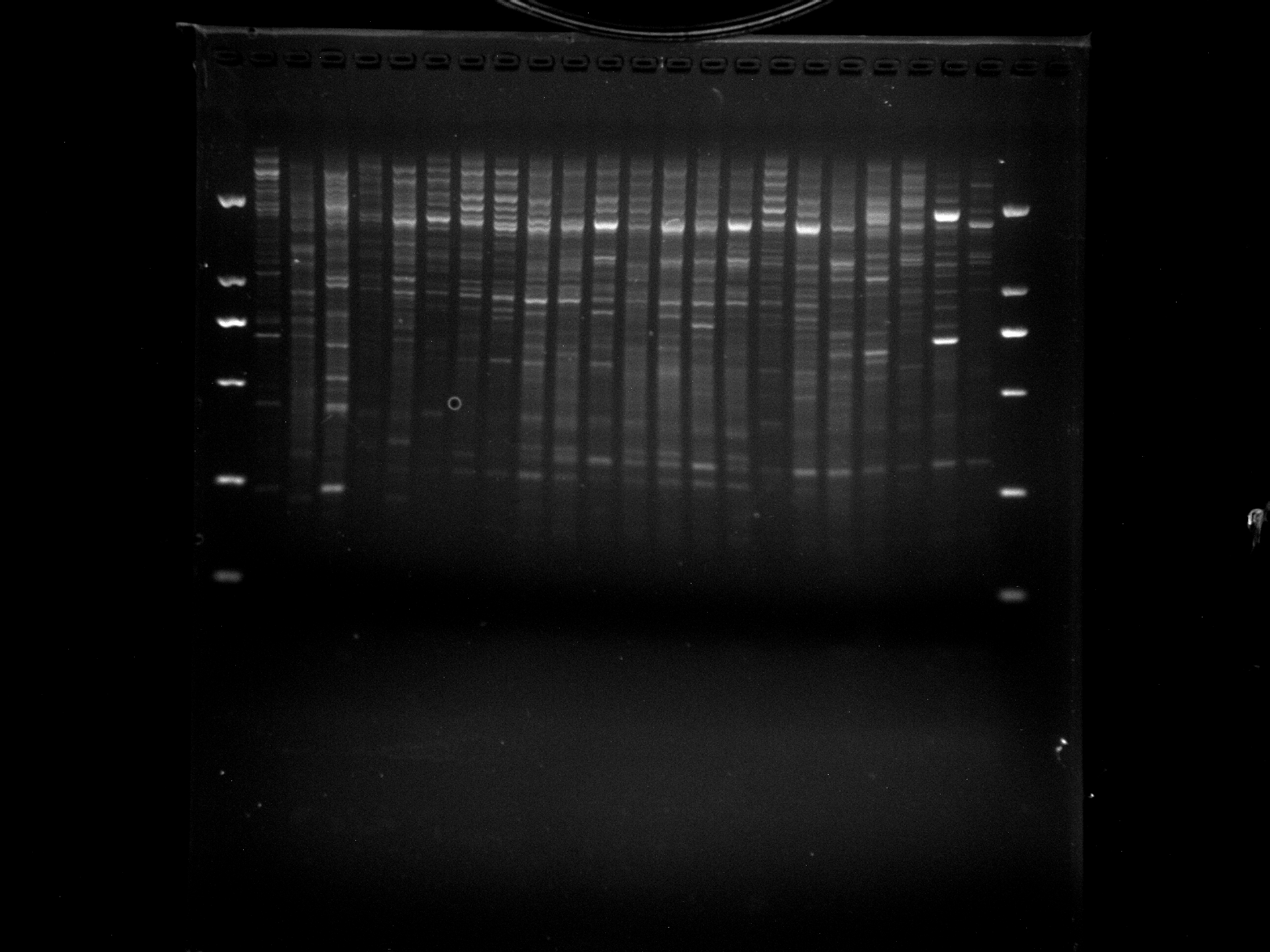

Supplement: Supplemental Information 54 — Amplification results of ERF3 on NJD33, LS1-13, PTU1, LSD1, and DGD24-26 samples. [file peerj-08-8498-s054.png]

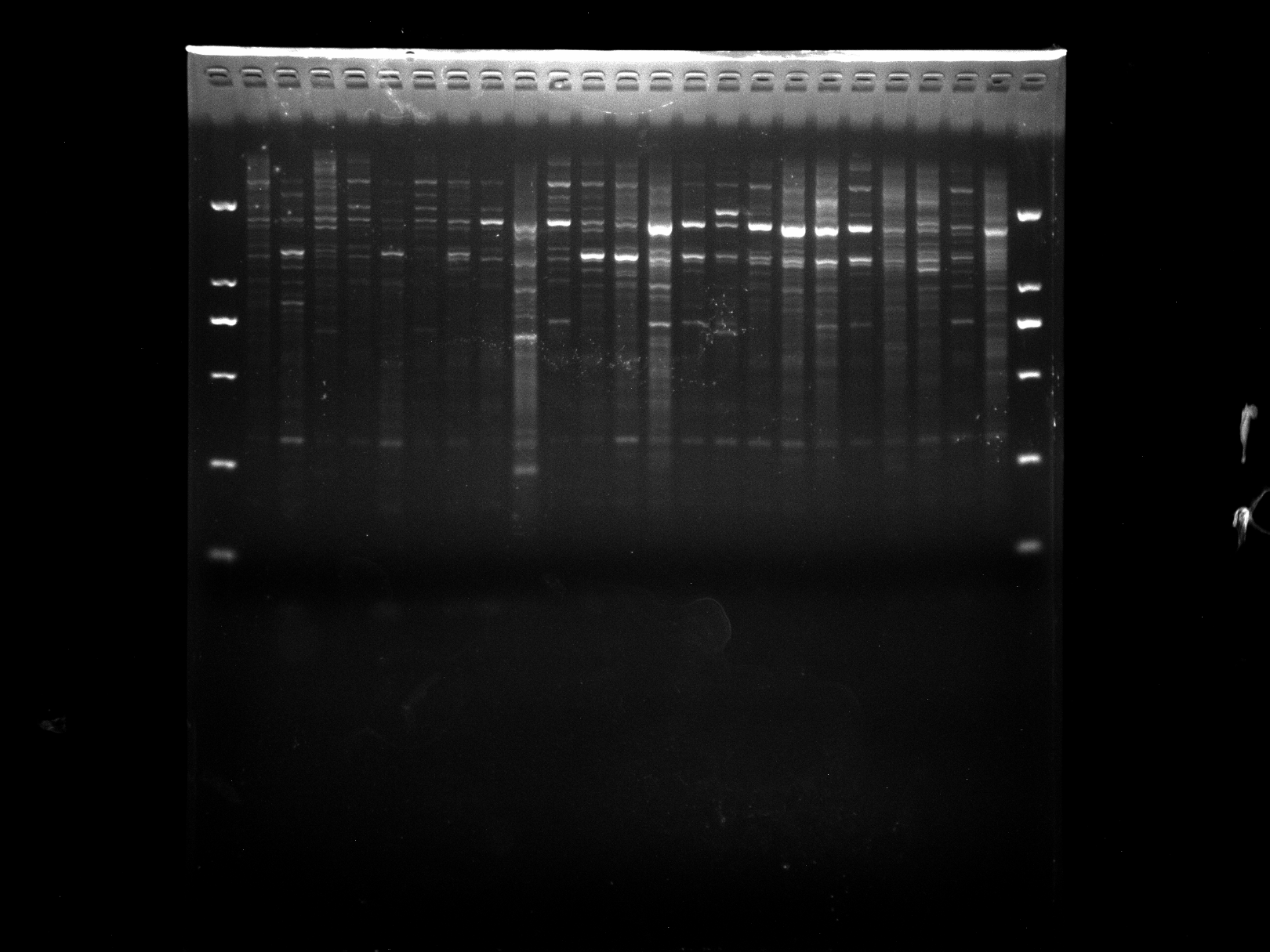

Supplement: Supplemental Information 55 — Amplification results of ERF3 on DGD1-23 samples. [file peerj-08-8498-s055.png]

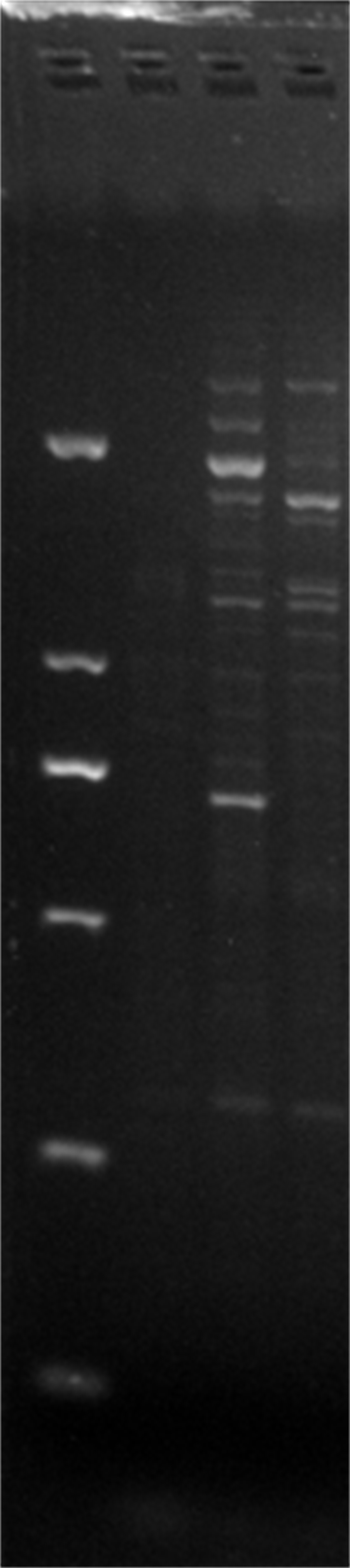

Supplement: Supplemental Information 56 — Amplification results of ERF3 on DGD24-26 samples. [file peerj-08-8498-s056.png]

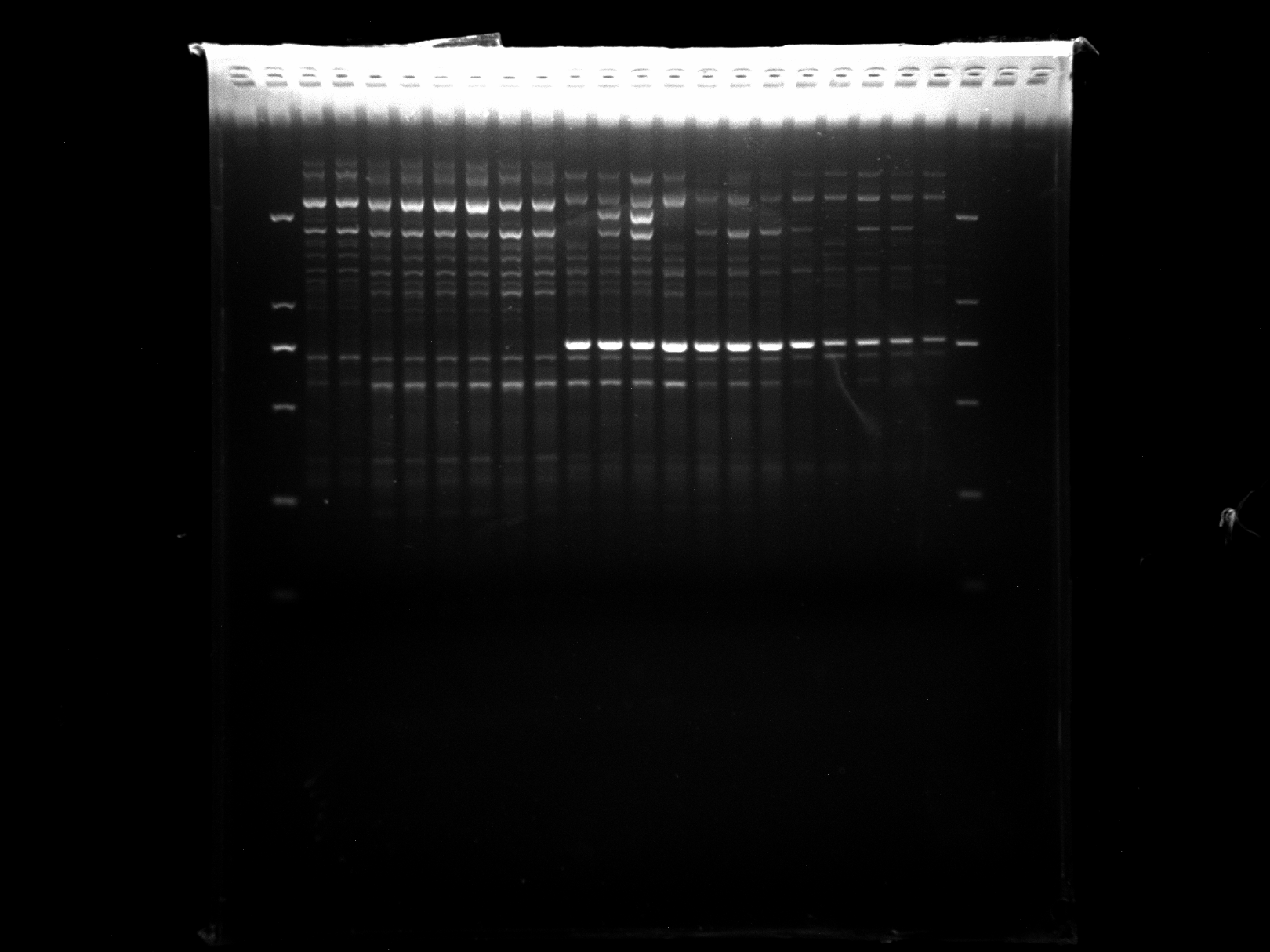

Supplement: Supplemental Information 57 — KNOX-1 amplification results for LGD1-8, DRS9-20 samples. [file peerj-08-8498-s057.png]

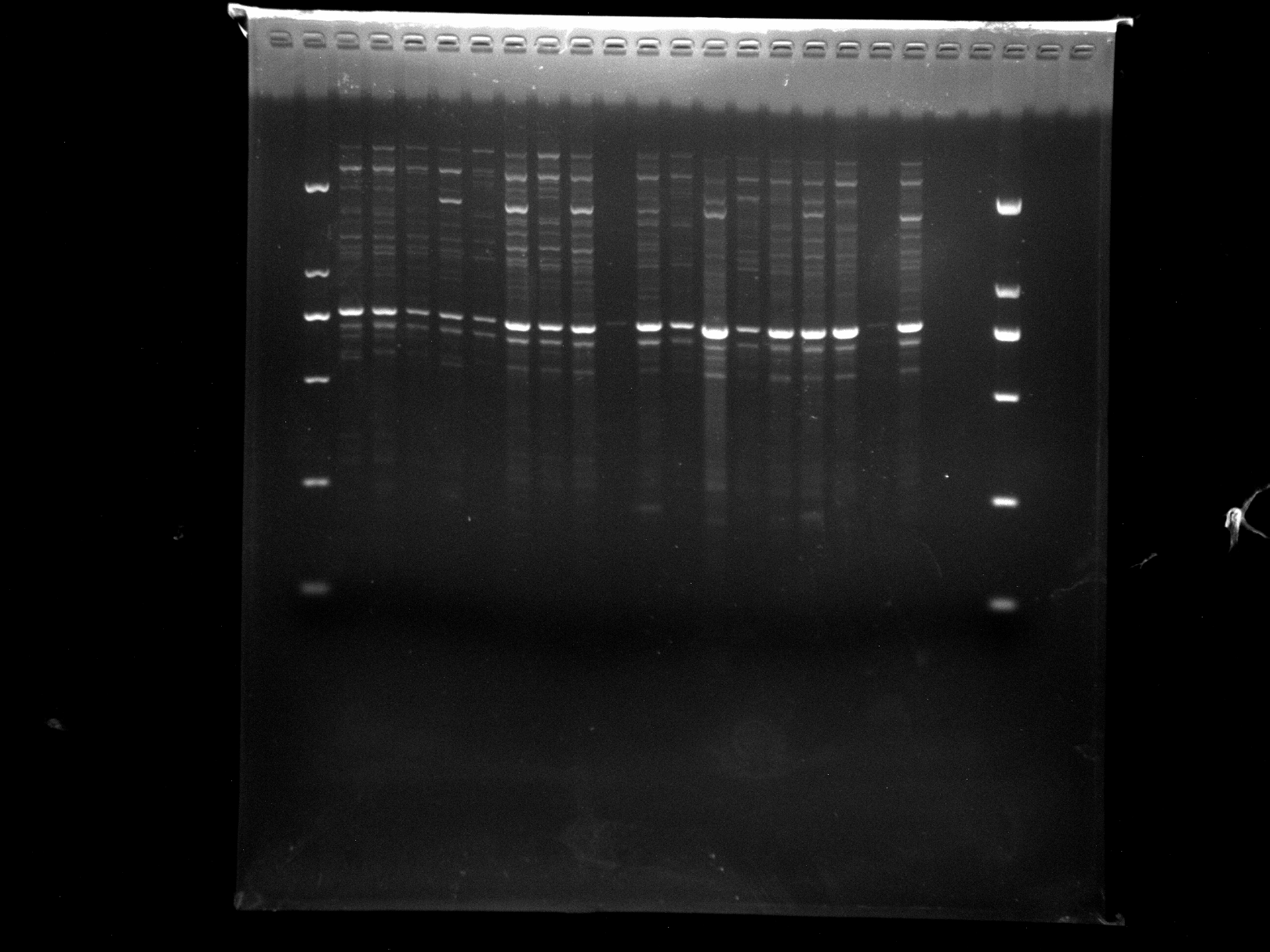

Supplement: Supplemental Information 58 — KNOX-1 amplification results for DRS21-28, NJD1-12 samples. [file peerj-08-8498-s058.png]

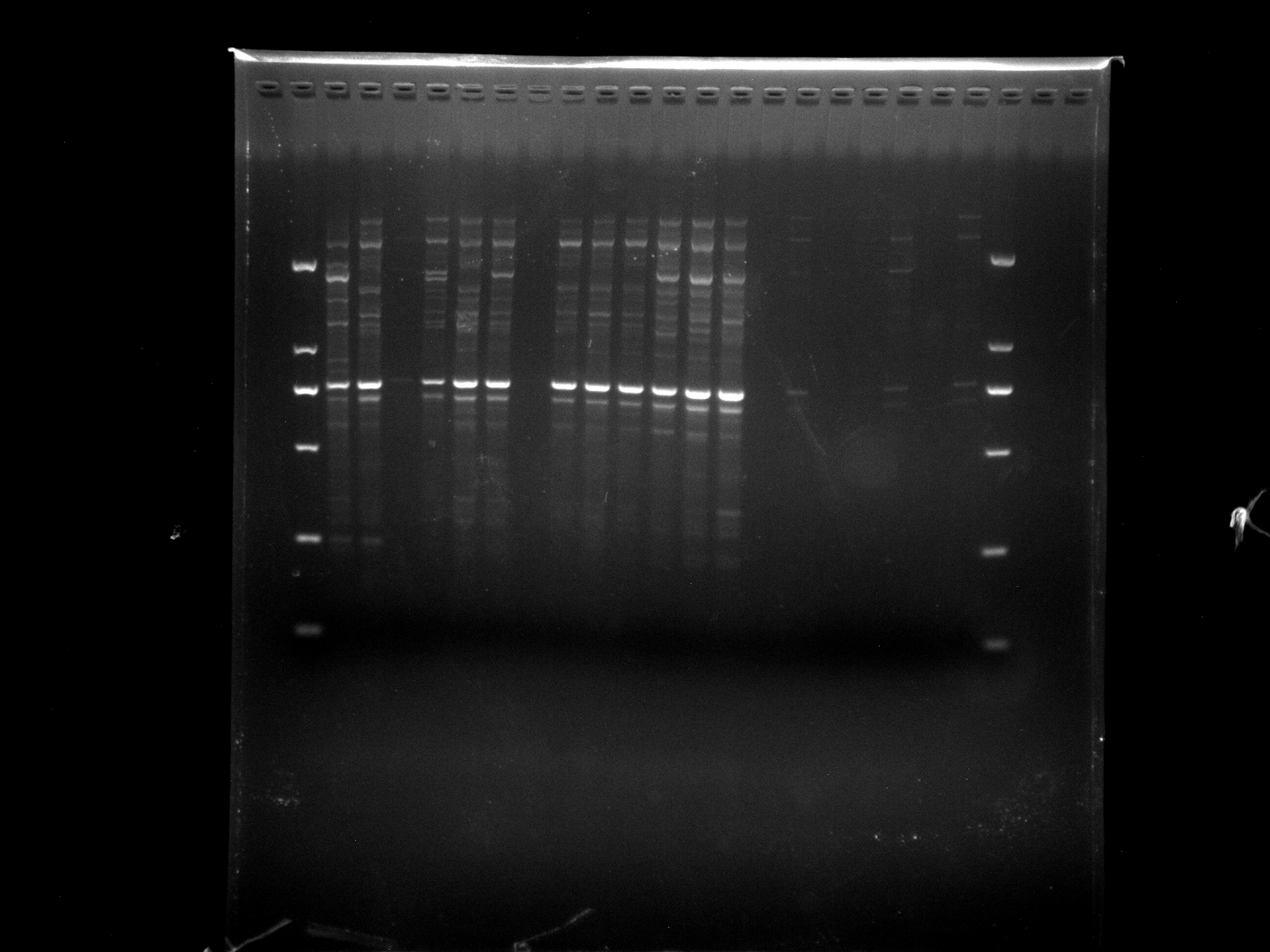

Supplement: Supplemental Information 59 — KNOX-1 amplification results for NJD13-32 samples. [file peerj-08-8498-s059.png]

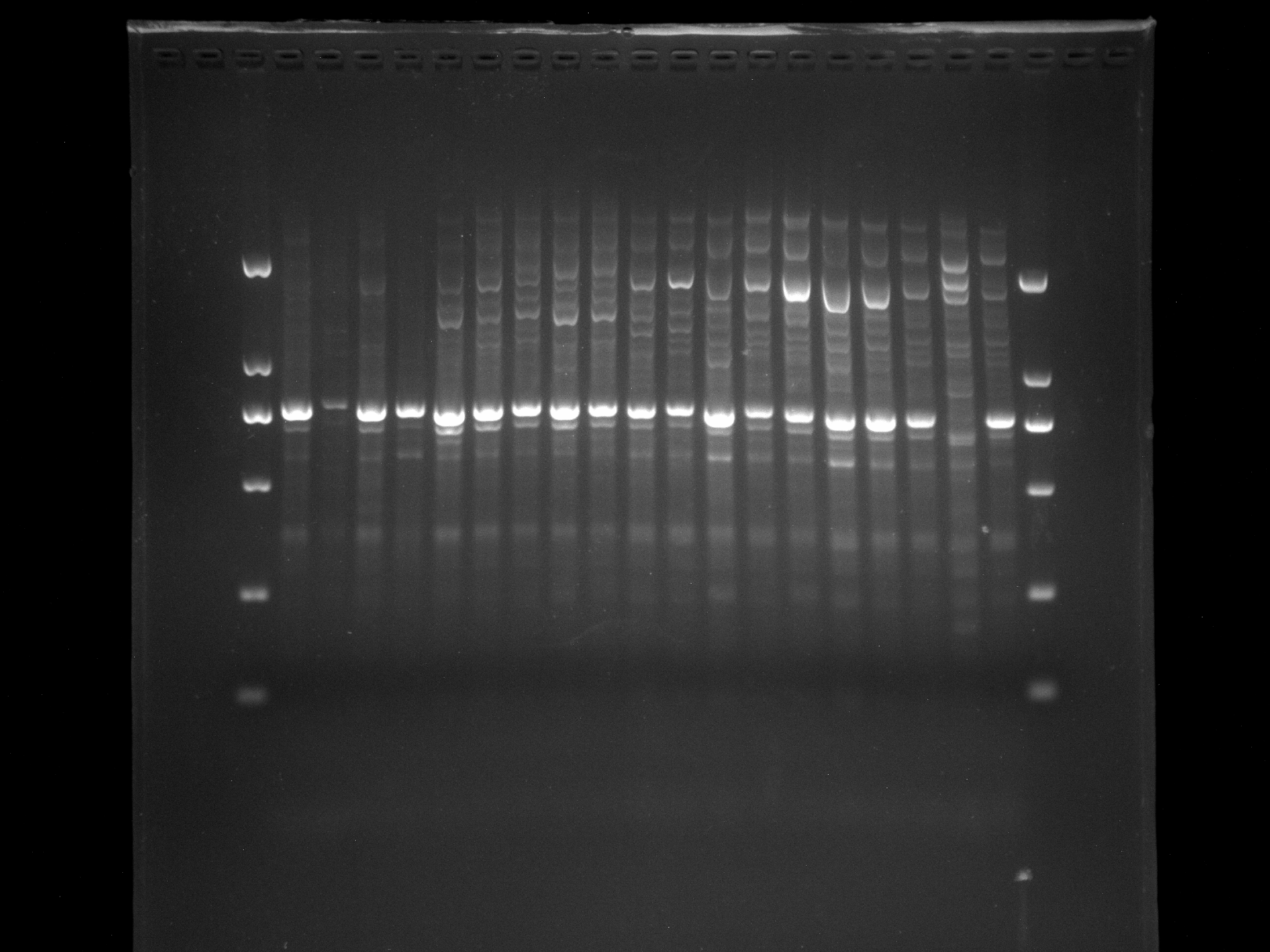

Supplement: Supplemental Information 60 — KNOX-1 amplification results for NJD33, LS1-13, PUD1, LSD1 samples. [file peerj-08-8498-s060.png]

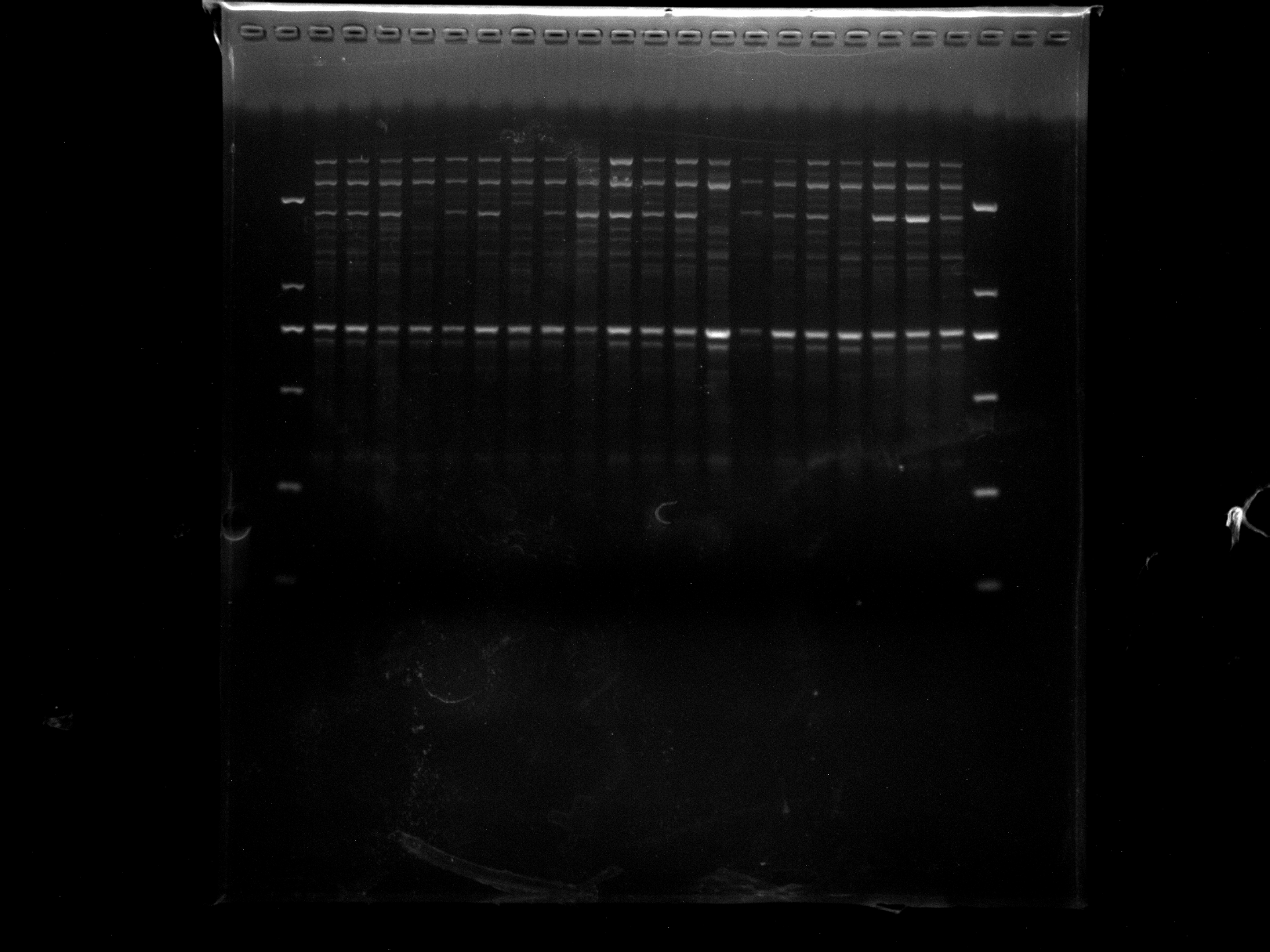

Supplement: Supplemental Information 61 — KNOX-1 amplification results for DGD1-20 samples. [file peerj-08-8498-s061.png]

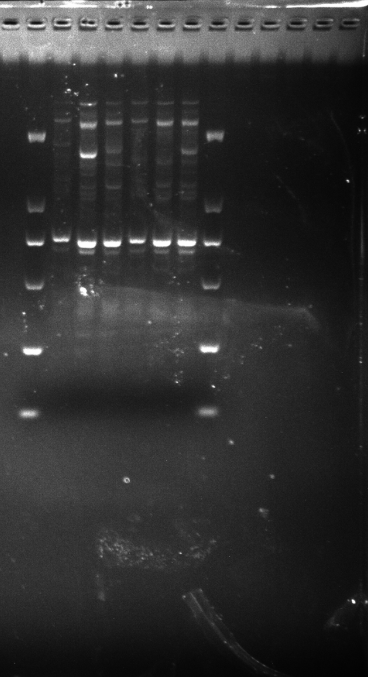

Supplement: Supplemental Information 62 — KNOX-1 amplification results for DGD21-26 samples. [file peerj-08-8498-s062.png]

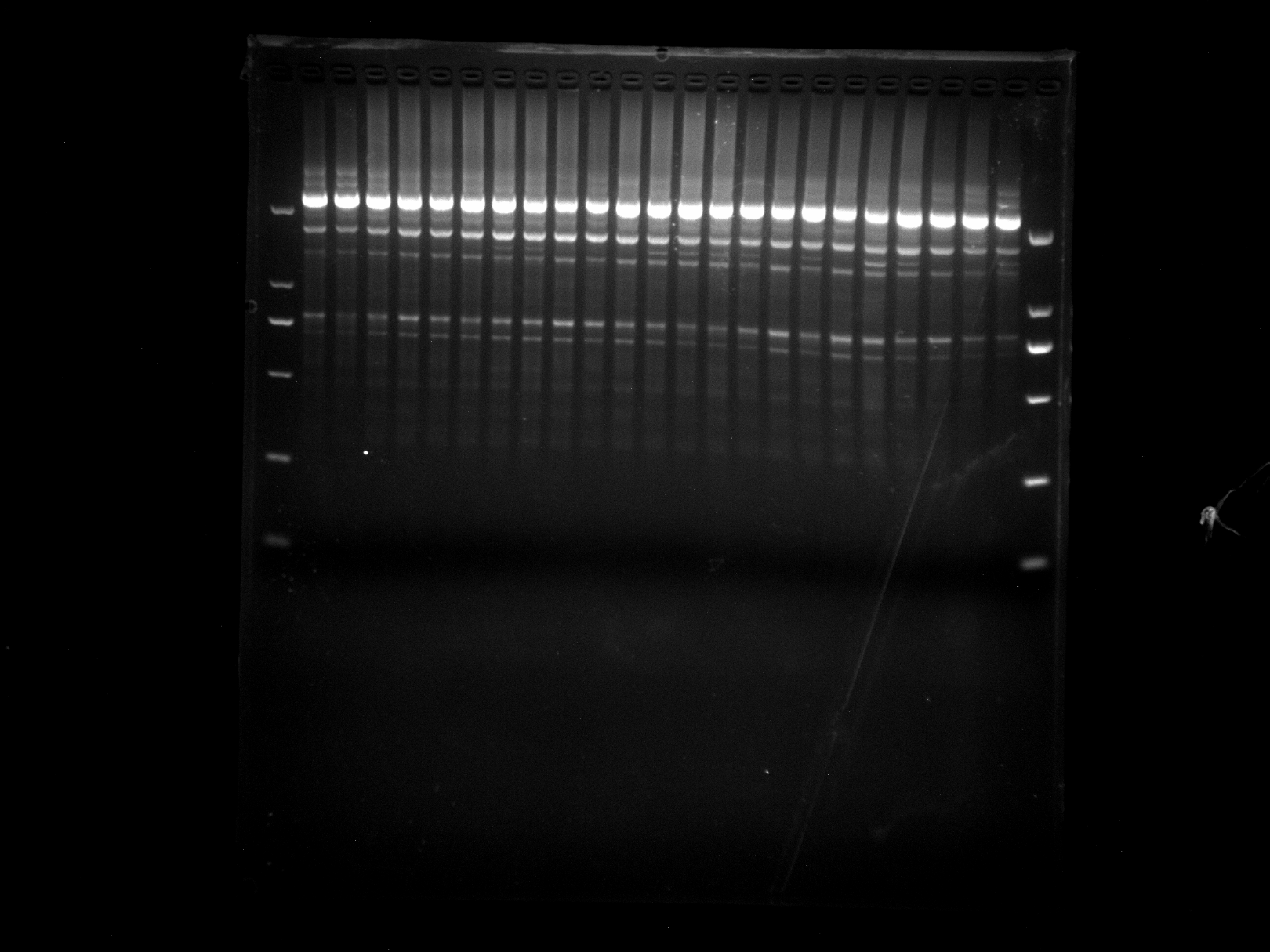

Supplement: Supplemental Information 63 — KNOX-2 amplification results for LGD1-8, DRS9-23 samples. [file peerj-08-8498-s063.png]

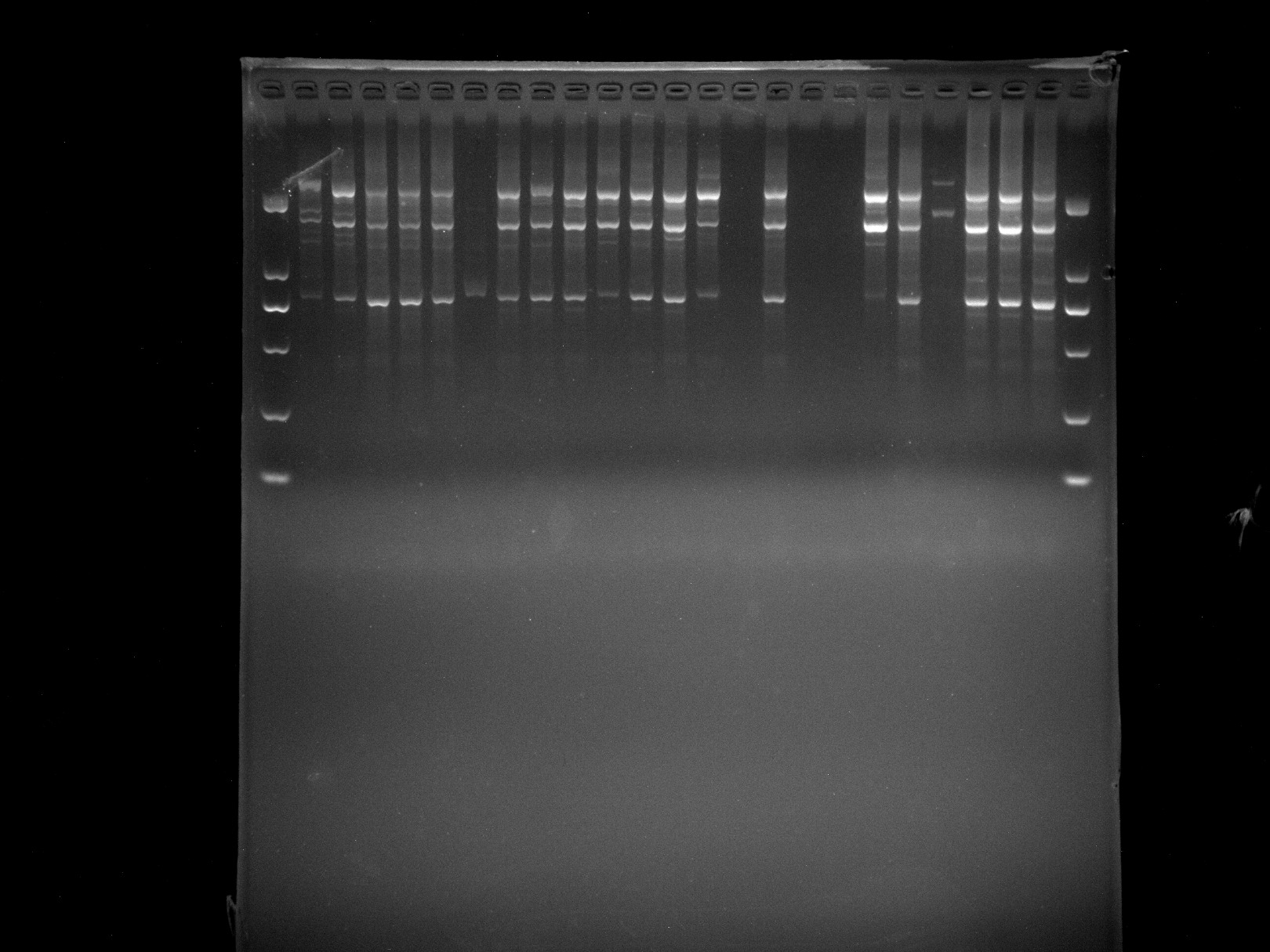

Supplement: Supplemental Information 64 — KNOX-2 amplification results for DRS24-28, NJD1-18 samples. [file peerj-08-8498-s064.png]

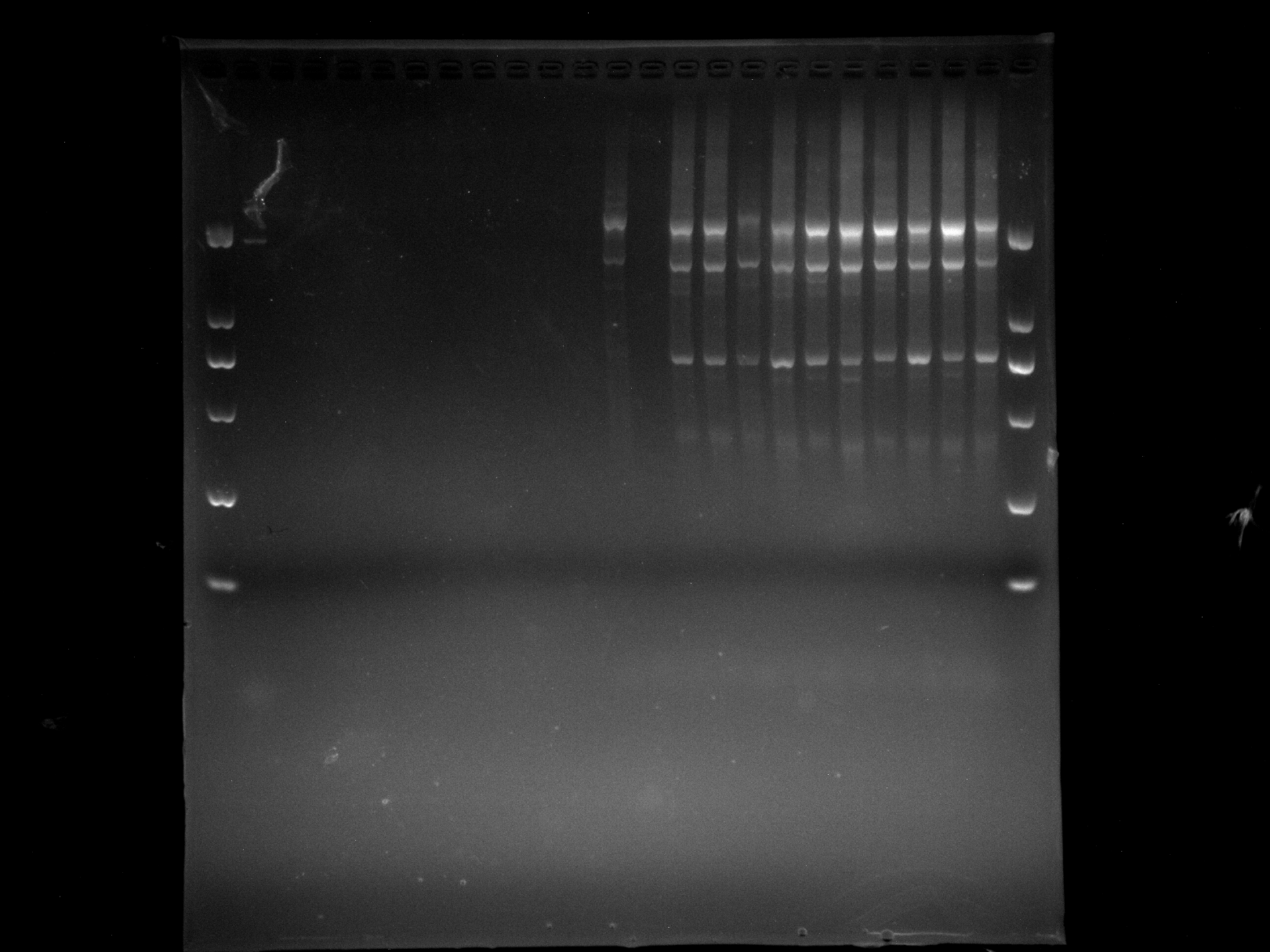

Supplement: Supplemental Information 65 — KNOX-2 amplification results for NJD19-33, LS1-6 samples. [file peerj-08-8498-s065.png]

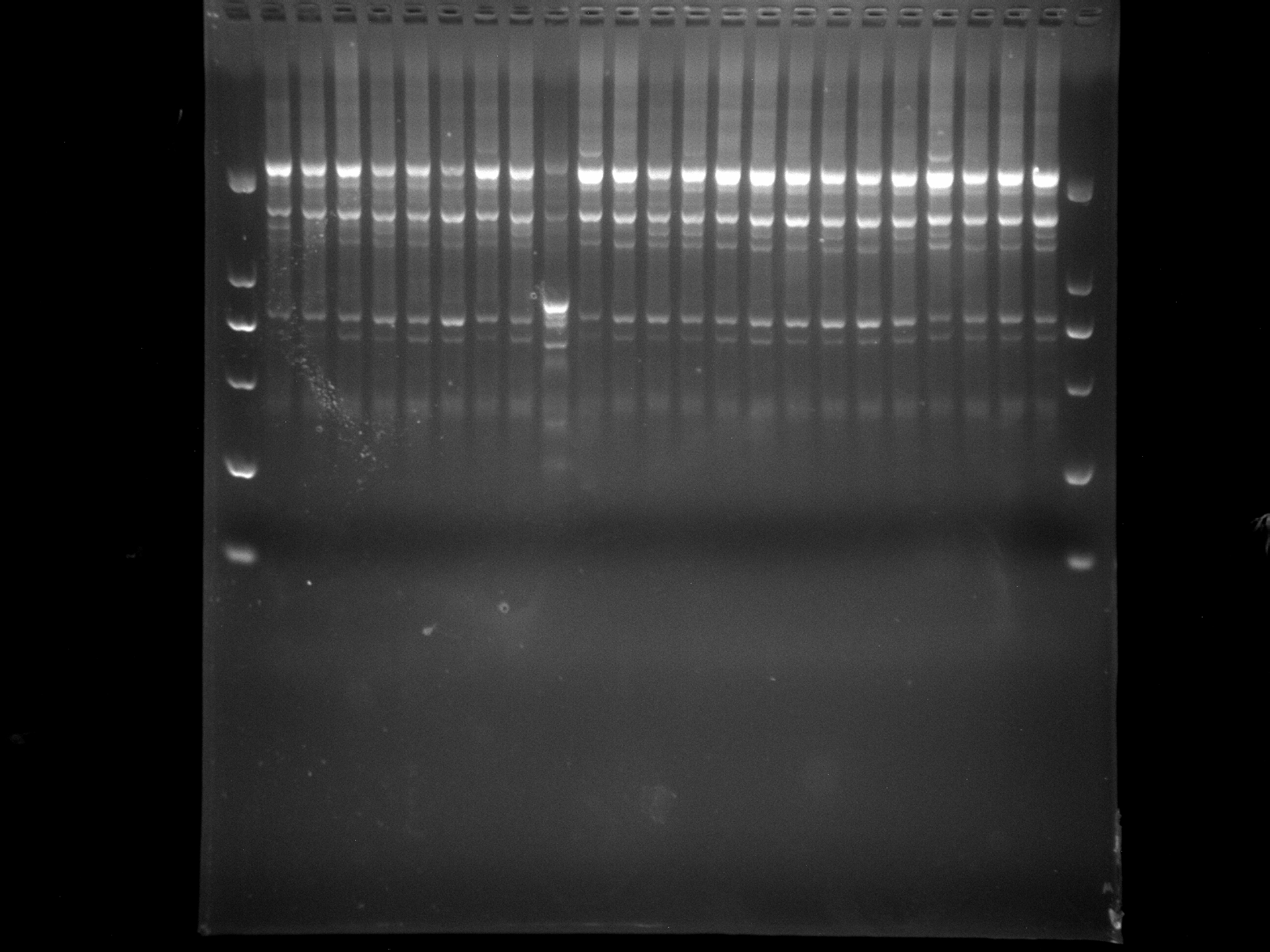

Supplement: Supplemental Information 66 — KNOX-2 amplification results for LS7-13, PTD1, LSD1and DGD1-13 samples. [file peerj-08-8498-s066.png]

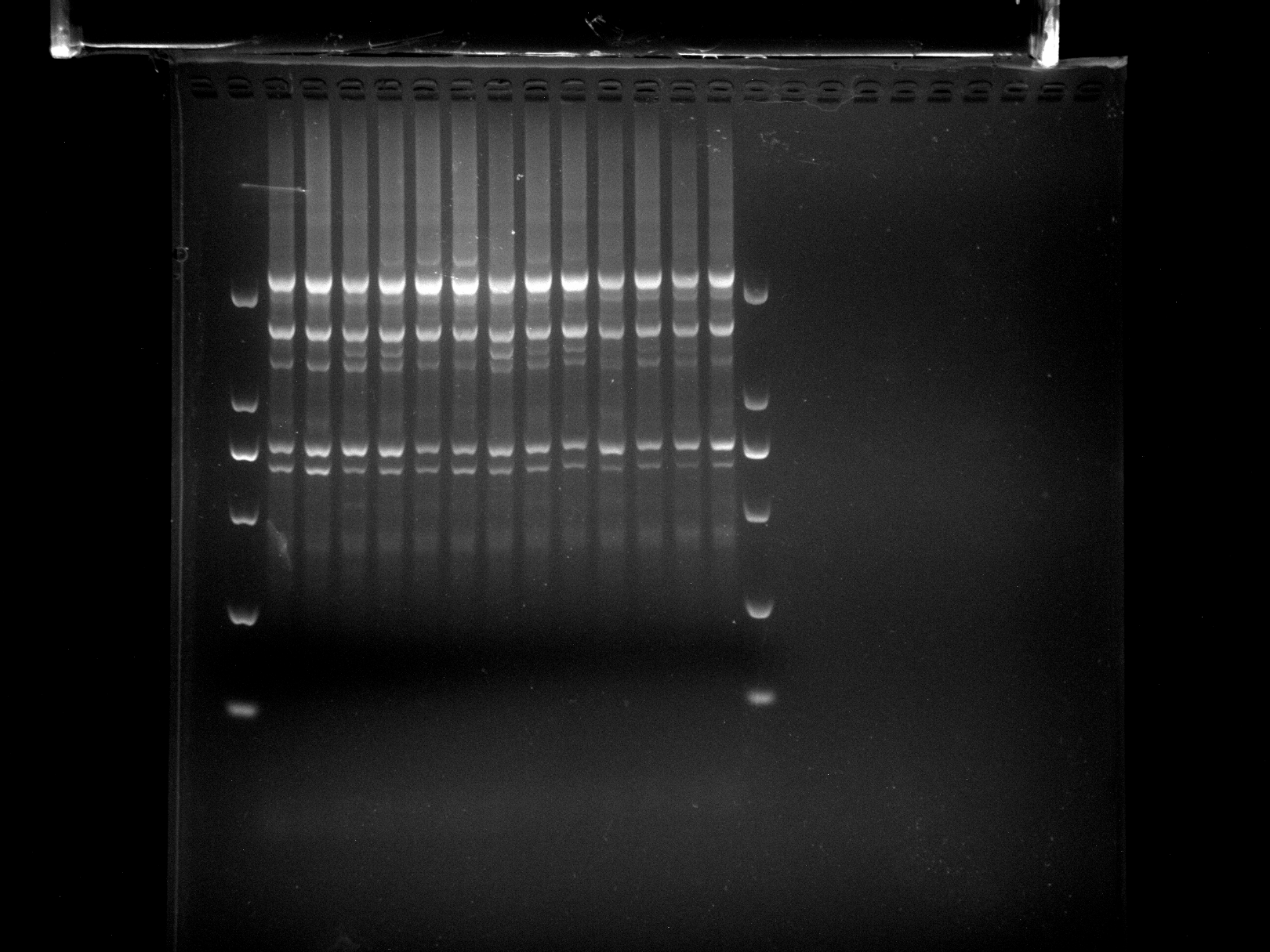

Supplement: Supplemental Information 67 — KNOX-2 amplification results for DGD14-26 samples. [file peerj-08-8498-s067.png]

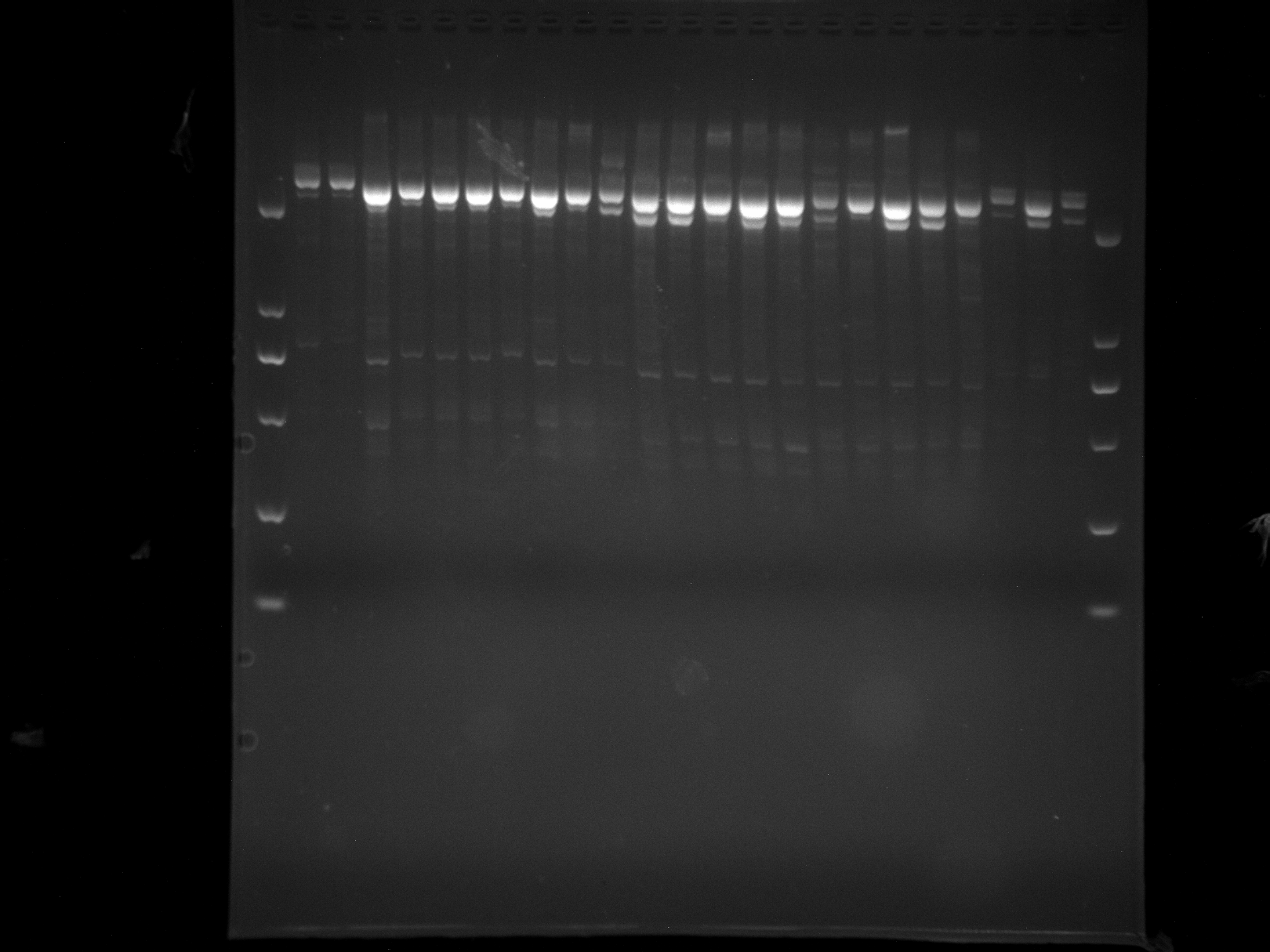

Supplement: Supplemental Information 68 — KNOX-3 amplification results for LGD1-8, DRS9-23 samples. [file peerj-08-8498-s068.png]

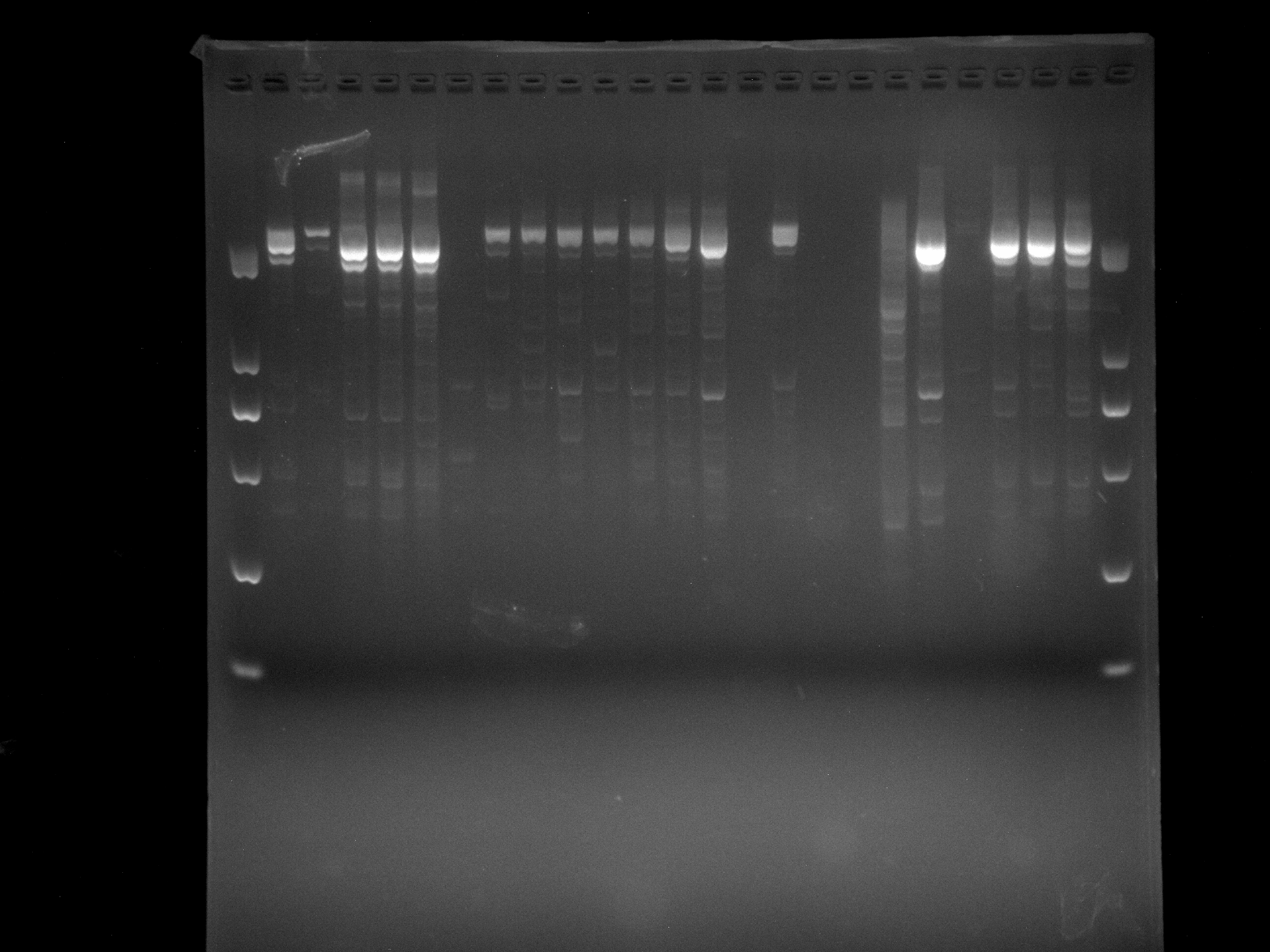

Supplement: Supplemental Information 69 — KNOX-3 amplification results for DRS24-28, NJD1-18 samples. [file peerj-08-8498-s069.png]

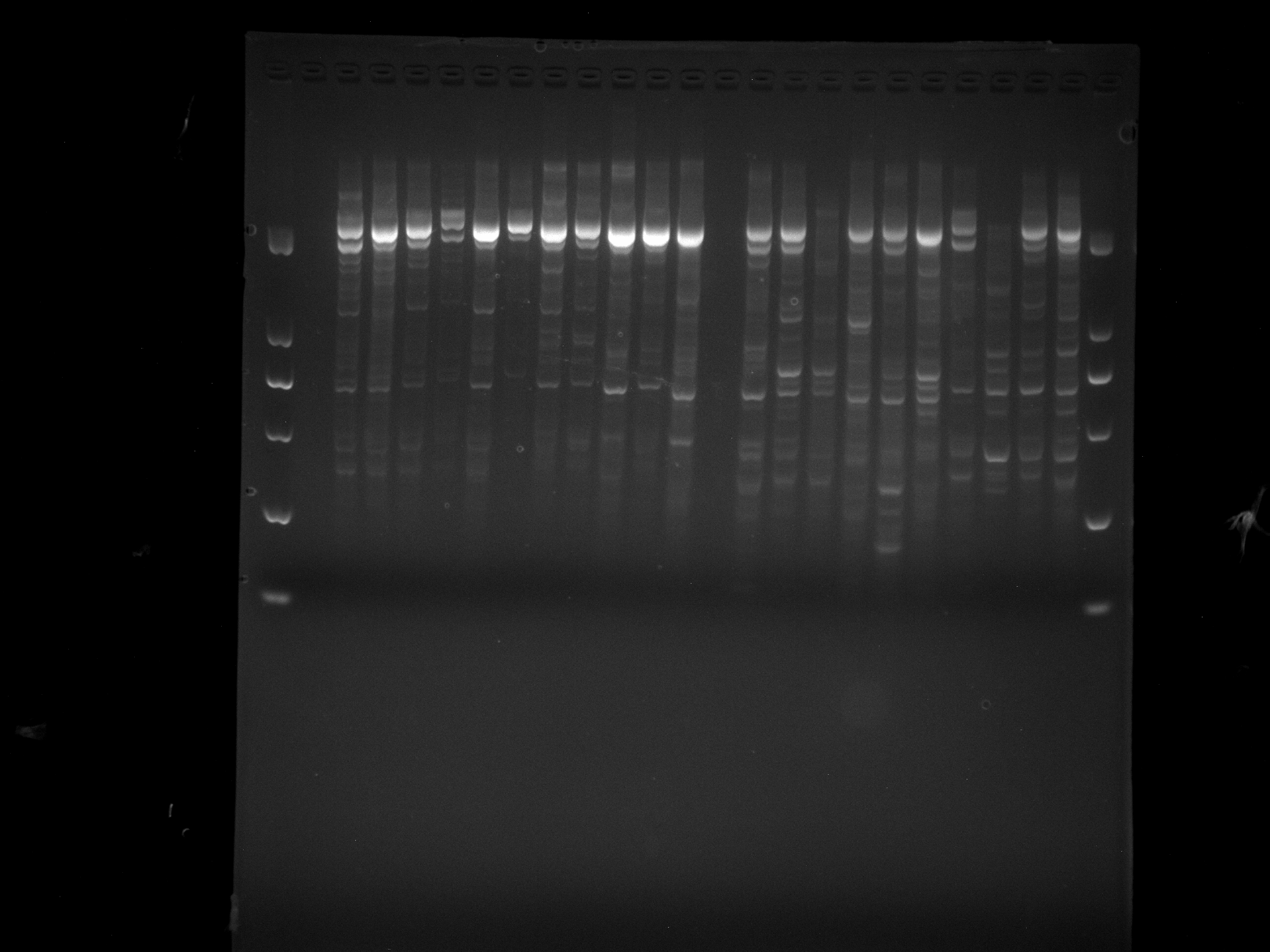

Supplement: Supplemental Information 70 — KNOX-3 amplification results for NJD19-33, PTD1, LSD1, LS1-4 samples. [file peerj-08-8498-s070.png]

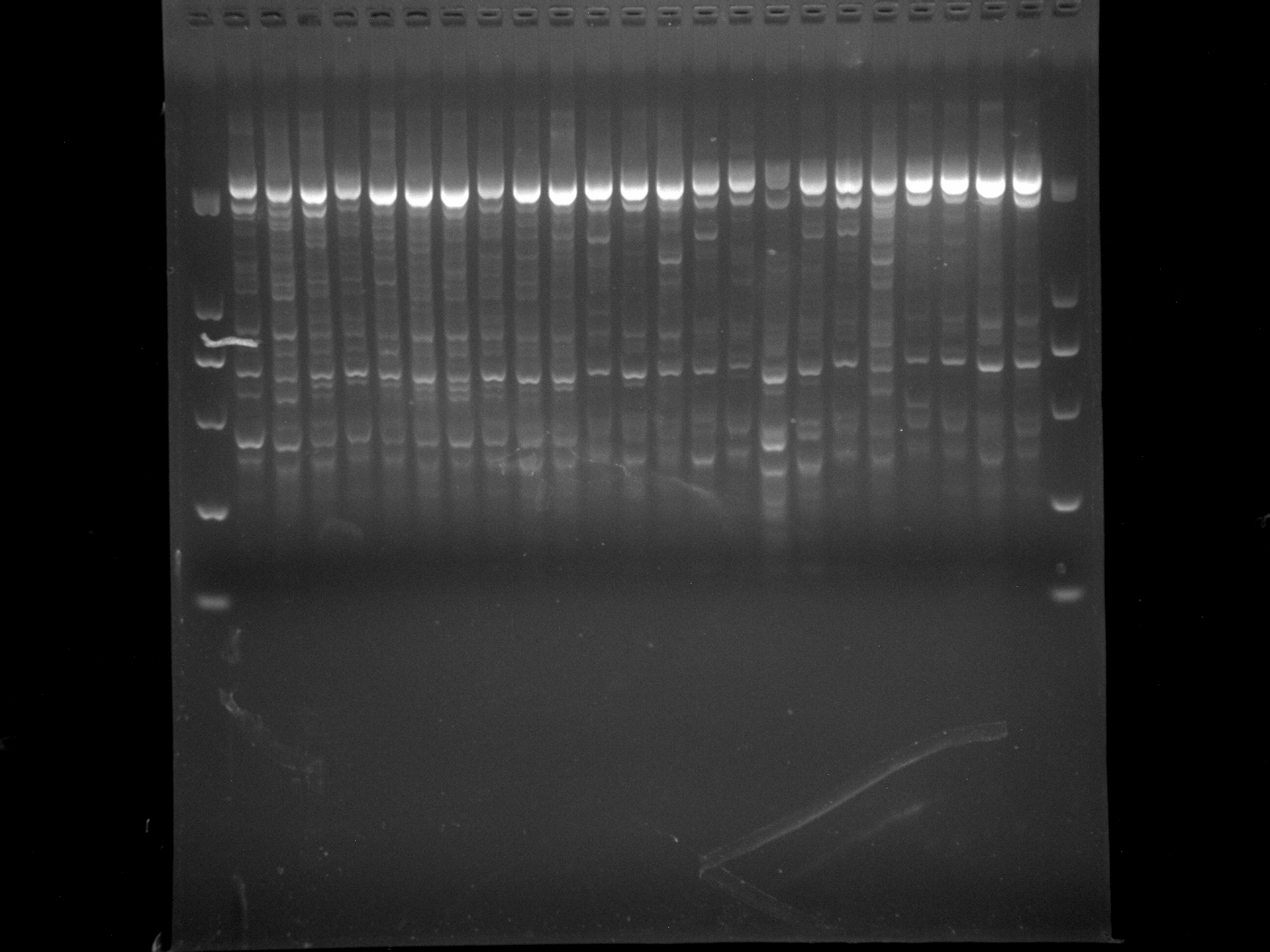

Supplement: Supplemental Information 71 — KNOX-3 amplification results for LS5-13, DGD1-13 samples. [file peerj-08-8498-s071.png]

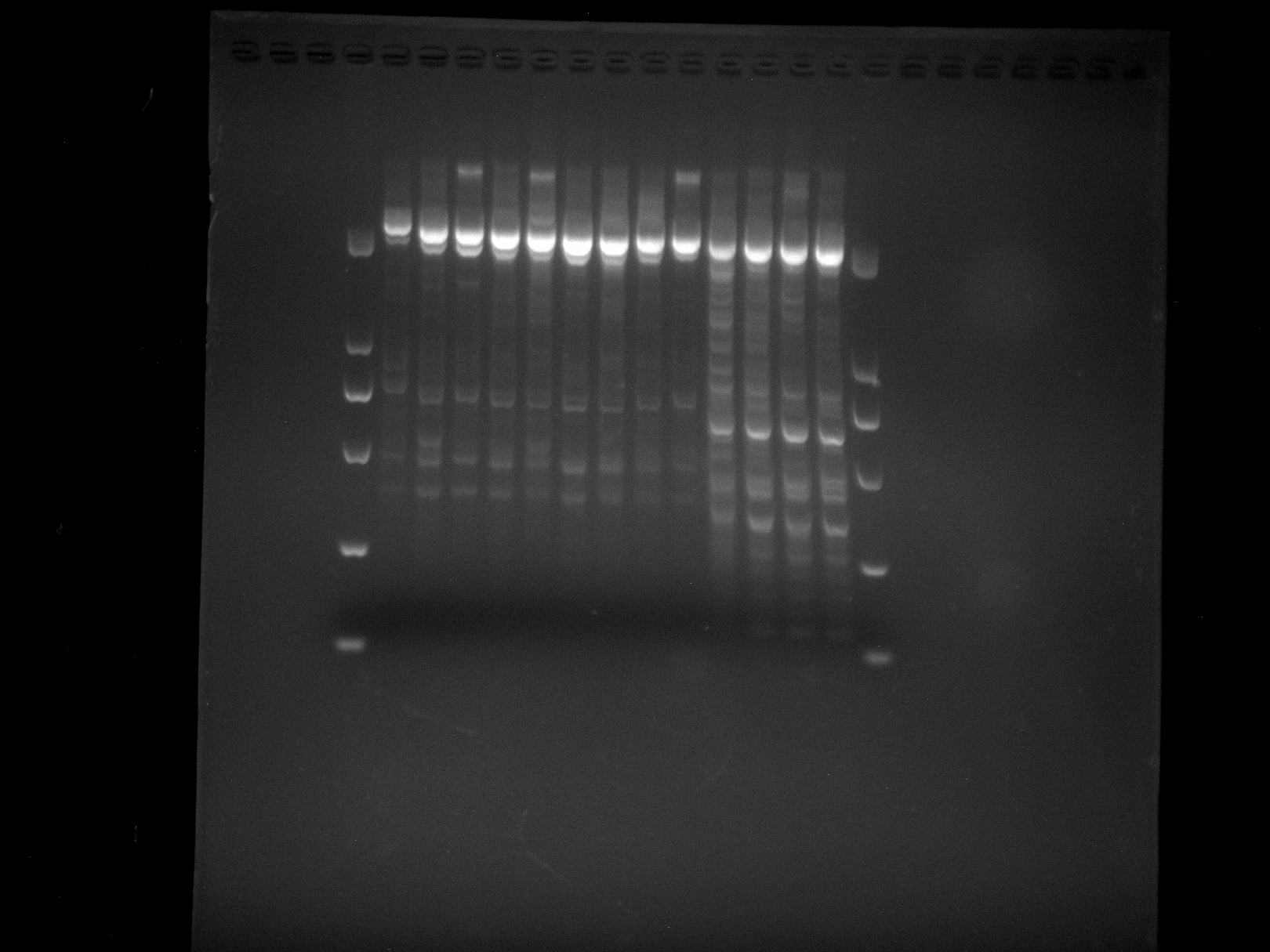

Supplement: Supplemental Information 72 — KNOX-3 amplification results for DGD14-26 samples. [file peerj-08-8498-s072.png]

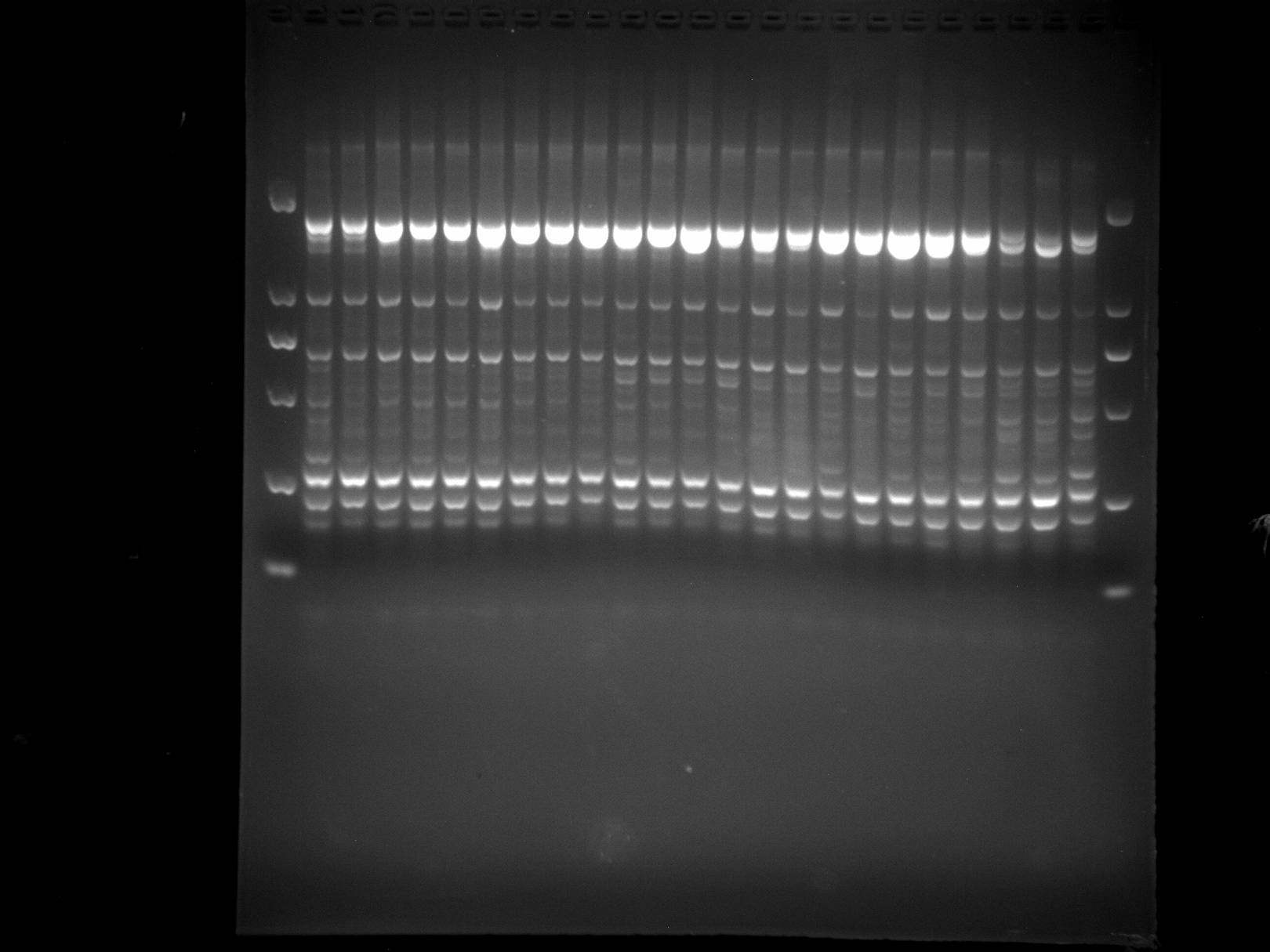

Supplement: Supplemental Information 73 — Amplification results of MADS-1 on LGD1-8, DRS9-23 samples. [file peerj-08-8498-s073.png]

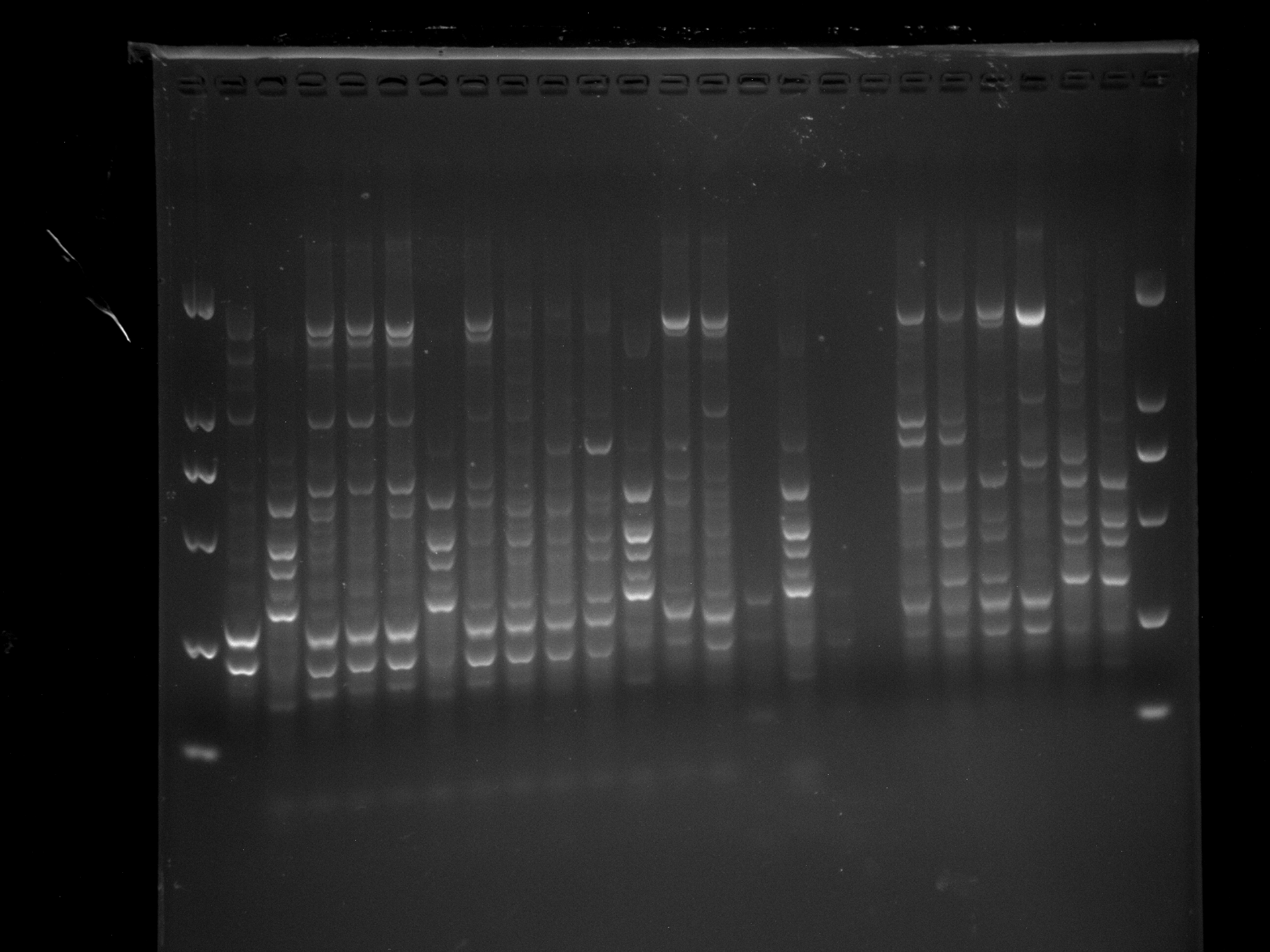

Supplement: Supplemental Information 74 — Amplification results of MADS-1 on DRS24-28, NJD1-18 samples. [file peerj-08-8498-s074.png]

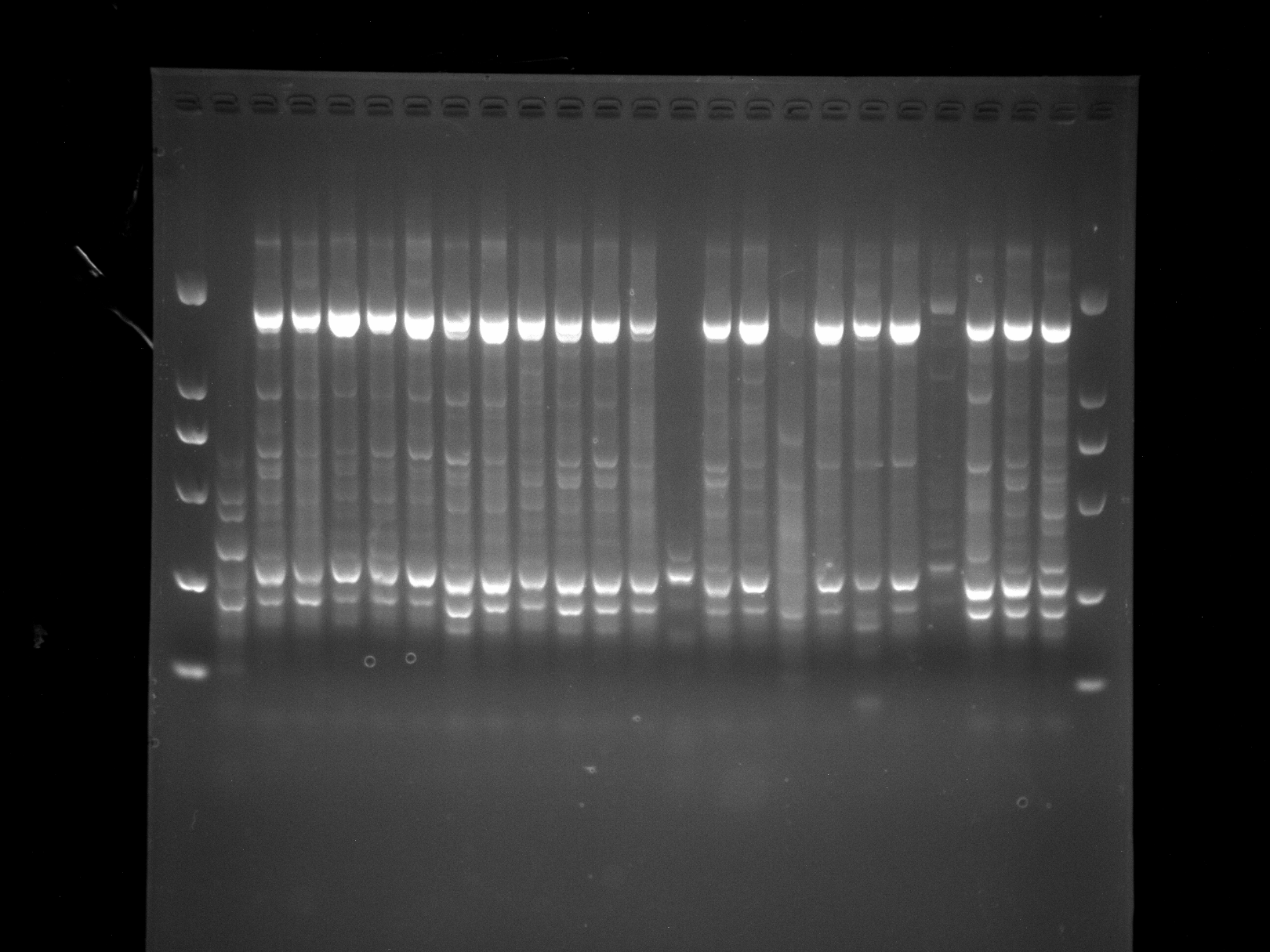

Supplement: Supplemental Information 75 — Amplification results of MADS-1 on NJD19-NJD33, PTD1, LSD1, LS1-4 samples. [file peerj-08-8498-s075.png]

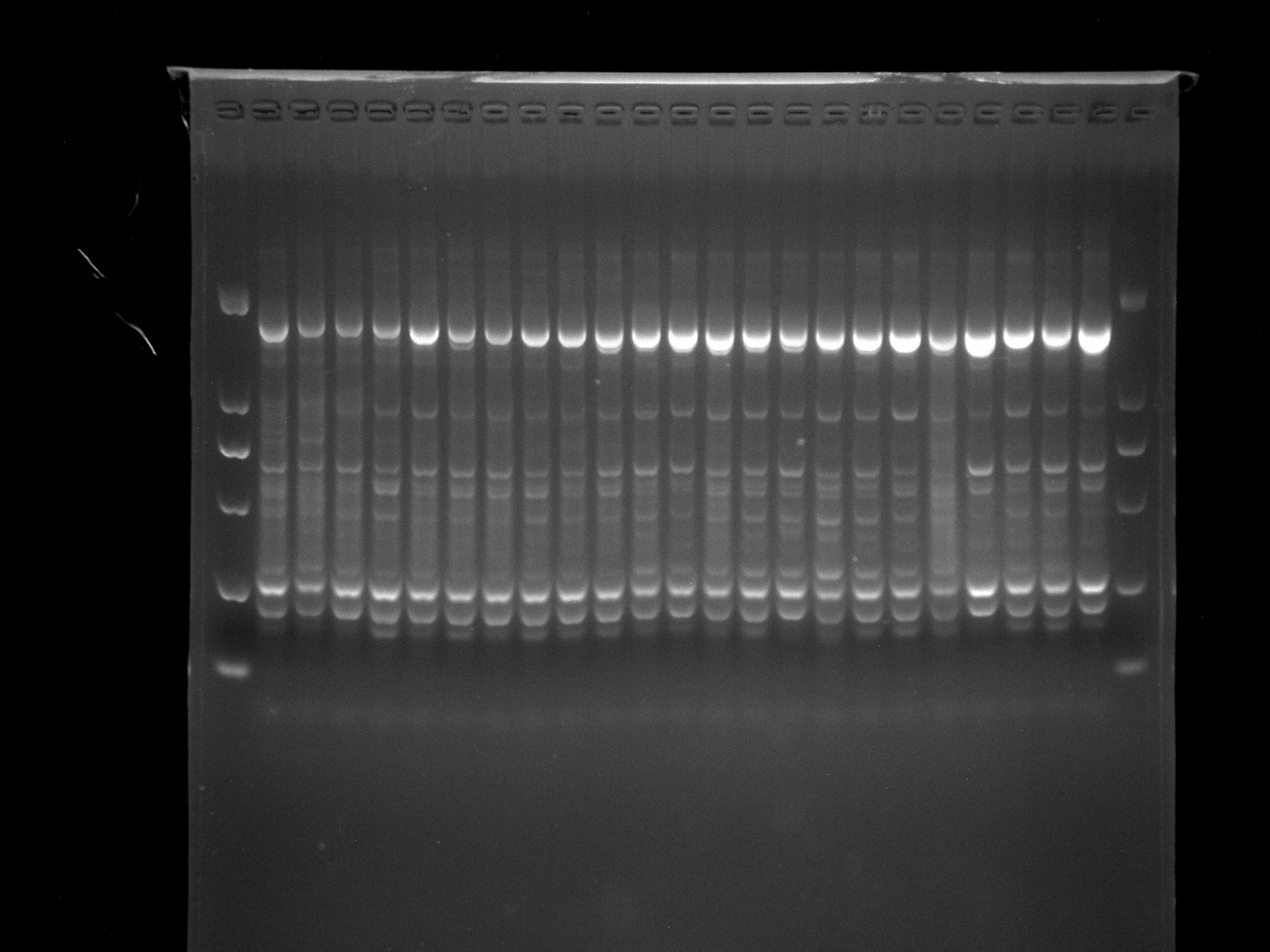

Supplement: Supplemental Information 76 — Amplification results of MADS-1 on LS5-13, DGD1-13 samples. [file peerj-08-8498-s076.png]

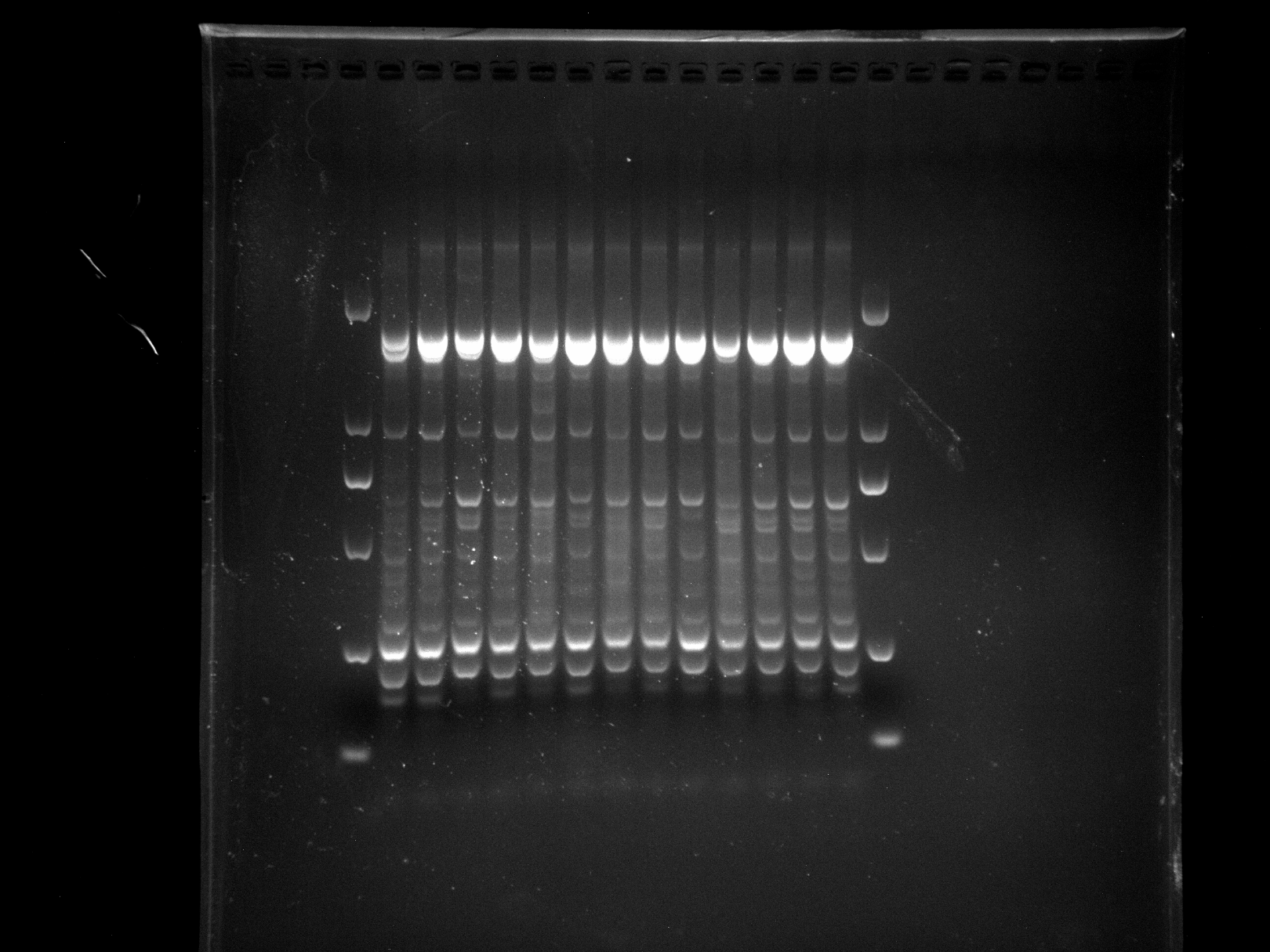

Supplement: Supplemental Information 77 — Amplification results of MADS-1 on DGD14-26 samples. [file peerj-08-8498-s077.png]

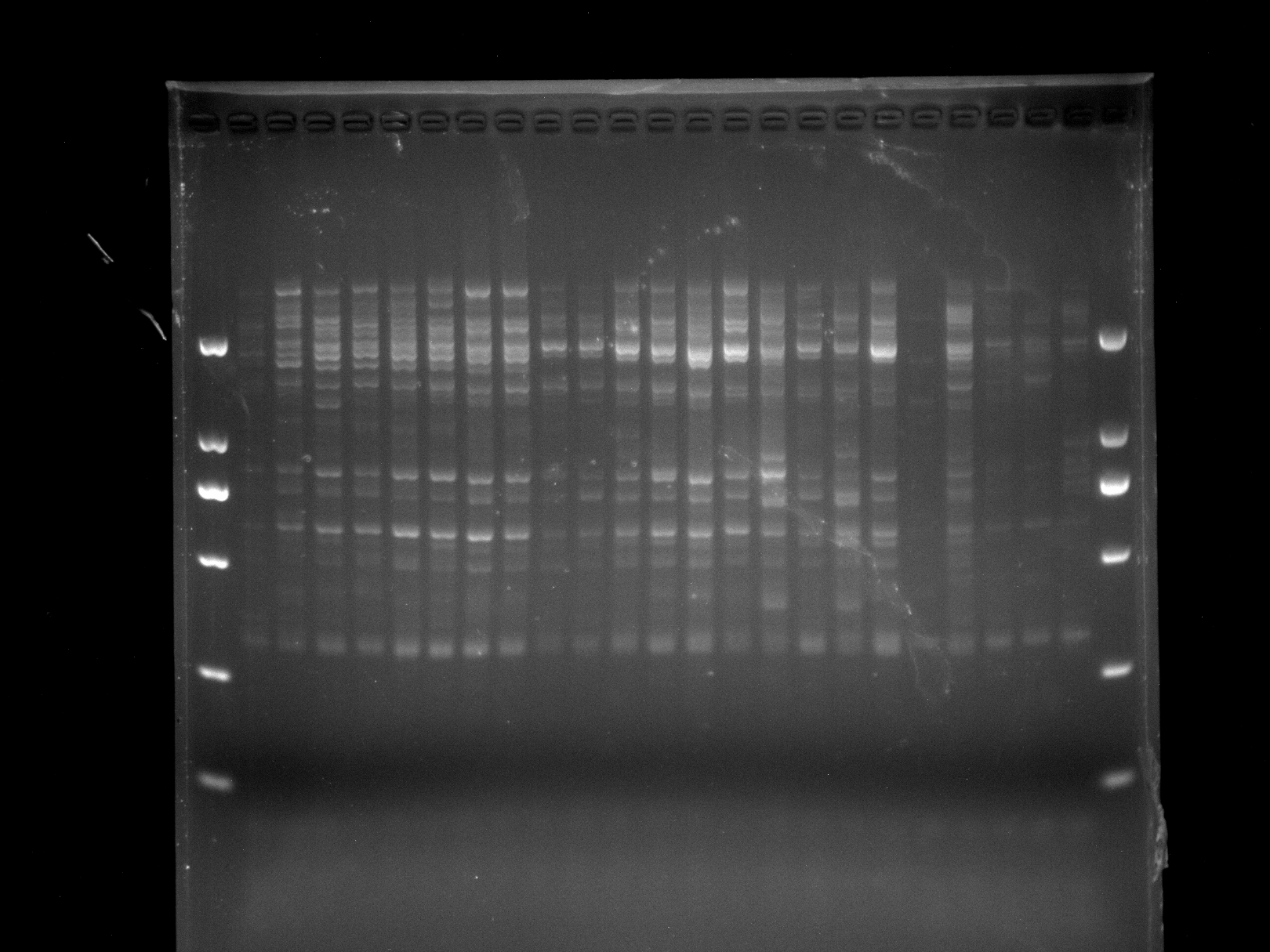

Supplement: Supplemental Information 78 — Amplification results of MADS-4 on LGD1-8 and DRS9-23 samples. [file peerj-08-8498-s078.png]

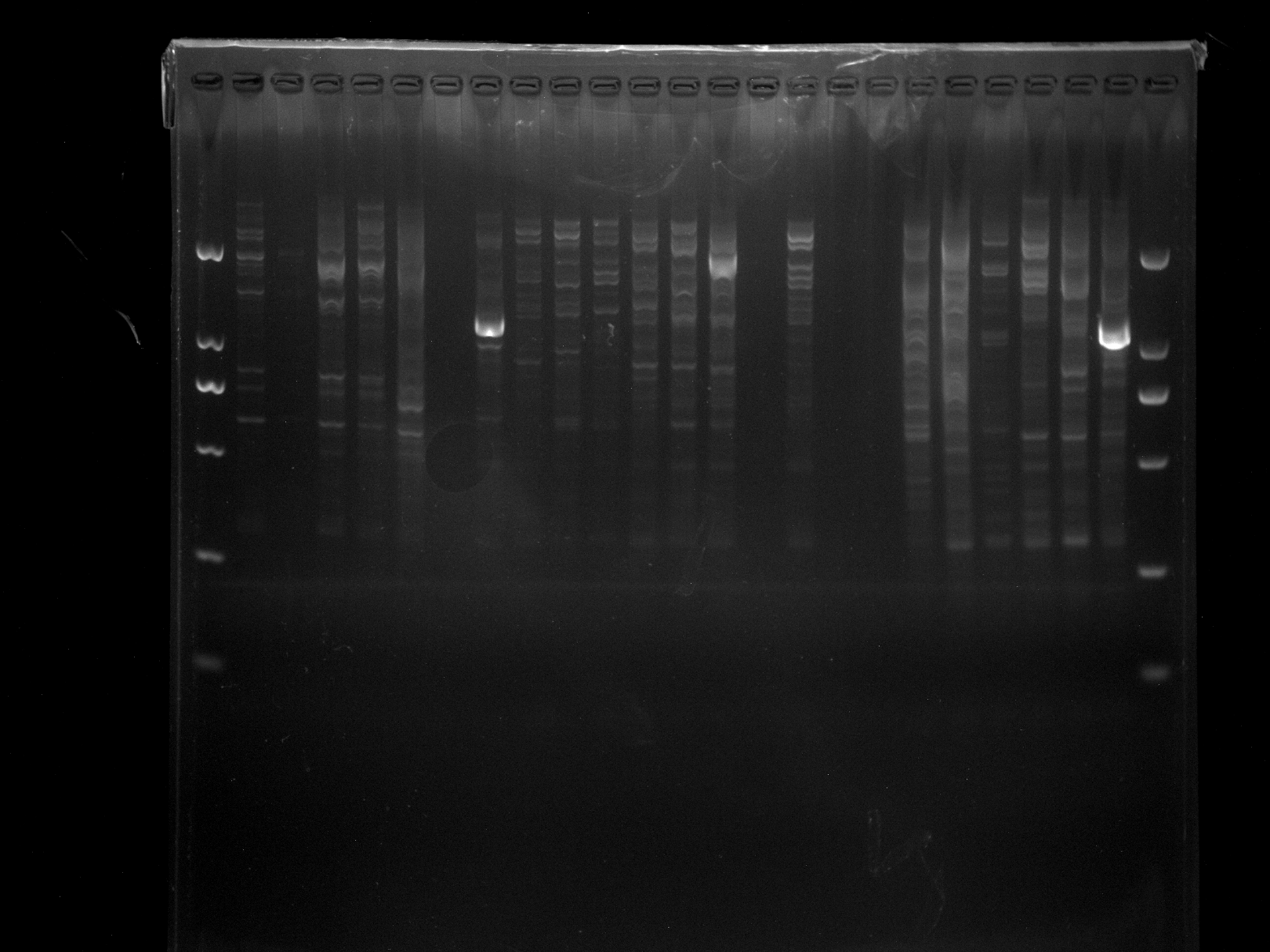

Supplement: Supplemental Information 79 — Amplification results of MADS-4 on DRS24-28, NJD1-18 samples. [file peerj-08-8498-s079.png]

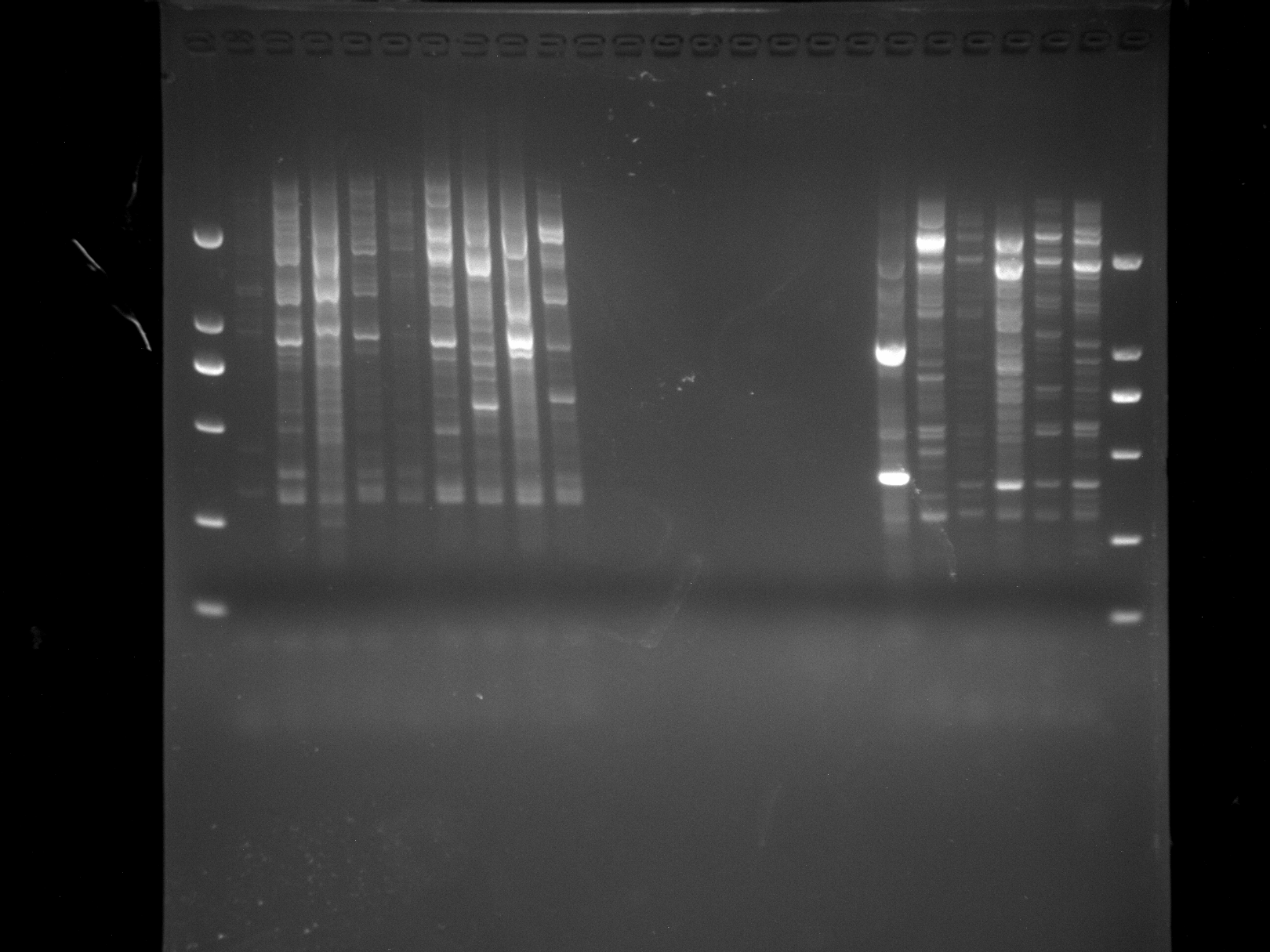

Supplement: Supplemental Information 80 — Amplification results of MADS-4 on NJD19-33, PUD1, LSD1, LS1-4 samples. [file peerj-08-8498-s080.png]

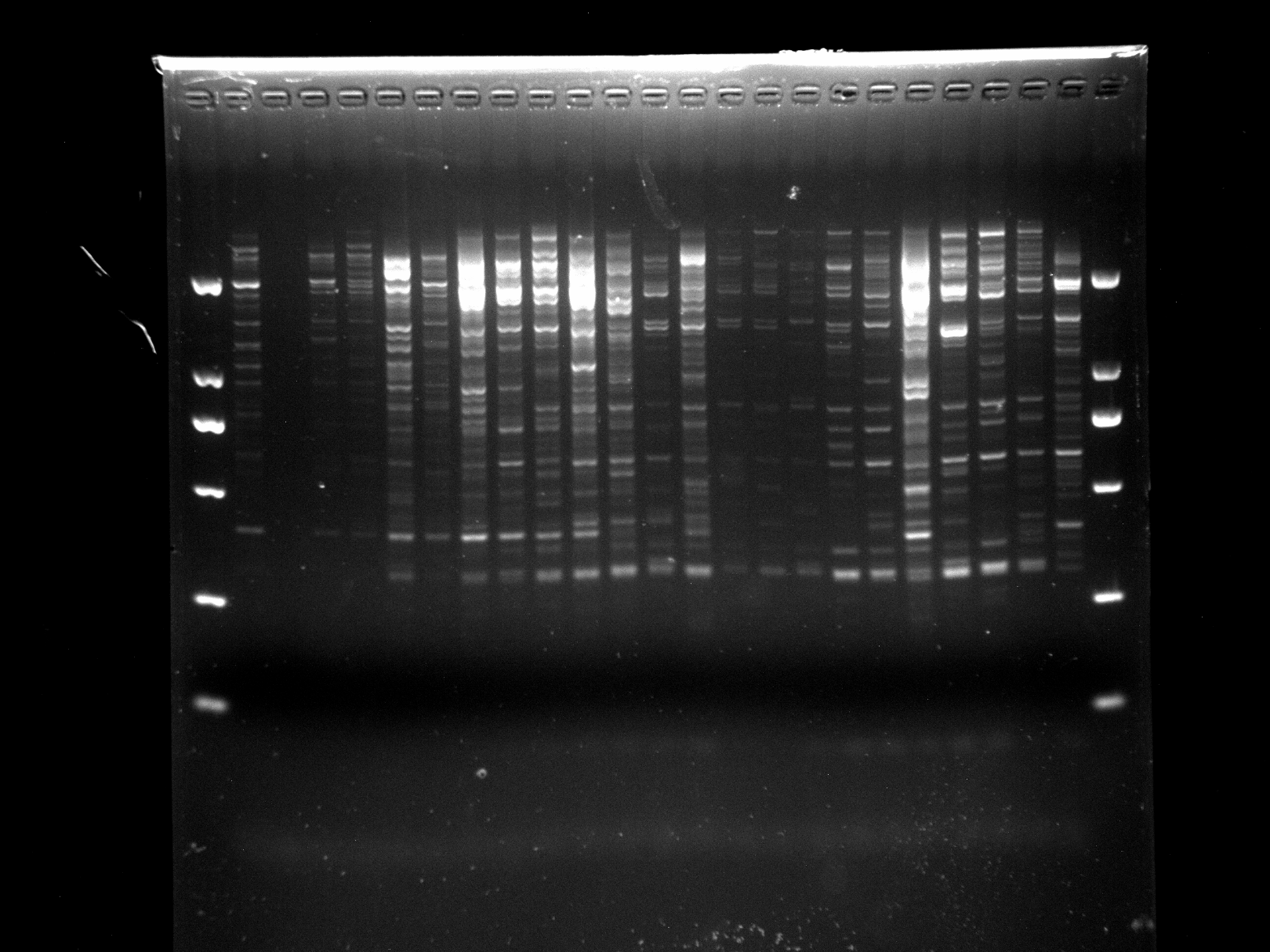

Supplement: Supplemental Information 81 — Amplification results of MADS-4 on LS5-13, DGD1-13 samples. [file peerj-08-8498-s081.png]

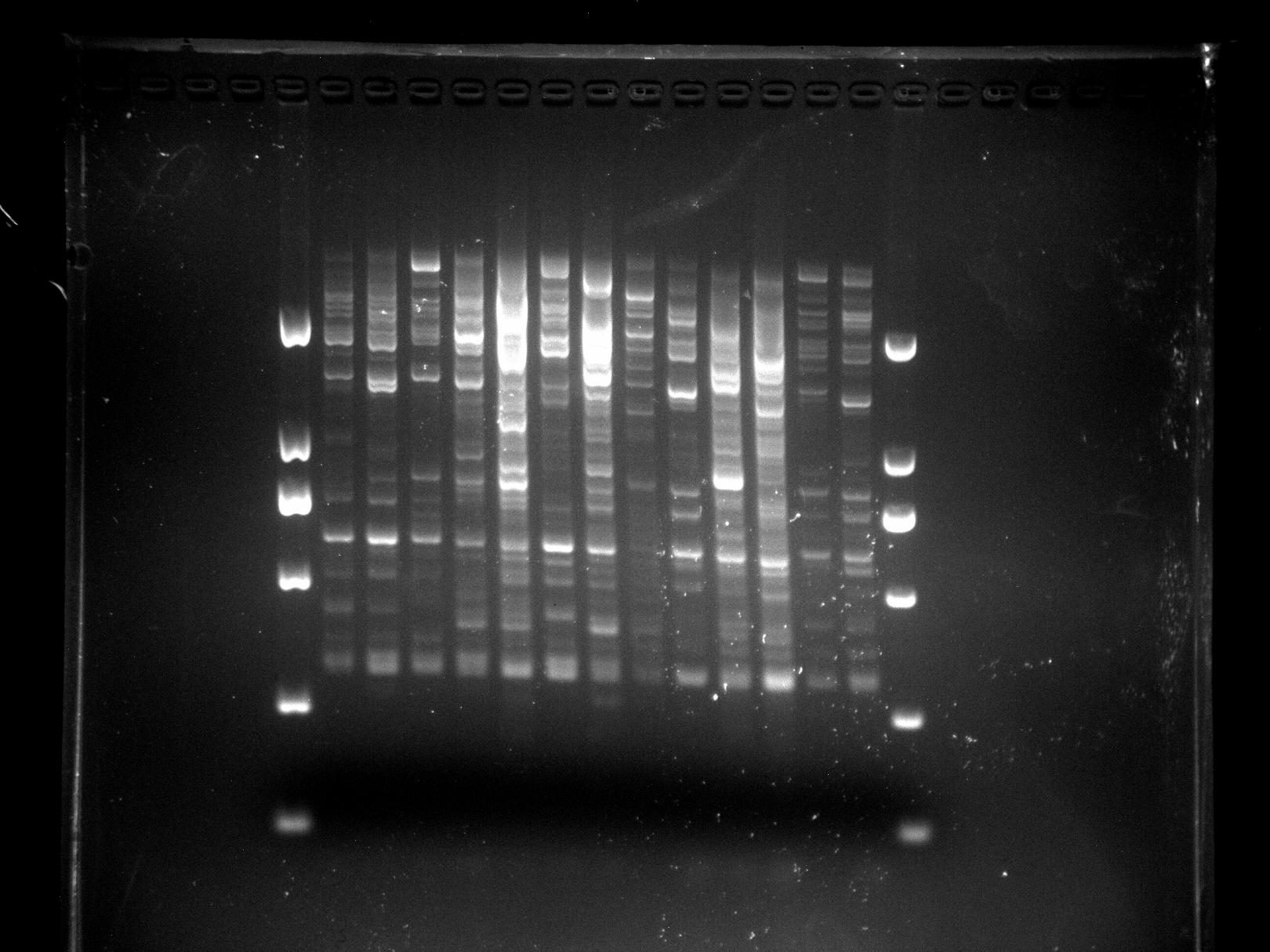

Supplement: Supplemental Information 82 — Amplification results of MADS-4 on DGD14-26 samples. [file peerj-08-8498-s082.png]

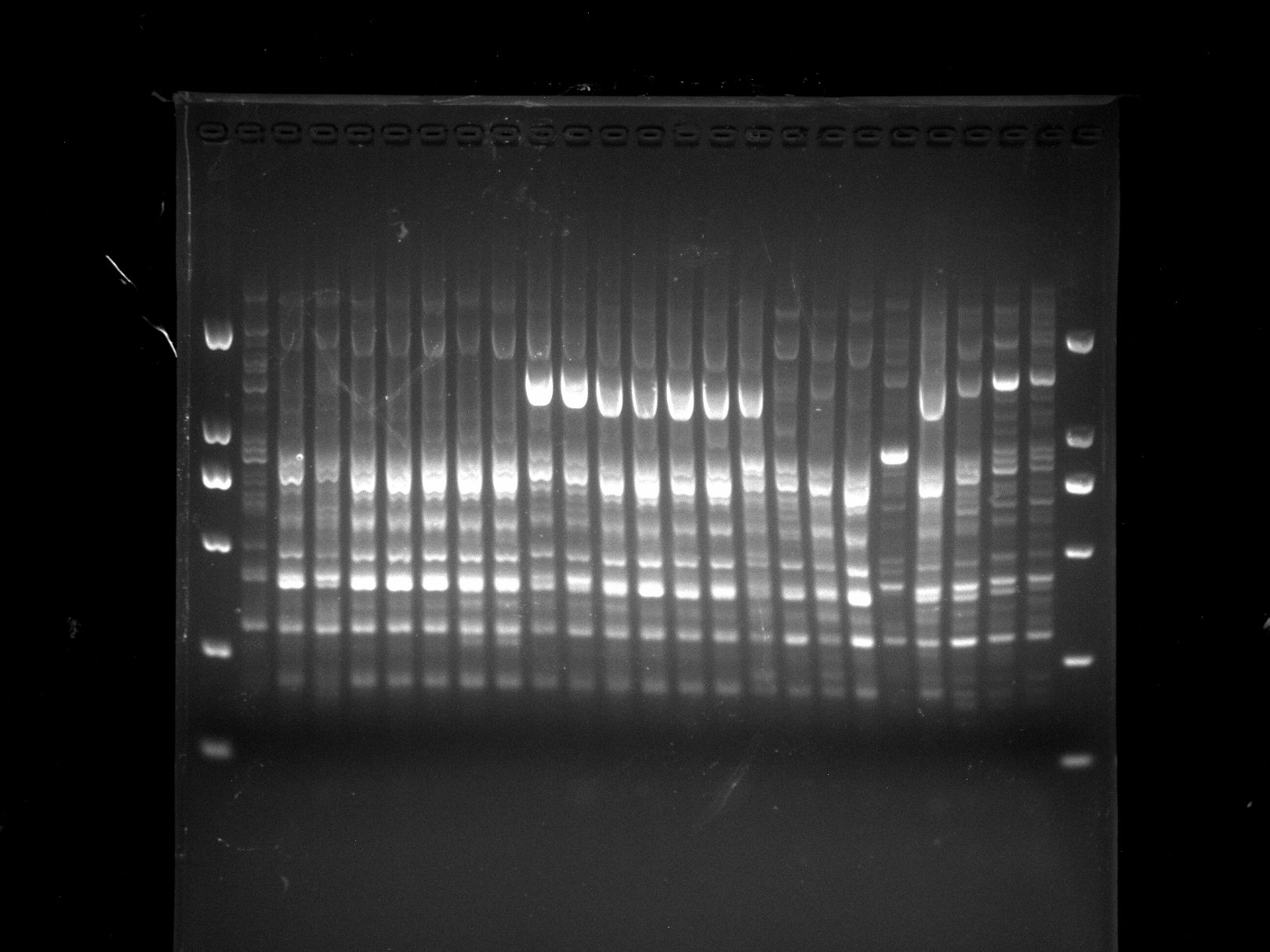

Supplement: Supplemental Information 83 — ABP1-1 amplification results for LGD1-8, DRS9-23 samples. [file peerj-08-8498-s083.png]

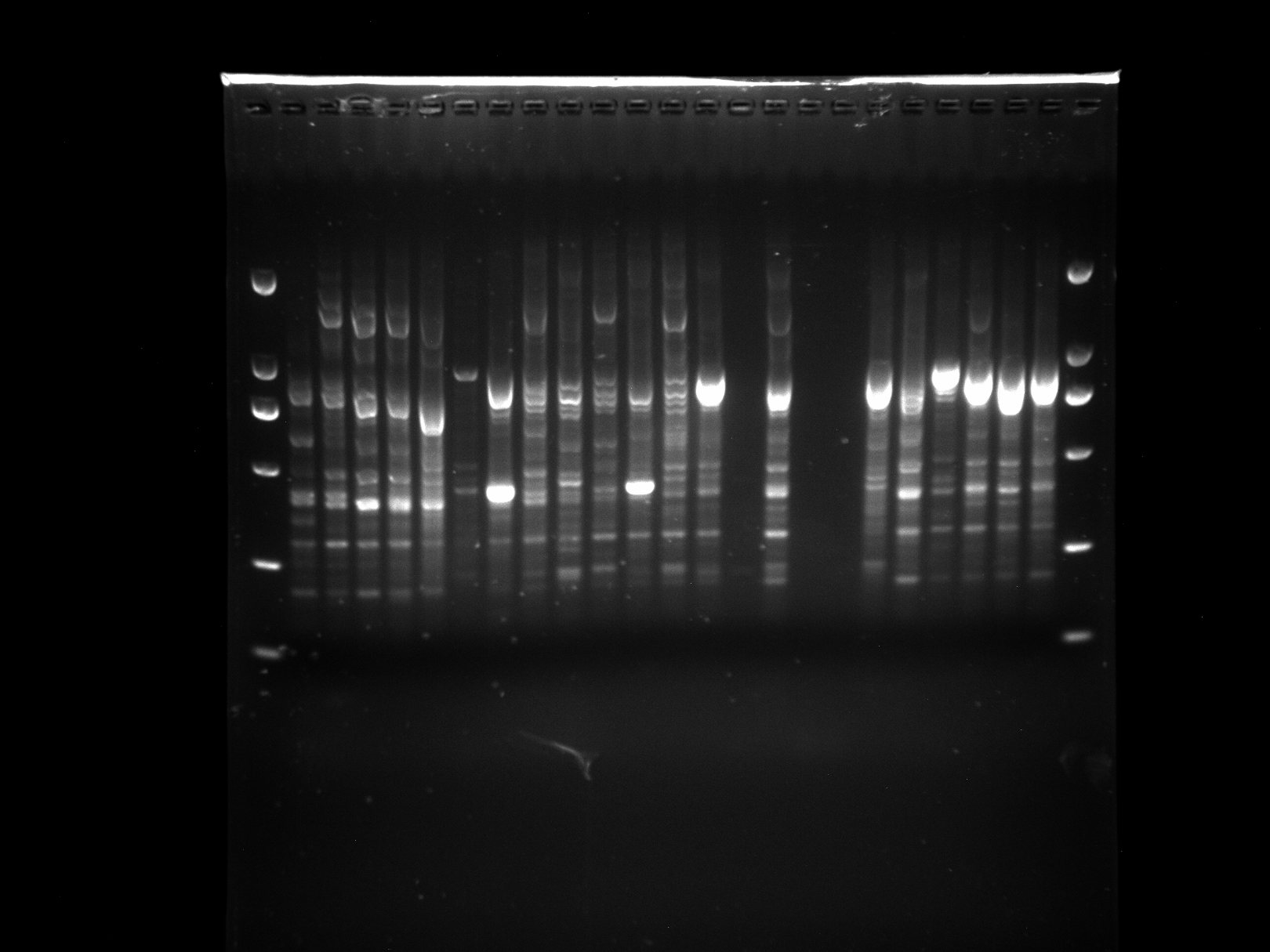

Supplement: Supplemental Information 84 — ABP1-1 amplification results for DRS24-28, NJD1-18 samples. [file peerj-08-8498-s084.png]

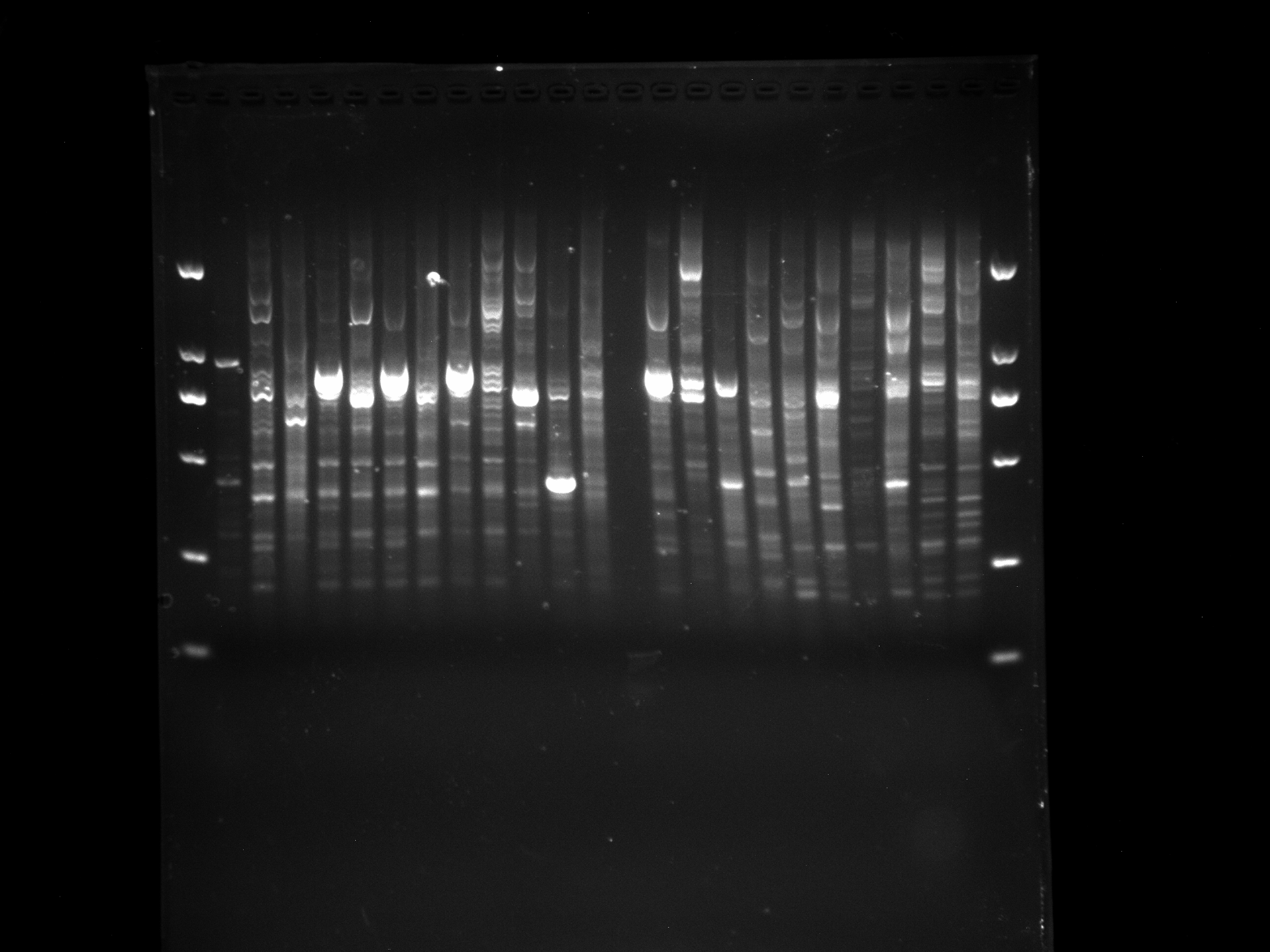

Supplement: Supplemental Information 85 — ABP1-1 amplification results for NJD19-33, LS1-4 samples. [file peerj-08-8498-s085.png]

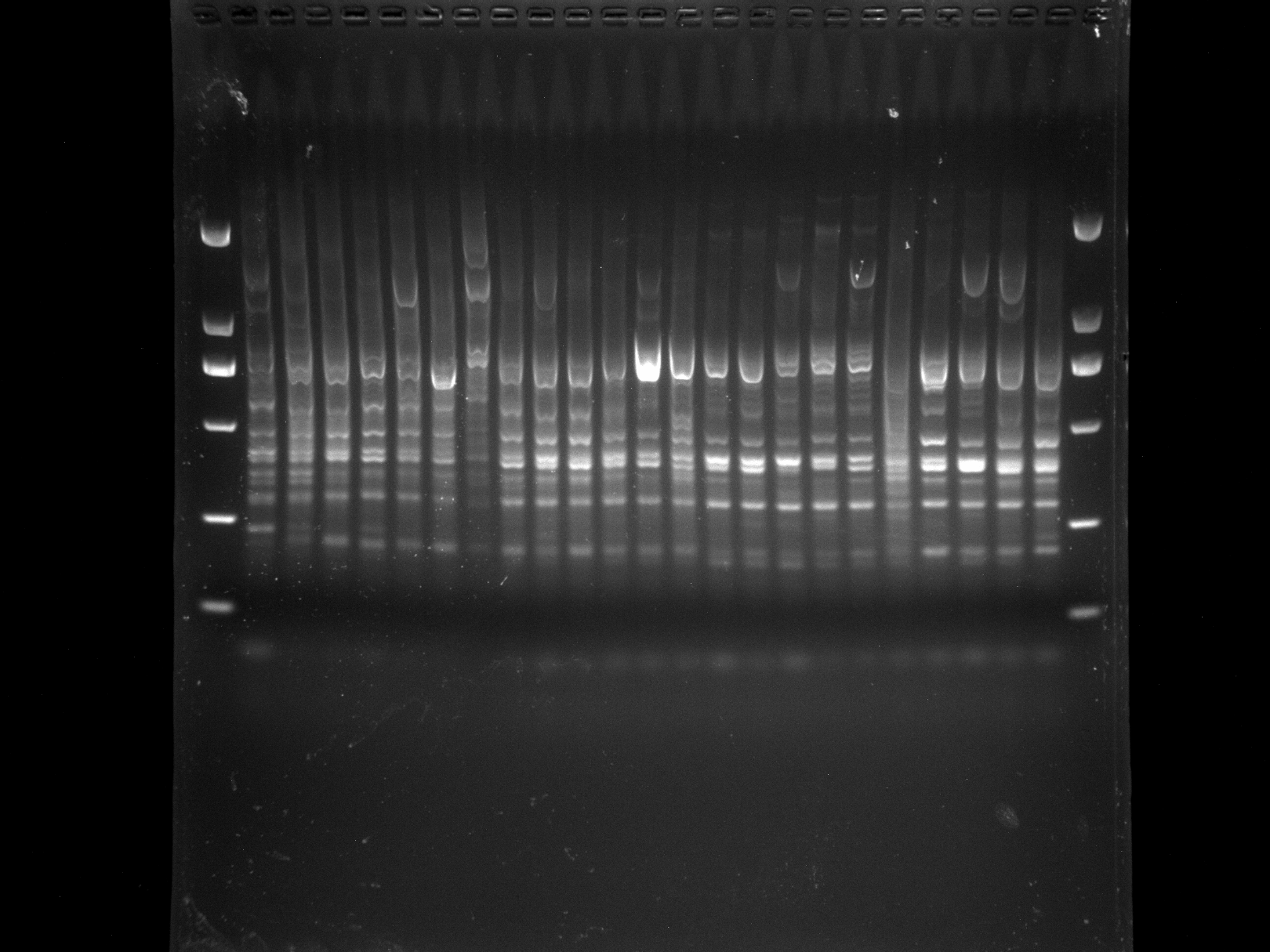

Supplement: Supplemental Information 86 — ABP1-1 amplification results for LS5-13, DGD1-13 samples. [file peerj-08-8498-s086.png]

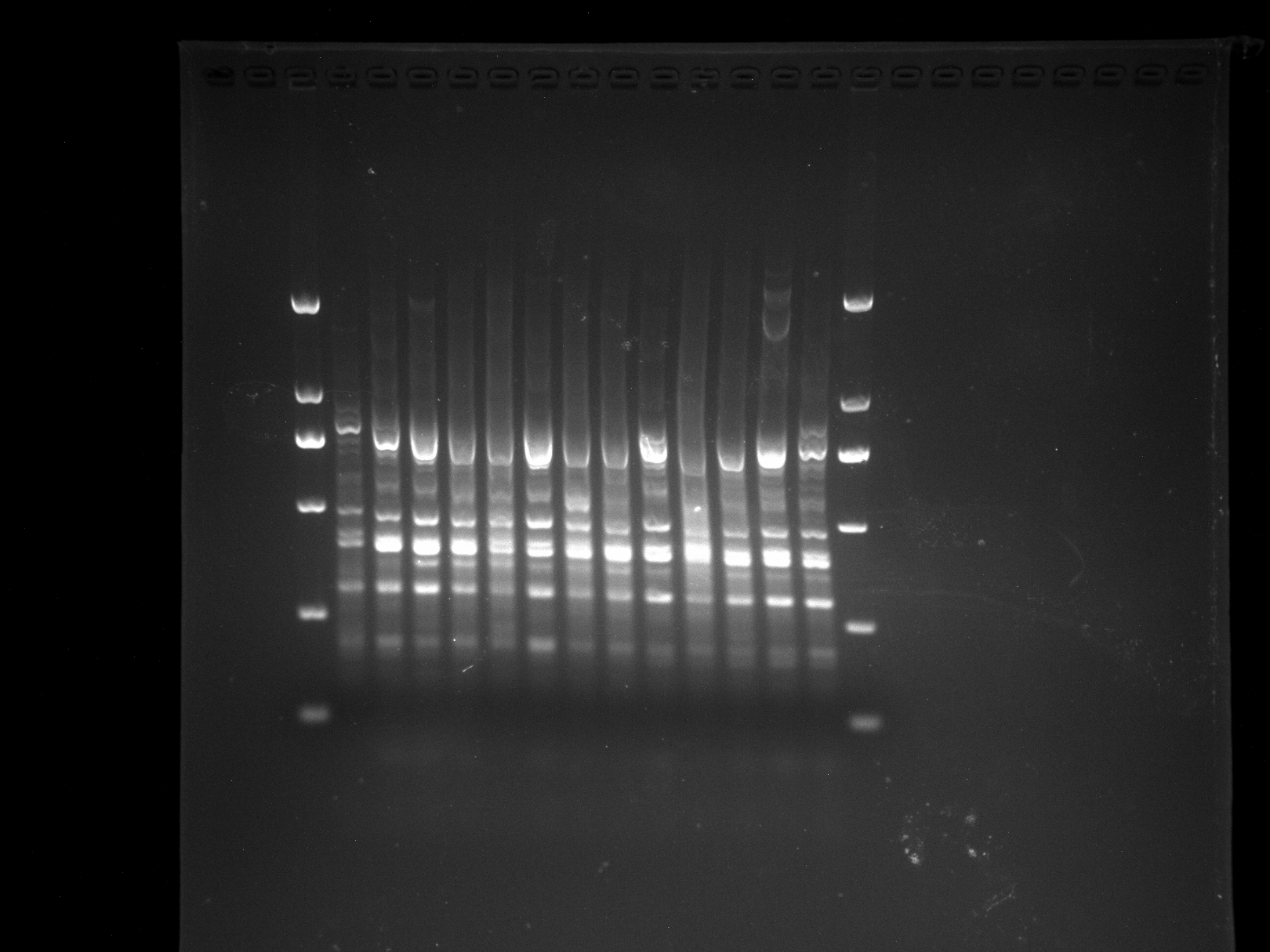

Supplement: Supplemental Information 87 — ABP1-1 amplification results for DGD14-26 samples. [file peerj-08-8498-s087.png]

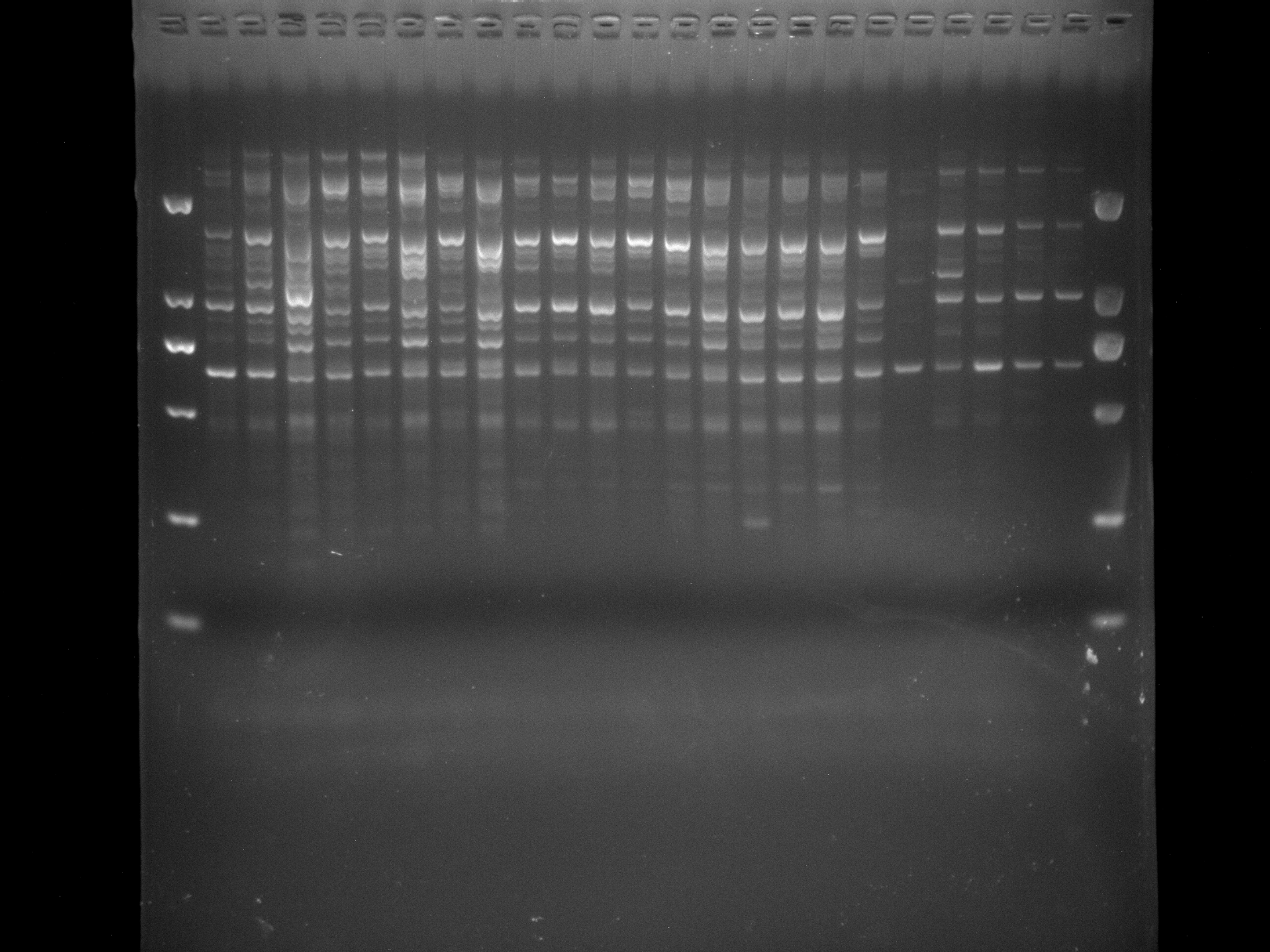

Supplement: Supplemental Information 88 — ABP1-3 amplification results for LGD1-8, DRS9-23 samples. [file peerj-08-8498-s088.png]

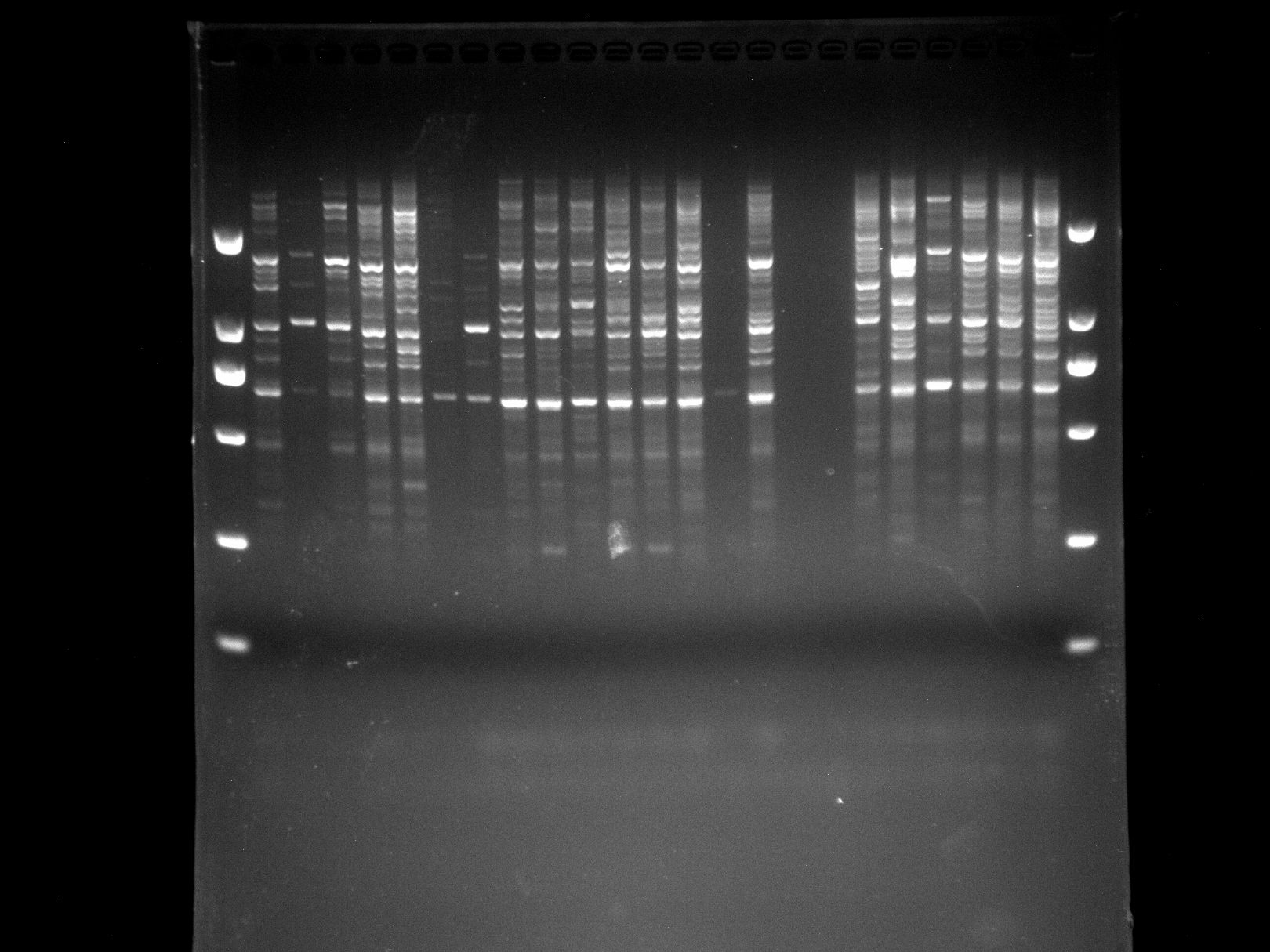

Supplement: Supplemental Information 89 — ABP1-3 amplification results of DRS24-28, NJD1-18 samples. [file peerj-08-8498-s089.png]

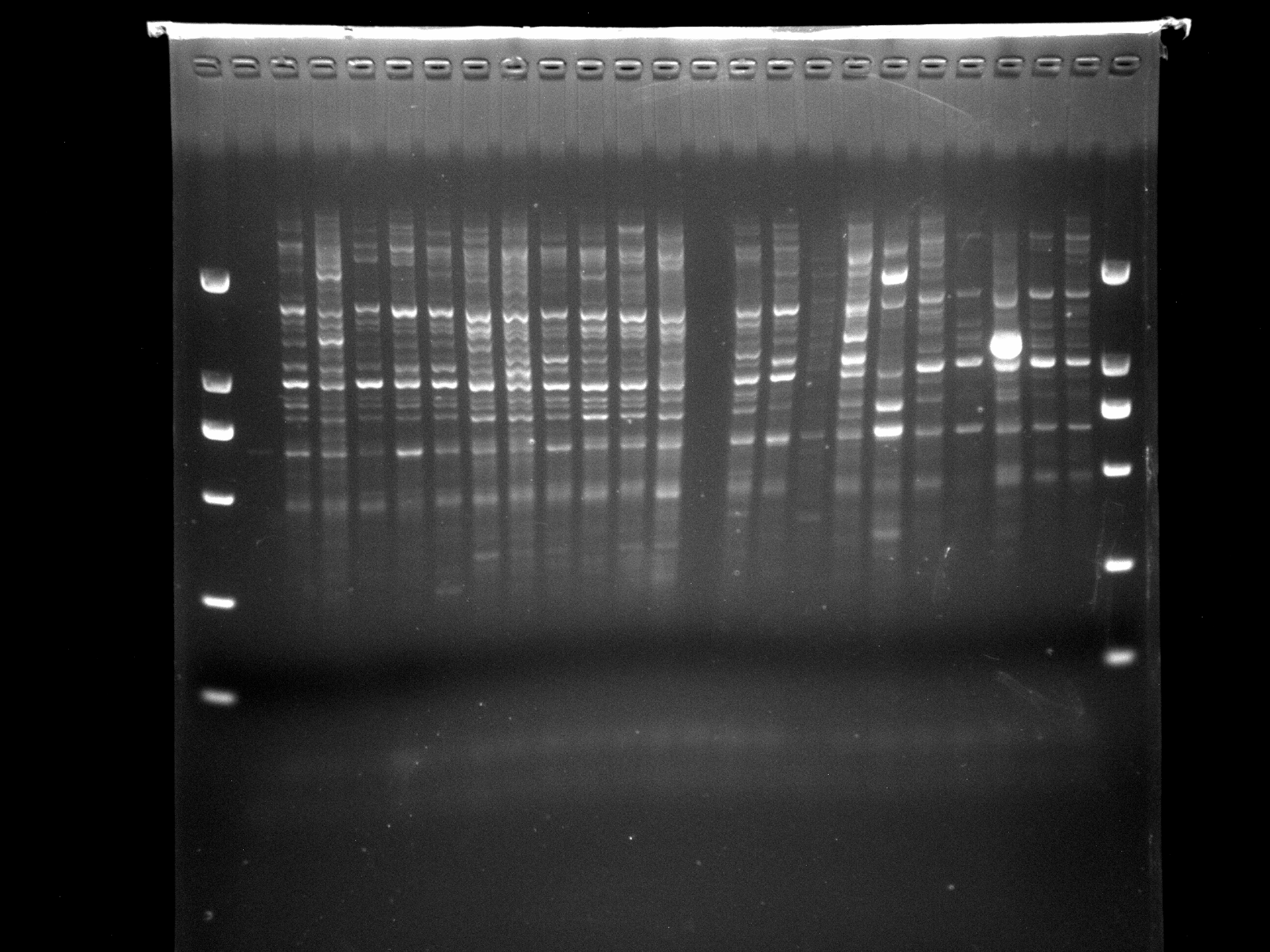

Supplement: Supplemental Information 90 — ABP1-3 amplification results for NJD19-33, PTD1, LSD1, LS1-4 samples. [file peerj-08-8498-s090.png]

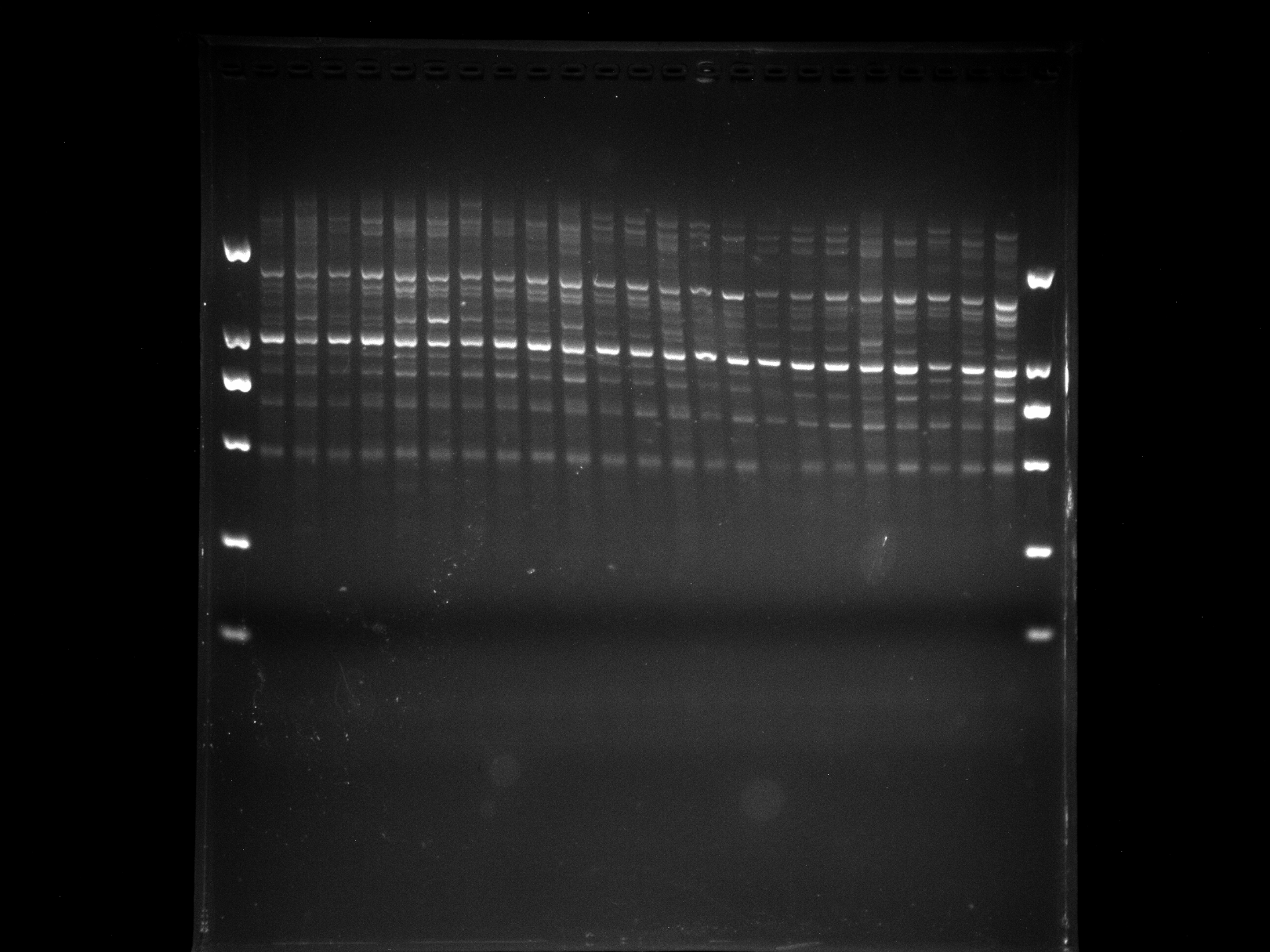

Supplement: Supplemental Information 91 — ABP1-3 amplification results for LS5-13, DGD1-13 samples. [file peerj-08-8498-s091.png]

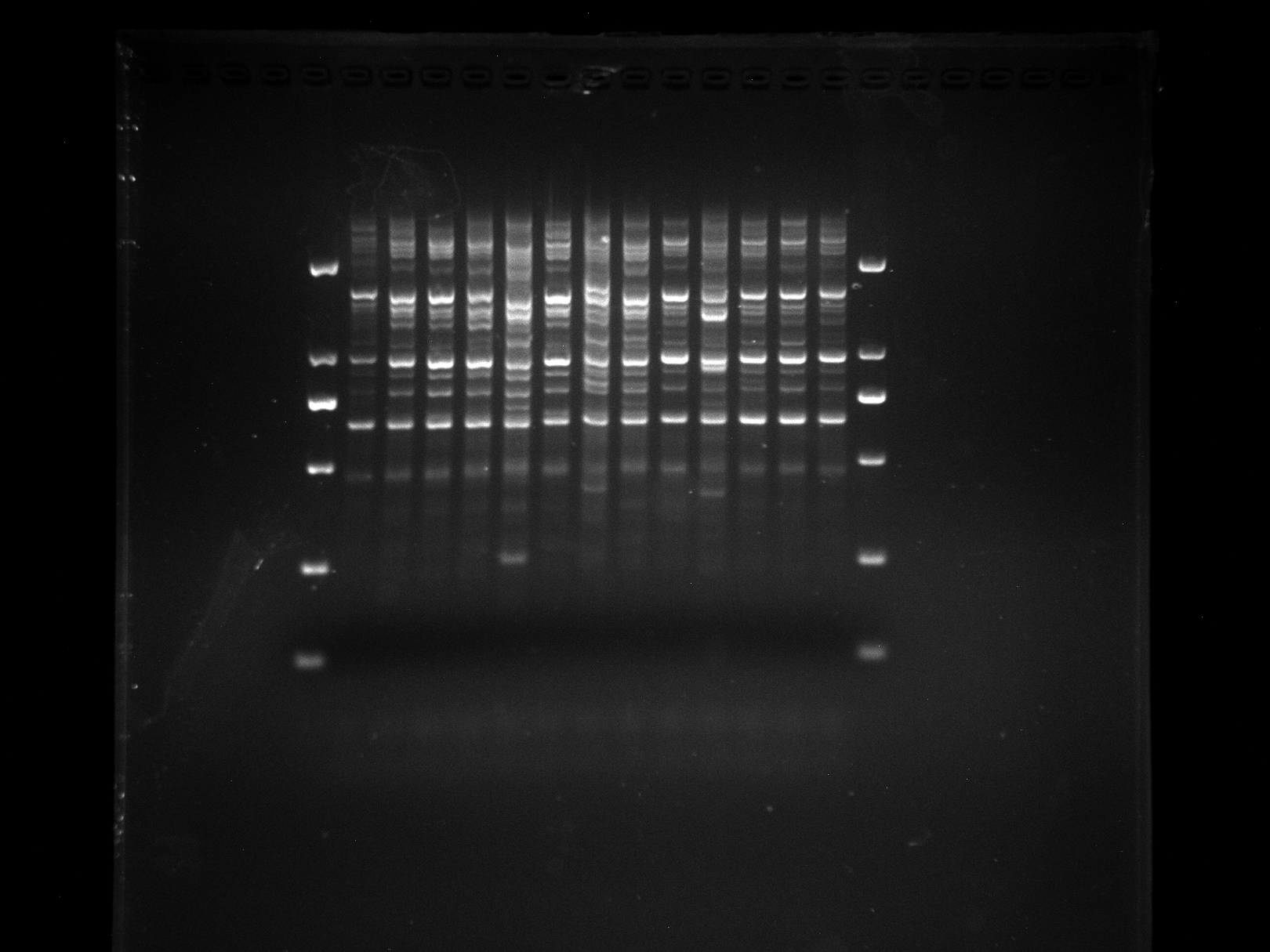

Supplement: Supplemental Information 92 — ABP1-3 amplification results for DGD14-26 samples. [file peerj-08-8498-s092.png]

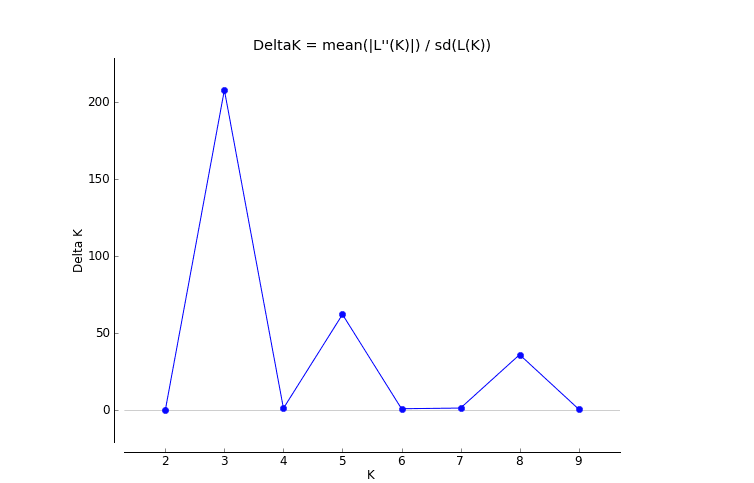

Supplement: Supplemental Information 93 — When K=3, the value of delat K is the largest and best fit three genetic groups. When K=5, the value of delat K is also large, so we analyze the cases of K=3 and K=5. [file peerj-08-8498-s093.png]

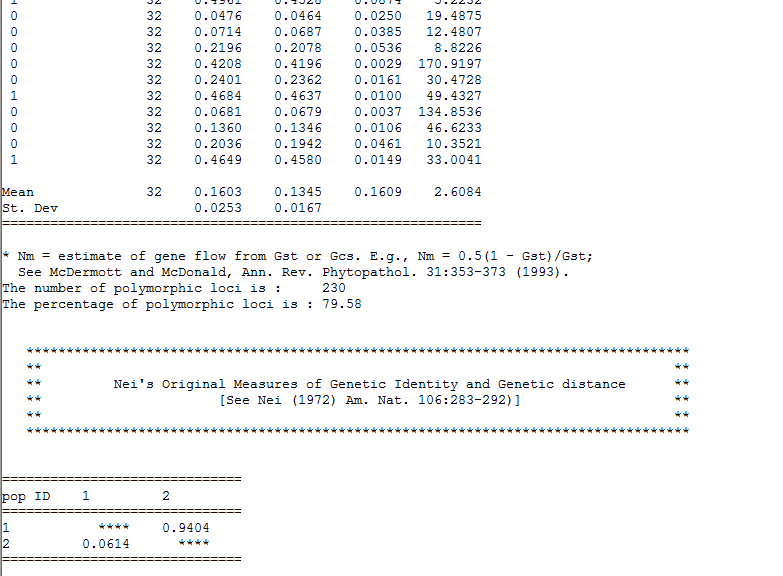

Supplement: Supplemental Information 94 — Nm represents the gene flow, and Gst represents the genetic differentiation coefficient. [file peerj-08-8498-s094.png]

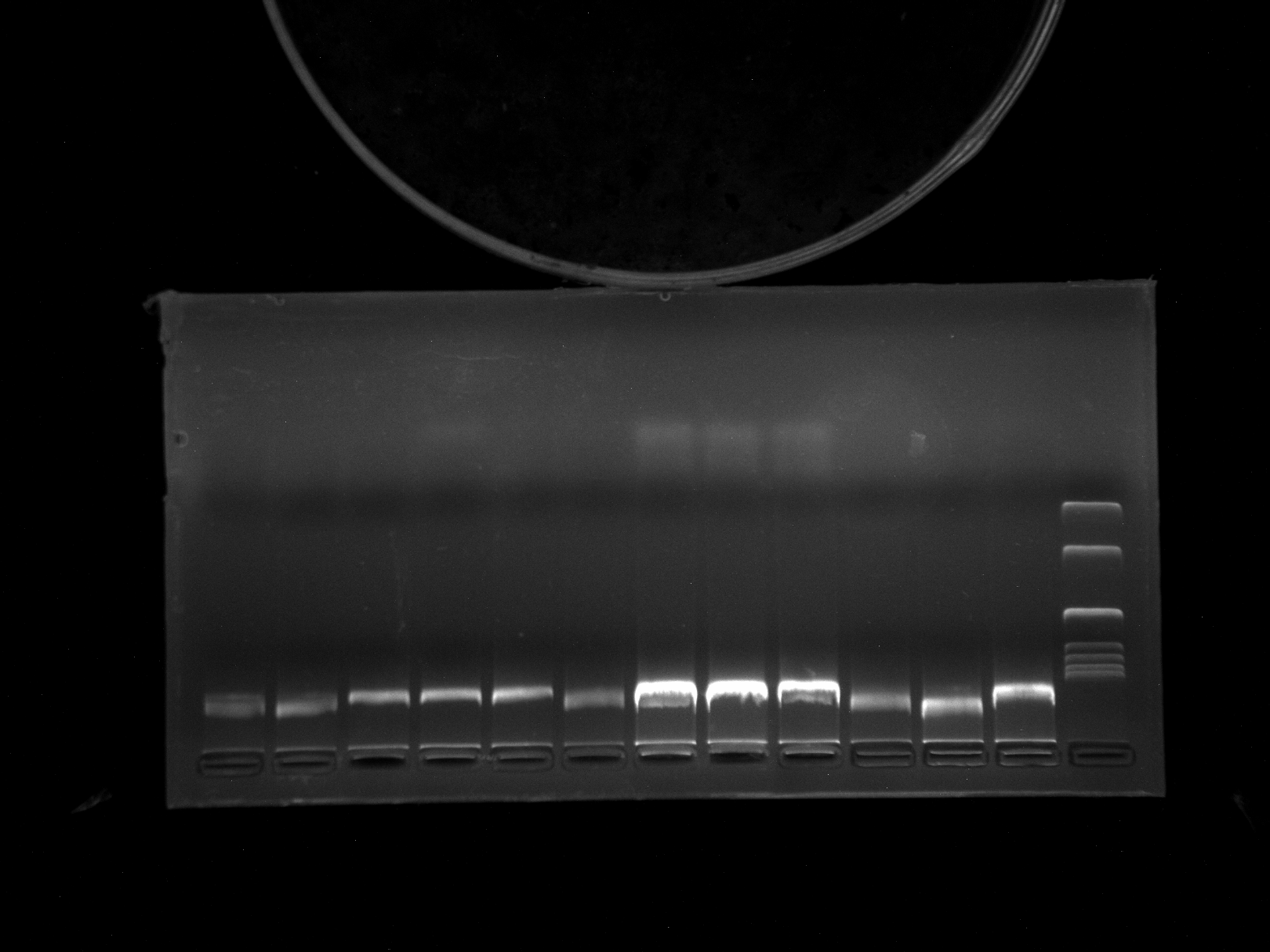

Supplement: Supplemental Information 95 — Two repetitions per sample. [file peerj-08-8498-s095.png]

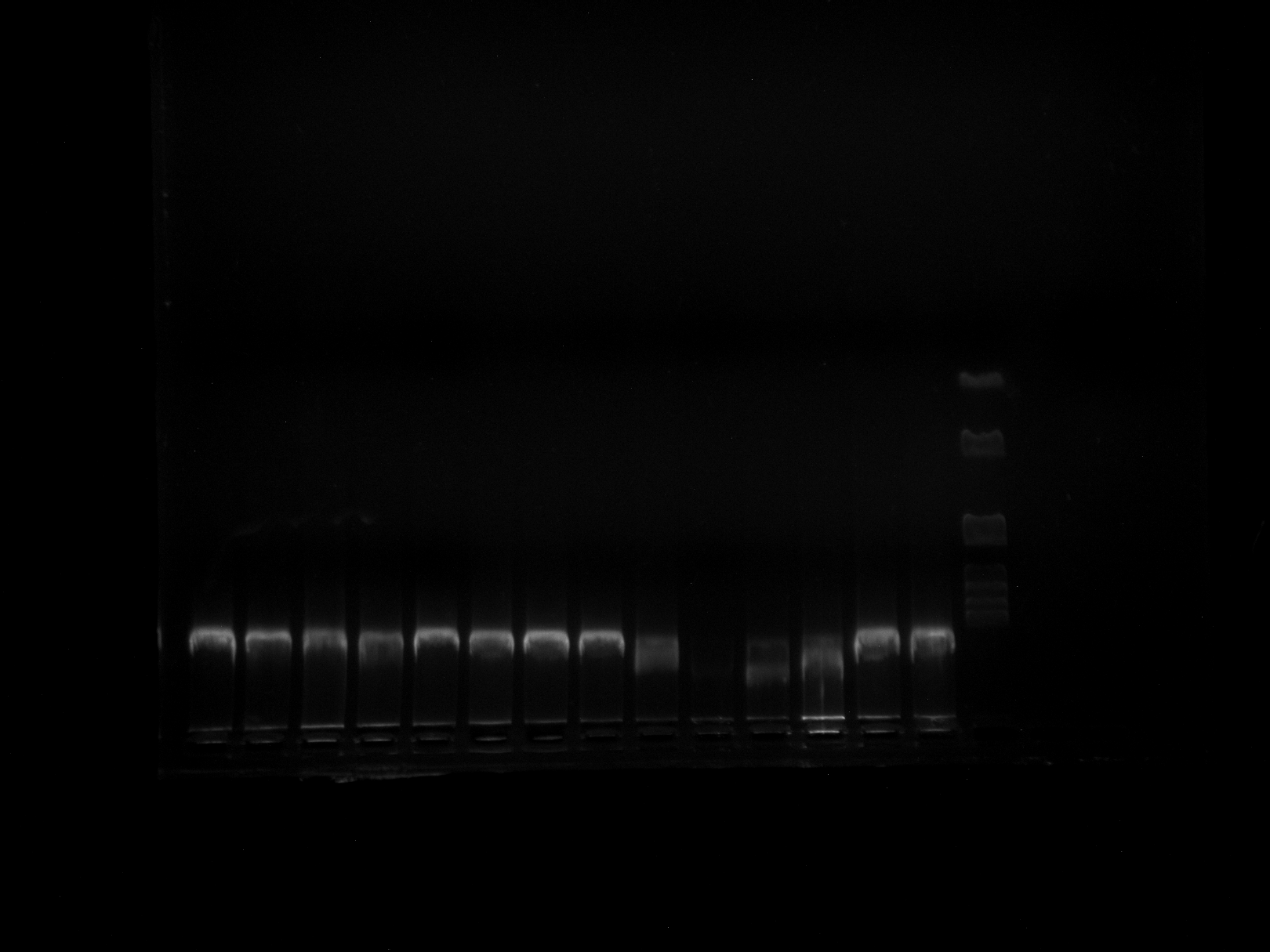

Supplement: Supplemental Information 96 — Two repetitions per sample. [file peerj-08-8498-s096.png]

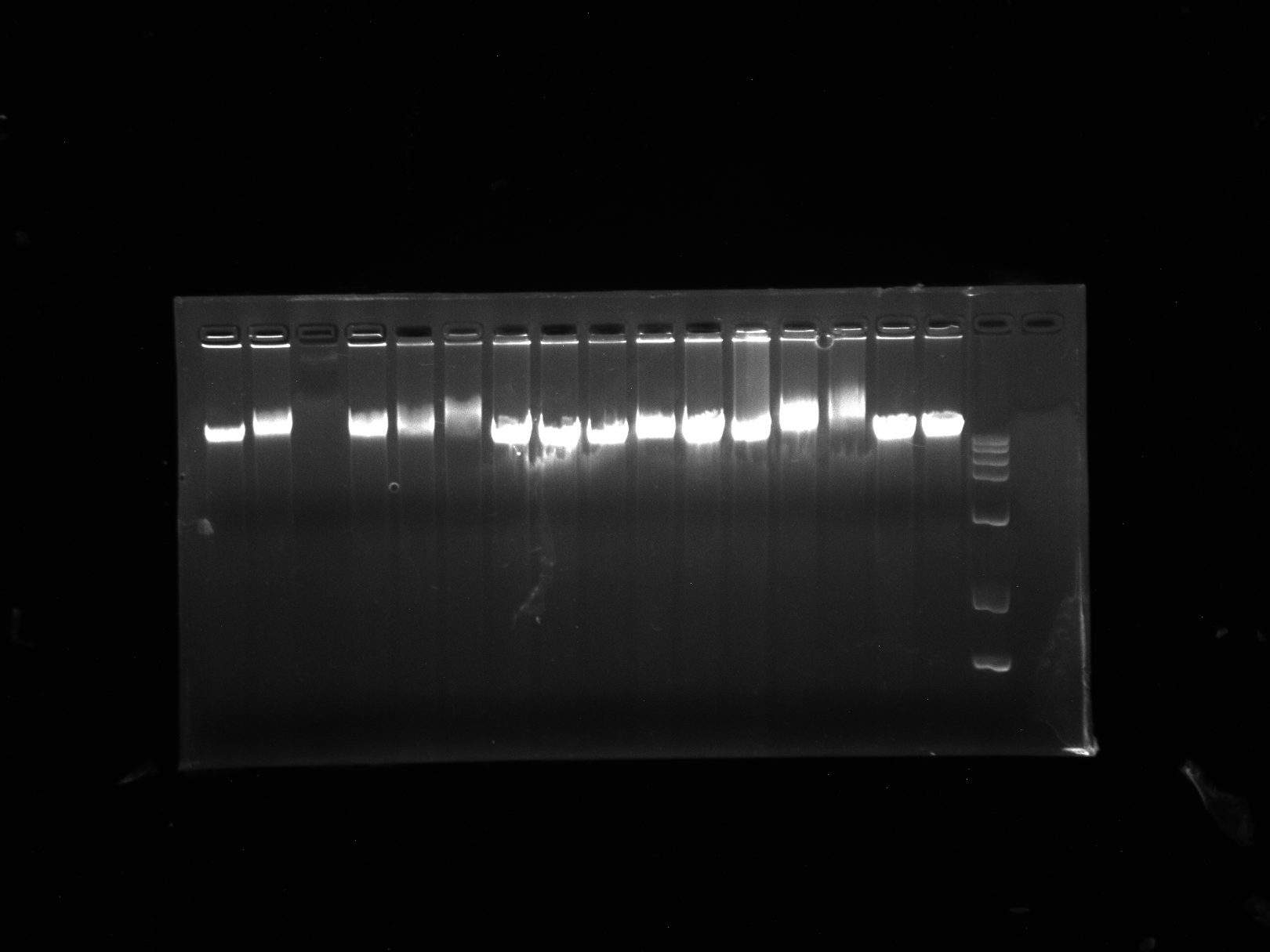

Supplement: Supplemental Information 97 — Two repetitions per sample. [file peerj-08-8498-s097.png]

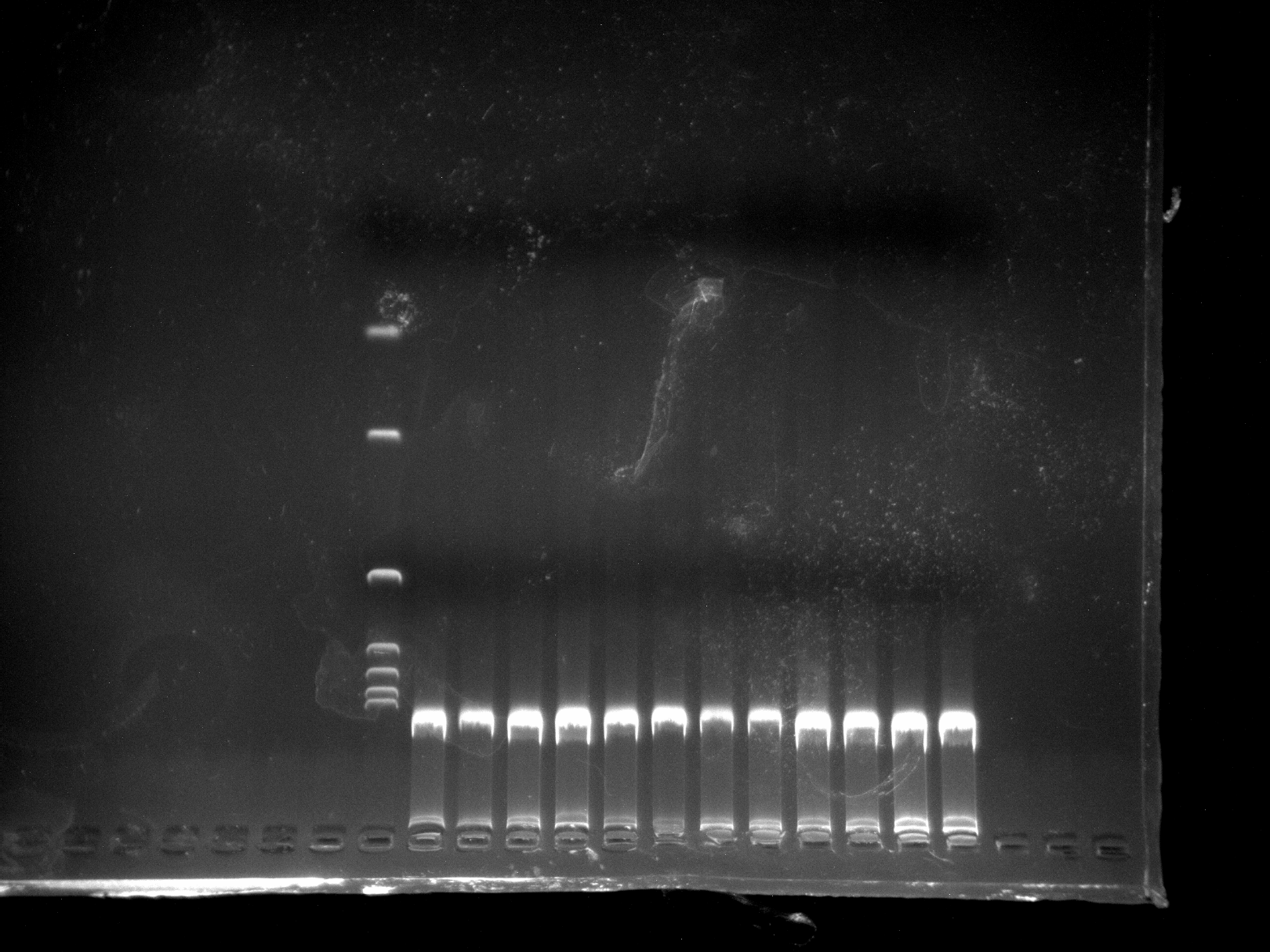

Supplement: Supplemental Information 98 — Two repetitions per sample. [file peerj-08-8498-s098.png]

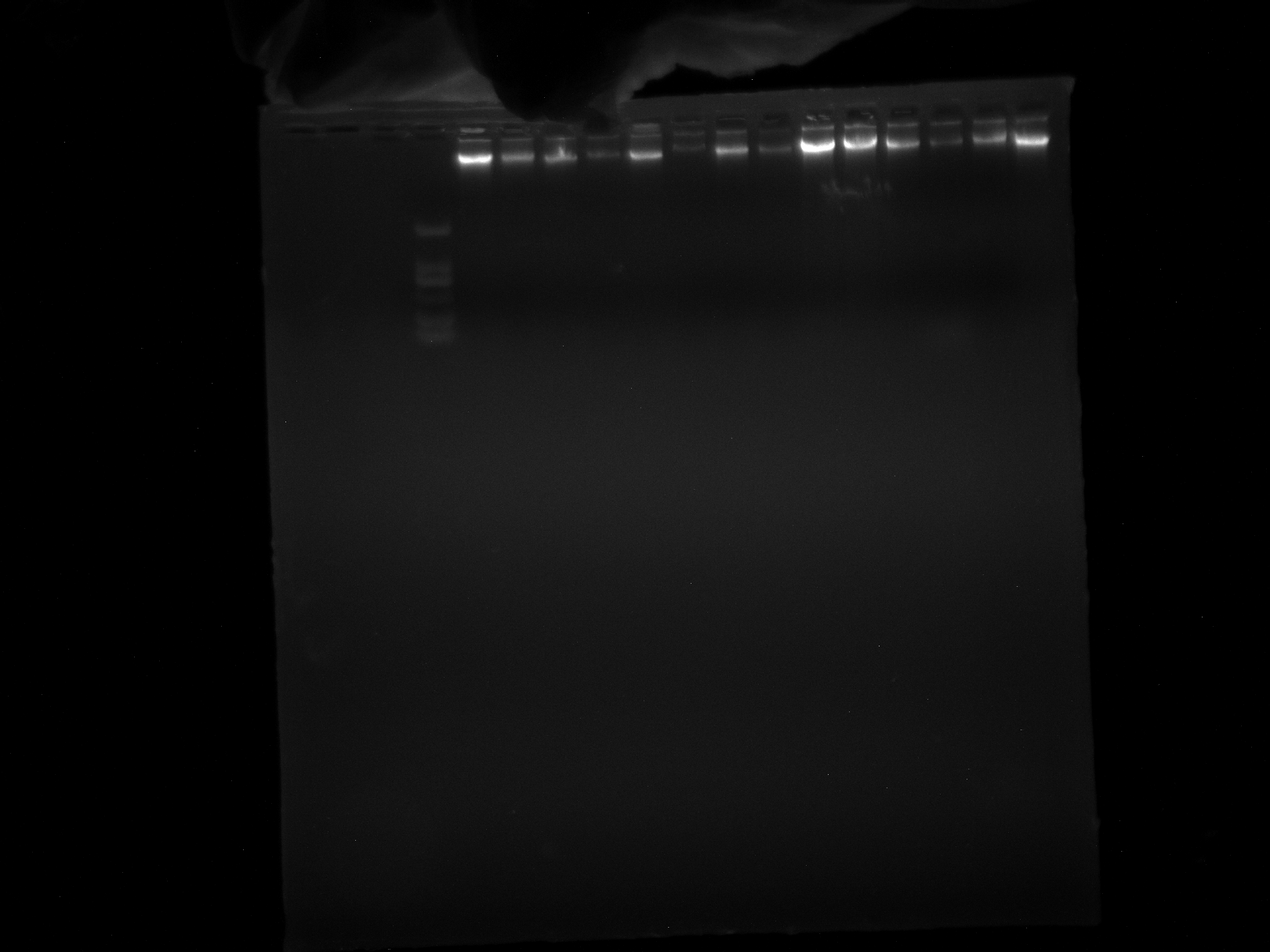

Supplement: Supplemental Information 99 — Two repetitions per sample. [file peerj-08-8498-s099.png]

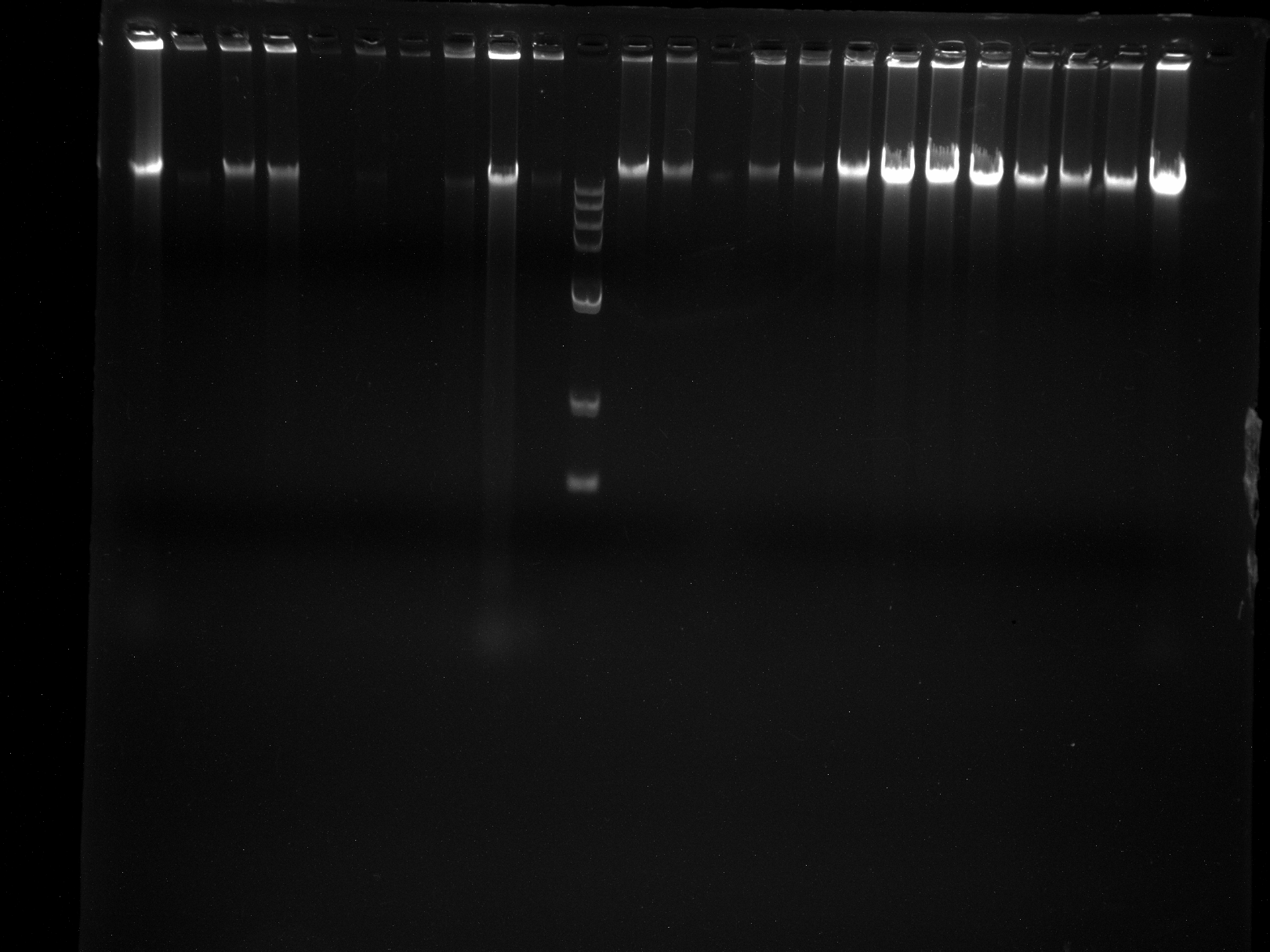

Supplement: Supplemental Information 100 — Two repetitions per sample. [file peerj-08-8498-s100.png]

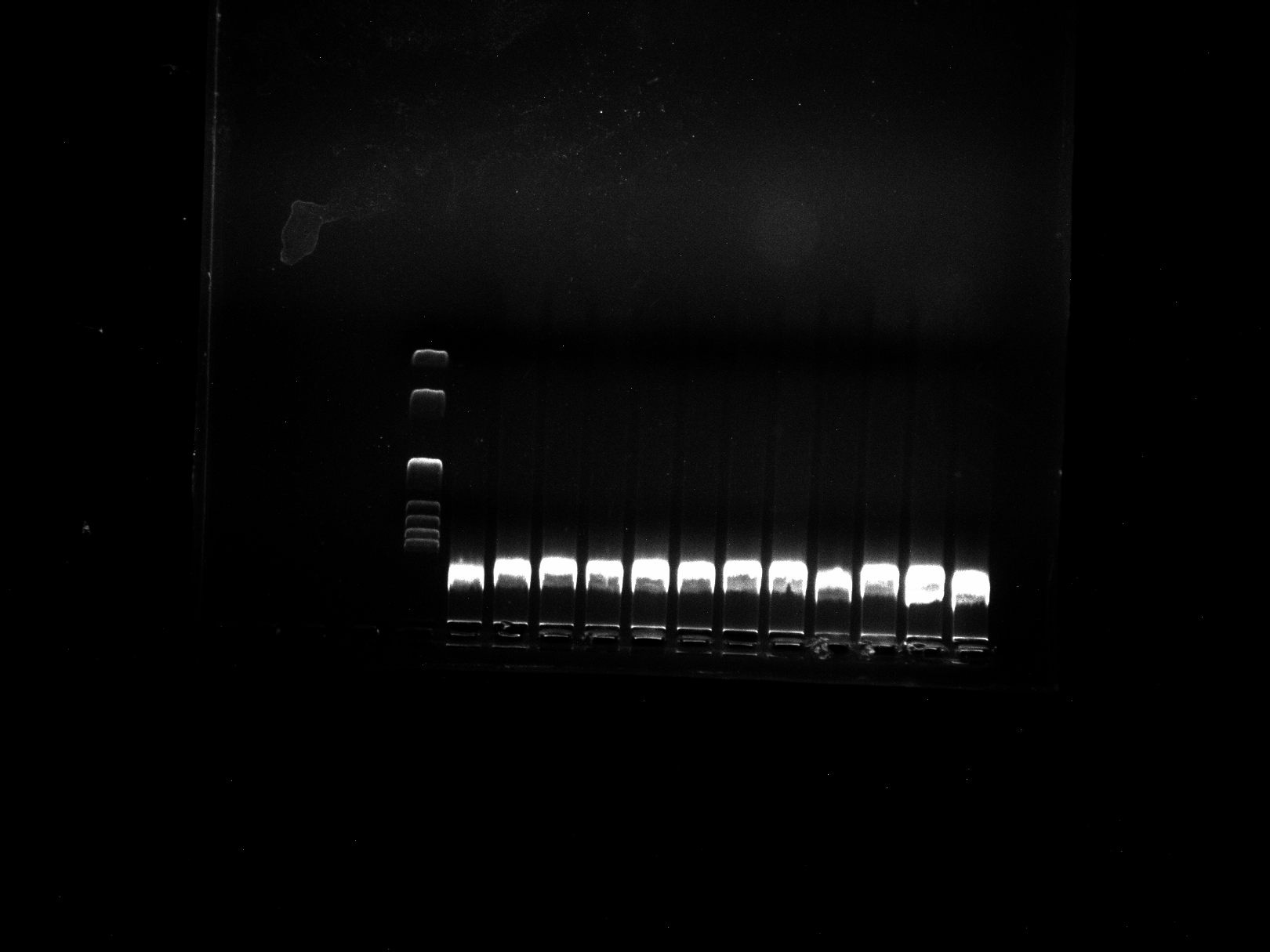

Supplement: Supplemental Information 101 — Two repetitions per sample. [file peerj-08-8498-s101.png]
