# Supplementary figures and images for: Analysis of the genetic diversity of the coastal and island endangered plant species Elaeagnus macrophylla via conserved DNA-derived polymorphism marker (part 2 of 2)
Source: PeerJ. 2020 Jan 31;8:e8498. doi: 10.7717/peerj.8498 (PMC6996508; doi:10.7717/peerj.8498)

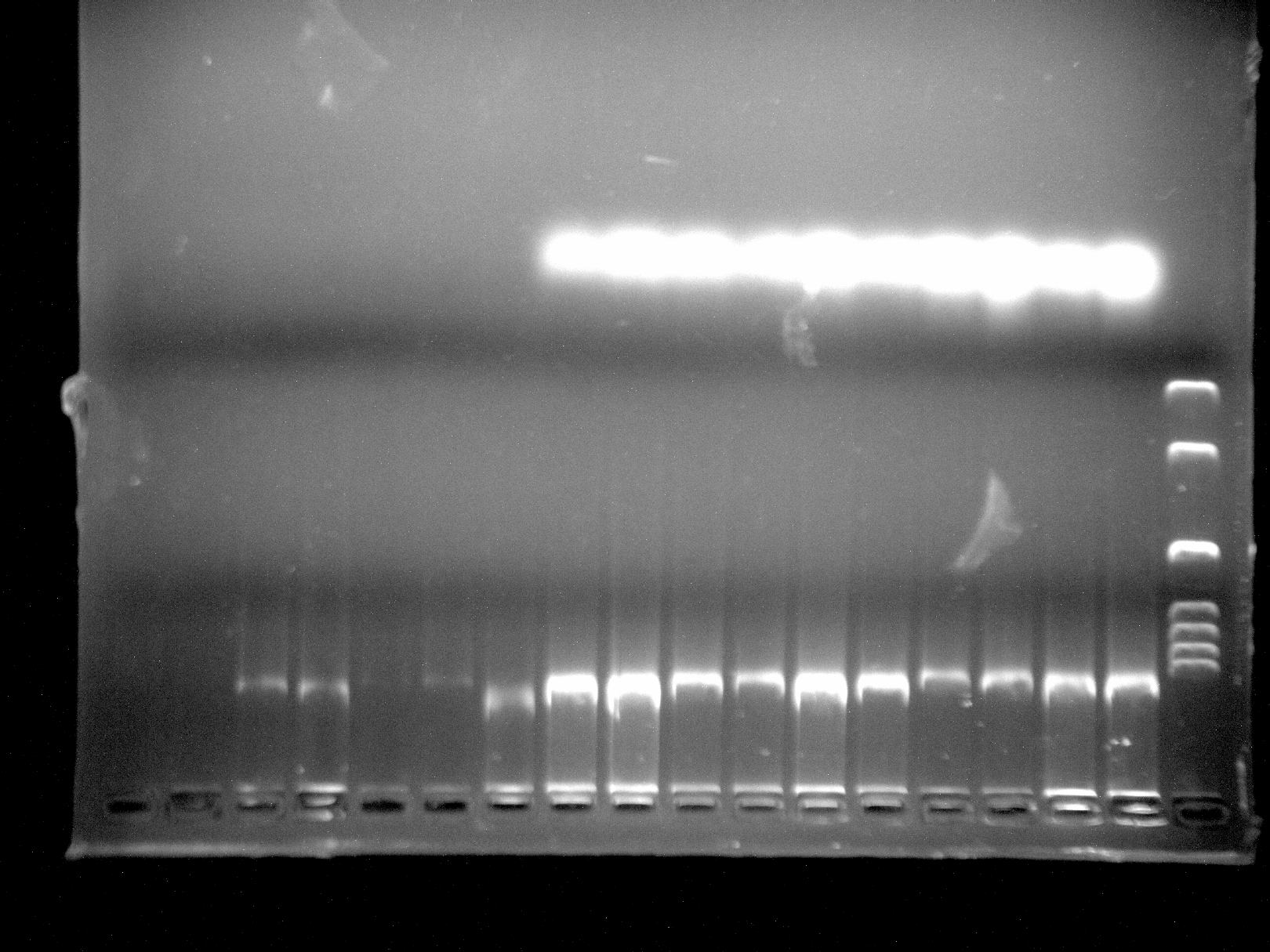

Supplement: Supplemental Information 102 — Two repetitions per sample. [file peerj-08-8498-s102.png]

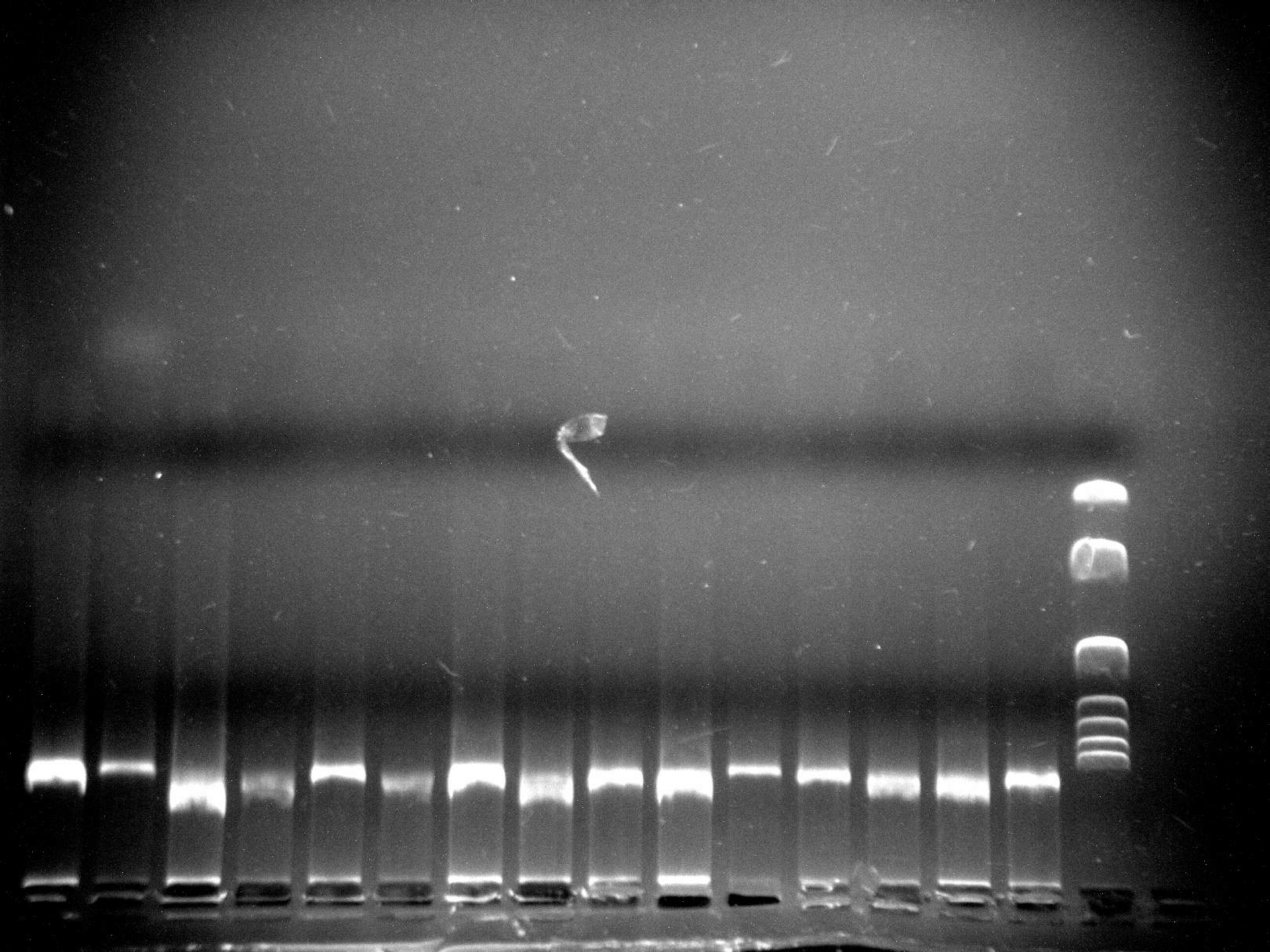

Supplement: Supplemental Information 103 — Two repetitions per sample. [file peerj-08-8498-s103.png]

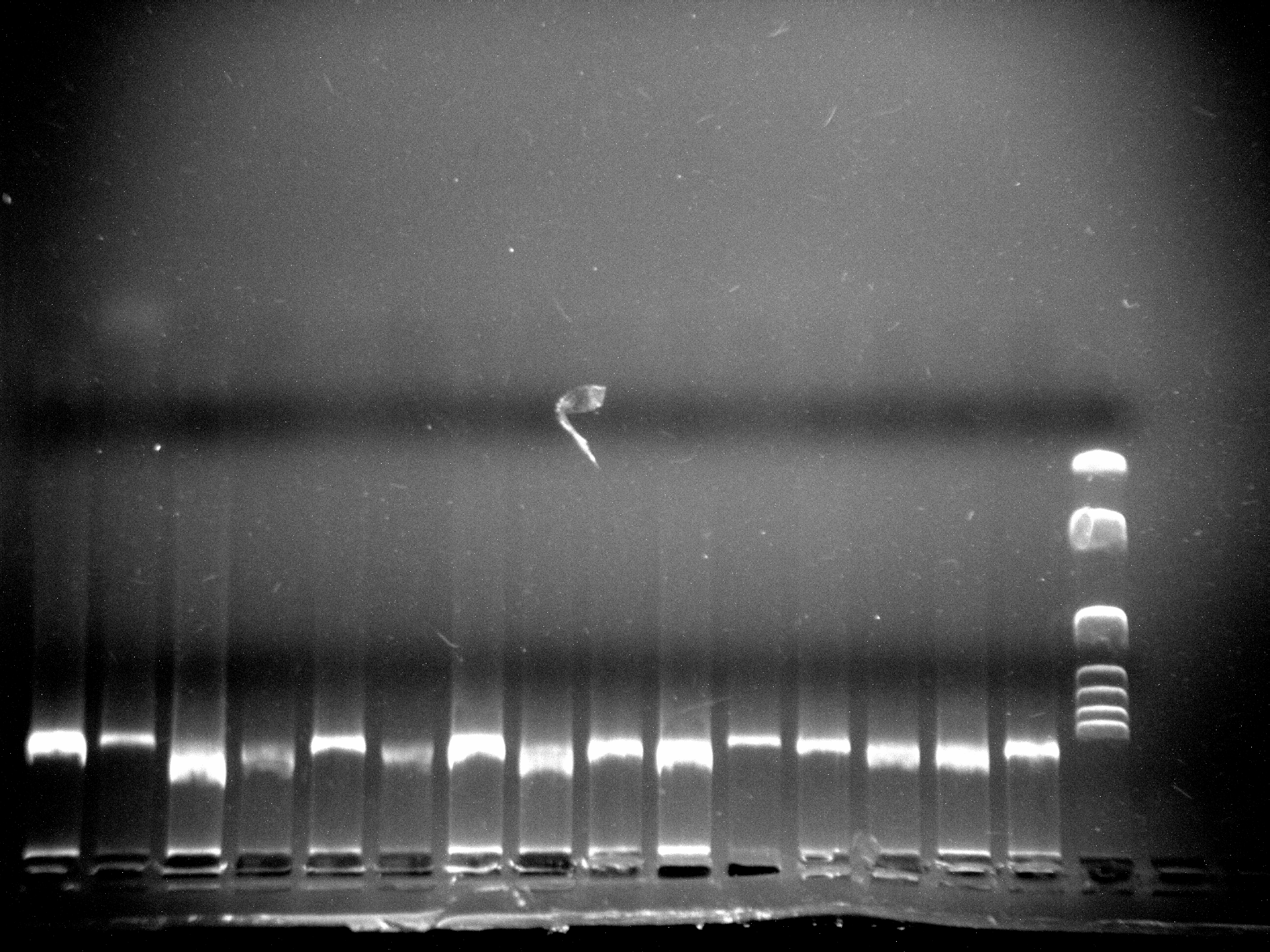

Supplement: Supplemental Information 104 — Two repetitions per sample. [file peerj-08-8498-s104.png]

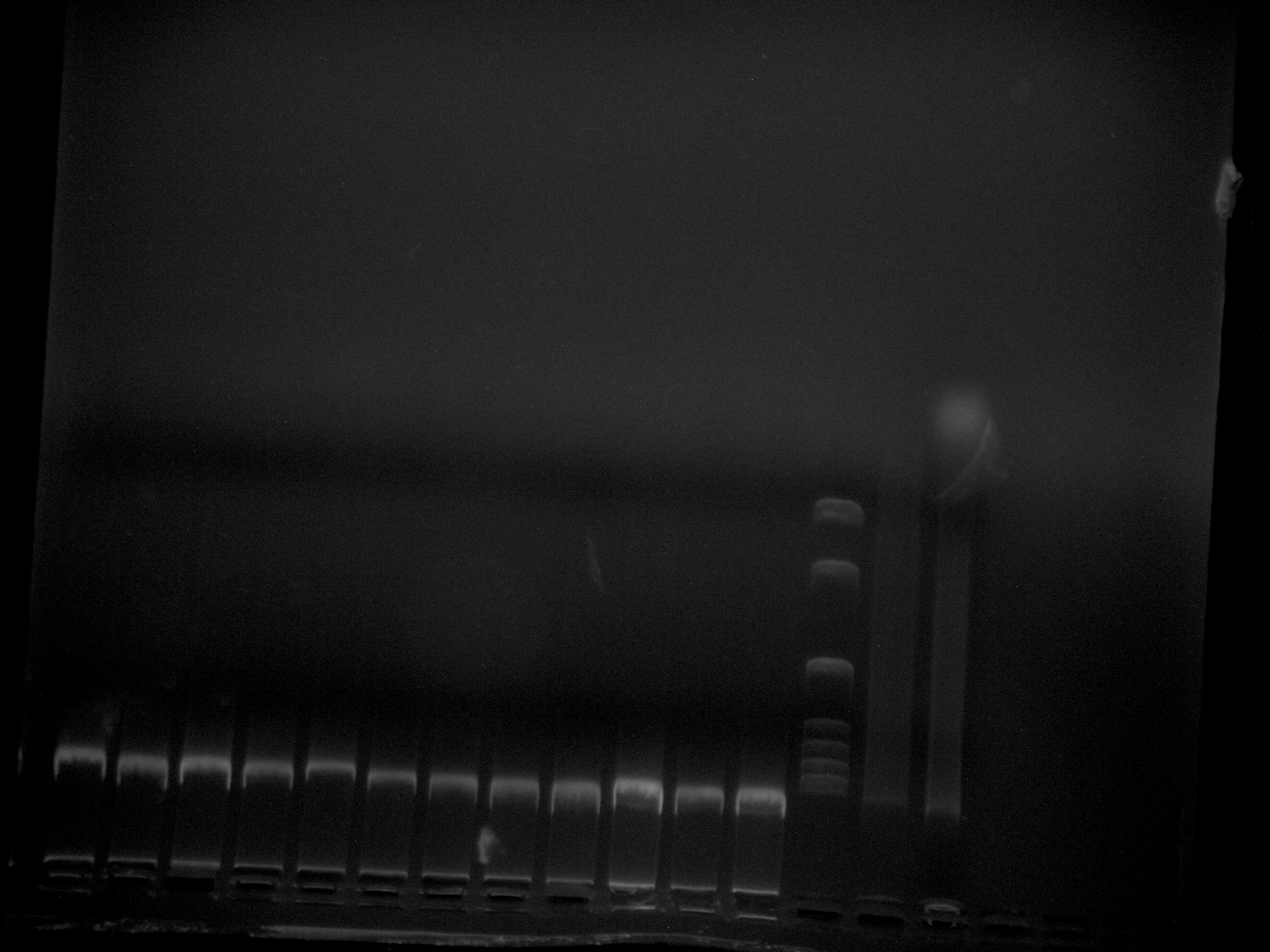

Supplement: Supplemental Information 105 — Two repetitions per sample. [file peerj-08-8498-s105.png]

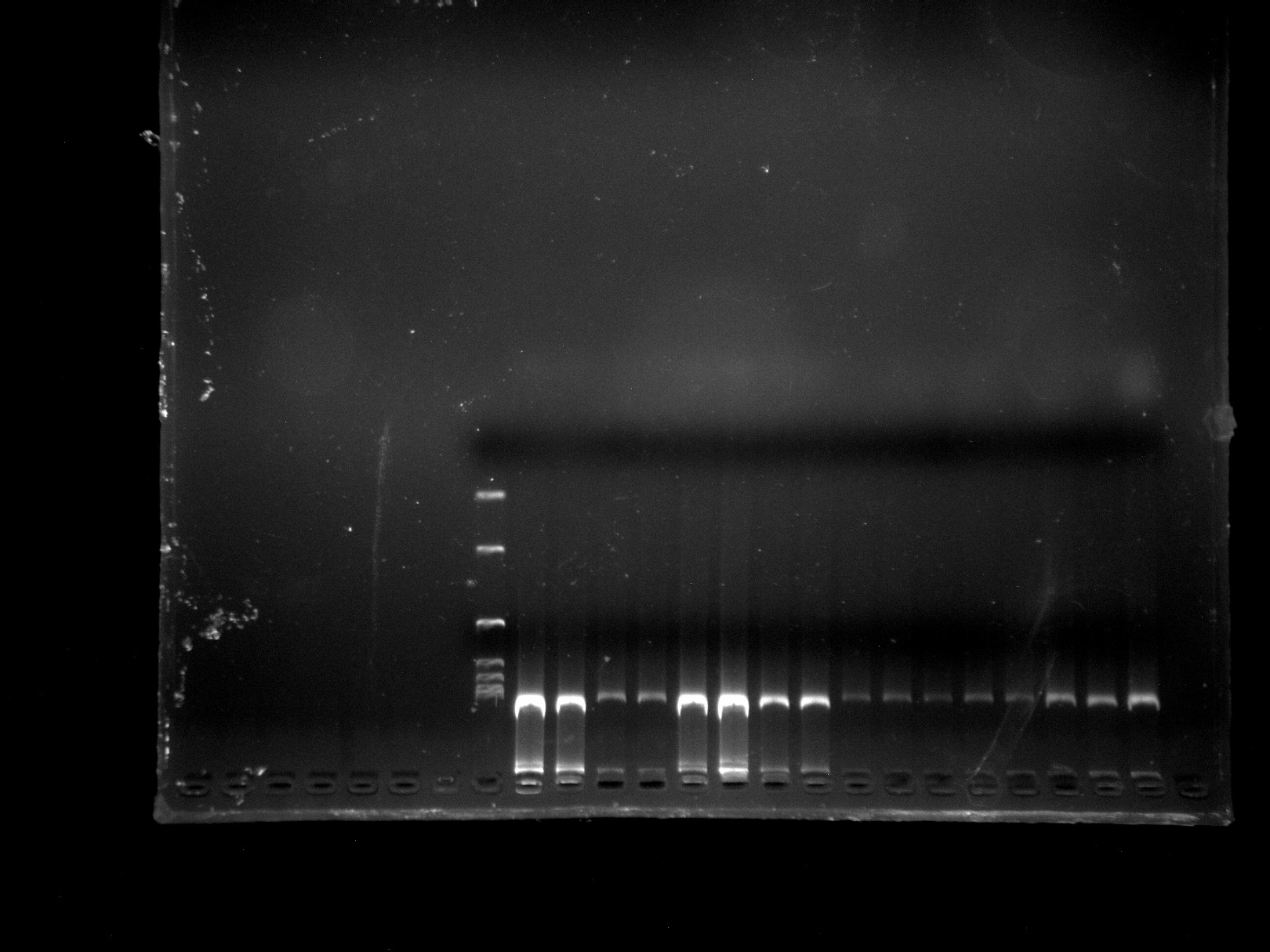

Supplement: Supplemental Information 106 — Two repetitions per sample. [file peerj-08-8498-s106.png]

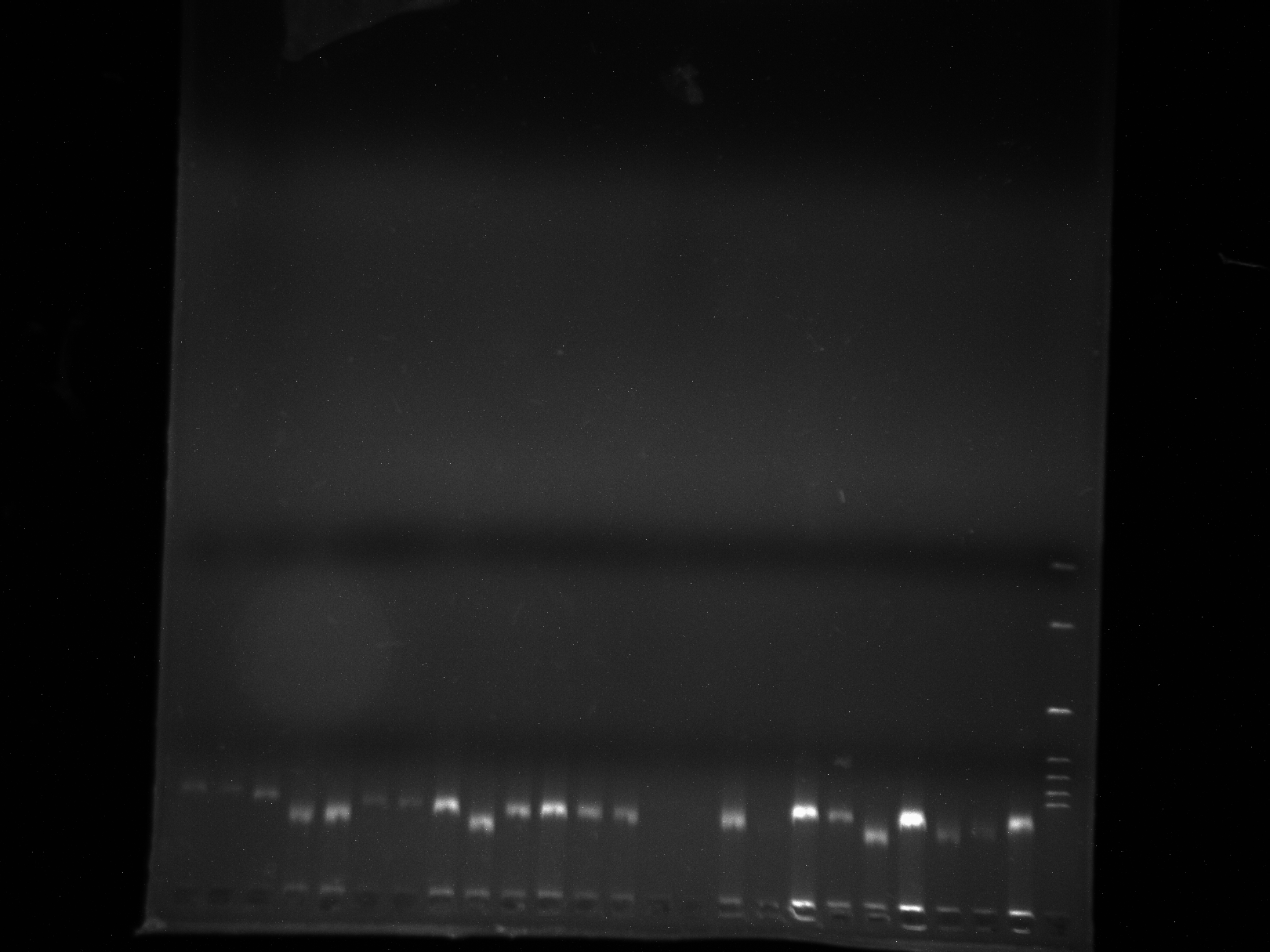

Supplement: Supplemental Information 107 — Two repetitions per sample. [file peerj-08-8498-s107.png]

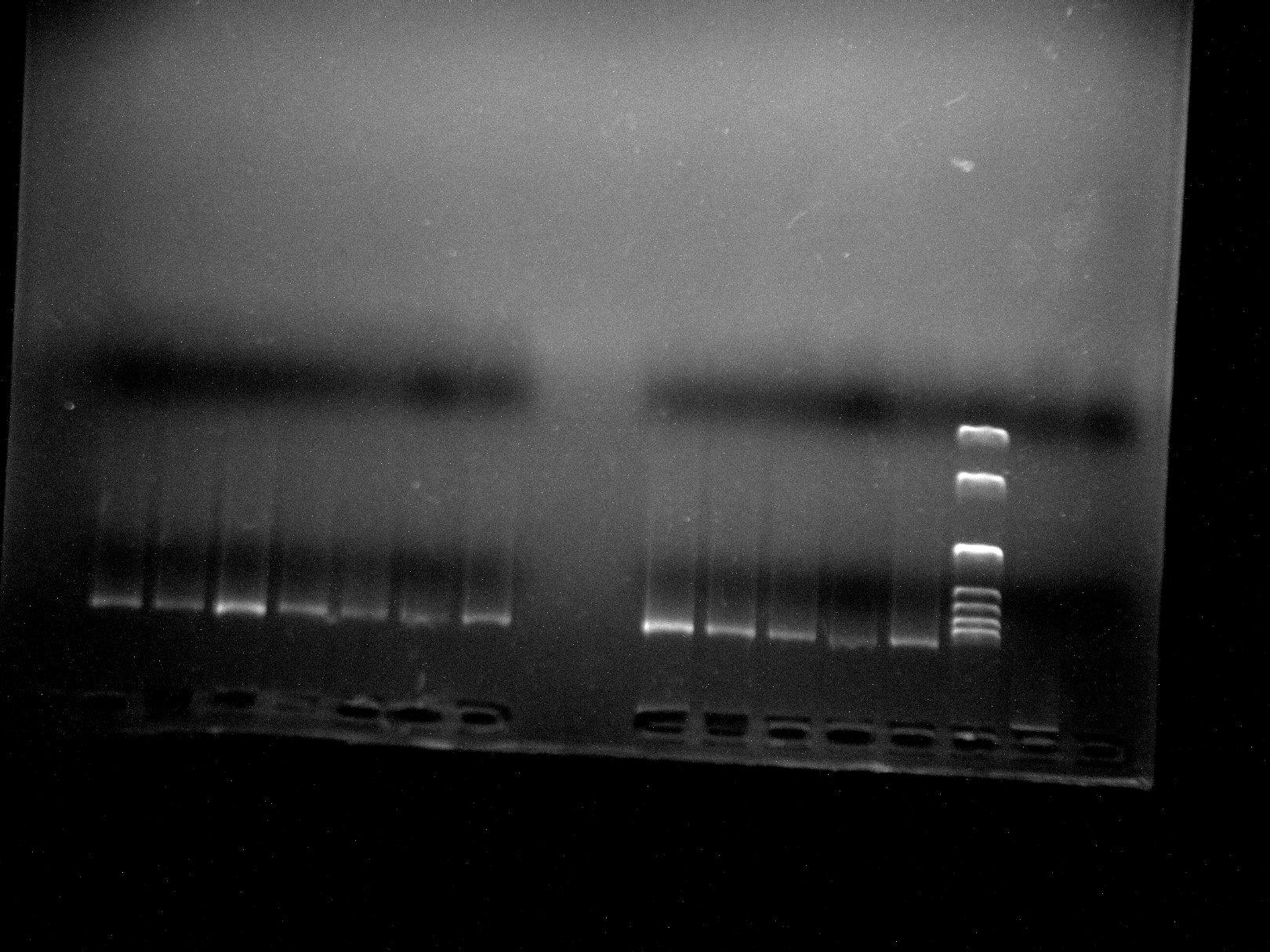

Supplement: Supplemental Information 108 — Two repetitions per sample. [file peerj-08-8498-s108.png]
